# Cerebrospinal fluid inflammatory biomarkers for disease progression in Alzheimer’s disease and multiple sclerosis: a systematic review

**DOI:** 10.3389/fimmu.2023.1162340

**Published:** 2023-07-13

**Authors:** Joke Temmerman, Sebastiaan Engelborghs, Maria Bjerke, Miguel D’haeseleer

**Affiliations:** ^1^ Vrije Universiteit Brussel, Center for Neurosciences (C4N), Jette, Brussels, Belgium; ^2^ Universiteit Antwerpen, Department of Biomedical Sciences and Institute Born-Bunge, Reference Center for Biological Markers of Dementia (BIODEM), Wilrijk, Antwerp, Belgium; ^3^ Universitair Ziekenhuis Brussel, Department of Neurology, Jette, Brussels, Belgium; ^4^ Universitair Ziekenhuis Brussel, Department of Clinical Biology, Laboratory of Clinical Neurochemistry, Jette, Brussels, Belgium; ^5^ National MS Center (NMSC), Neurology, Melsbroek, Steenokkerzeel, Belgium

**Keywords:** inflammation, biomarkers, Alzheimer’s disease (AD), multiple scleorsis (MS), disease progression

## Abstract

Inflammatory processes are involved in the pathophysiology of both Alzheimer’s disease (AD) and multiple sclerosis (MS) but their exact contribution to disease progression remains to be deciphered. Biomarkers are needed to define pathophysiological processes of these disorders, who may increasingly co-exist in the elderly generations of the future, due to the rising prevalence in both and ameliorated treatment options with improved life expectancy in MS. The purpose of this review was to provide a systematic overview of inflammatory biomarkers, as measured in the cerebrospinal fluid (CSF), that are associated with clinical disease progression. International peer-reviewed literature was screened using the PubMed and Web of Science databases. Disease progression had to be measured using clinically validated tests representing baseline functional and/or cognitive status, the evolution of such clinical scores over time and/or the transitioning from one disease stage to a more severe stage. The quality of included studies was systematically evaluated using a set of questions for clinical, neurochemical and statistical characteristics of the study. A total of 84 papers were included (twenty-five for AD and 59 for MS). Elevated CSF levels of chitinase-3-like protein 1 (YKL-40) were associated with disease progression in both AD and MS. Osteopontin and monocyte chemoattractant protein-1 were more specifically related to disease progression in AD, whereas the same was true for interleukin-1 beta, tumor necrosis factor alpha, C-X-C motif ligand 13, glial fibrillary acidic protein and IgG oligoclonal bands in MS. We observed a broad heterogeneity of studies with varying cohort characterization, non-disclosure of quality measures for neurochemical analyses and a lack of adequate longitudinal designs. Most of the retrieved biomarkers are related to innate immune system activity, which seems to be an important mediator of clinical disease progression in AD and MS. Overall study quality was limited and we have framed some recommendations for future biomarker research in this field.

**Systematic review registration:**

https://www.crd.york.ac.uk/prospero/, identifier CRD42021264741.

## 1 Introduction

Alzheimer’s disease (AD) and multiple sclerosis (MS) are two frequently occurring and debilitating disorders of the central nervous system (CNS). Whereas these conditions exhibit fundamental epidemiological and pathobiological differences, they also share a number of striking commonalities, making them suitable for an integrative approach in translational research. Cognitive decline is the core clinical feature of AD, usually starting with memory loss, language difficulties and visuospatial deficits (1). In MS, physical symptoms are often more visible (2), but impaired cognition (typically affecting information processing speed, episodic memory, attention and executive function), which may be subtle but therefore not necessarily less troublesome, has been reported in up to 70% of patients and can already be present from the early stages of the disease (3, 4). Neurodegeneration is the ultimate pathological mechanism leading to functional decline in both entities, accounting as the main determinant of global long-term prognosis in MS (5), and highly correlating with cognitive decline in individuals with AD (6). Inflammatory responses are involved as well, to varying degrees and as explained more into detail below, but their precise role in the respective neurodegenerative cascades remains to be elucidated (7). Interestingly, the life expectancy of individuals with MS has significantly improved over the past decades, probably due to ameliorated disease modifying treatments (DMT) and/or general medical care. As a consequence, and in combination with a rising prevalence of both disorders (8, 9), there is a growing likelihood of co-existence of MS with classic age-related dementias such as AD (10). Some studies have even reported an increased risk of AD diagnosis in patients with MS, as compared to controls (11–13). In order to enable high-quality precision medicine, it will become increasingly important to unravel the unique and (possibly) shared pathophysiological processes of both disorders. Biomarkers are vital when investigating disease mechanisms as they are the *in vivo* proxy of neuropathology, used in both medical routine and clinical trials to achieve formal (and early) diagnosis/patient stratification, predict disability progression and/or monitor treatment response (14, 15). In addition, biomarker research may lead to the identification of new druggable targets for disease modification, potentially leading to prevention of functional decline in affected individuals.

AD is classically viewed as a pure neurodegenerative disorder, characterized by the deposition of amyloid beta (Aβ)-containing plaques and neurofibrillary tangles composed of hyperphosphorylated tau protein in the brain. The earliest biomarker sign representing AD pathology is a reduced concentration of Aβ_1-42_ and a decreased Aβ_1-42_/Aβ_1-40_ ratio in the cerebrospinal fluid (CSF), of which the latter is the most specific for plaque pathology (16). Neuropathology may develop up to 20 years before the first manifestation of cognitive symptoms (17) and supports the concept that AD is a continuum with a long preclinical phase, followed by a prodromal phase of mild cognitive impairment (MCI) that eventually leads to dementia (18). Positron emission tomography (PET) studies have demonstrated an increased density of translocator protein (TSPO), a marker for neuroinflammation that is predominantly present in microglia, in patients with AD, as compared to healthy individuals (19). Disease-associated microglia (DAMs), a context-dependent microglia state identified by novel technologies, such as single-cell RNA sequencing (20), have been found in the vicinity of the amyloid plaques (21), while several well-established AD risk genes closely relate to microglial functions and appear to be highly enriched in these cells (22). As such, the innate inflammatory response may be implicated in the early pathological process in AD. However, the heterogenous presentation of AD has previously split the field concerning the role of inflammation and hindered the establishment of inflammatory biomarkers to monitor disease progression. It remains to be determined whether the inflammatory changes are actually a driving force behind the pathology or merely represent a bystander effect, and whether the inflammatory state is beneficial, by clearing pathological aggregates, or detrimental, by acting provocative for the degenerative process (23). Conversely, an inadequate immune response in AD may equally lead to disease progression, as microglia surrounding the plaques apparently fail to take part in phagocytosis (24). Anyhow, current literature does suggest that inflammatory CSF biomarkers are altered in AD (25), but the impact on progression across the disease continuum needs further elaboration.

MS is a neuroinflammatory and -degenerative disorder of the CNS with a causative mechanism that is incompletely understood but presumably autoimmune in origin. Most patients (85%) start with a relapsing-remitting (RR) pattern during which abrupt and at least partially recovering exacerbations of neurological dysfunction (termed relapses) are interchanged with periods of clinical stability. Many will eventually transit into a secondary progressive (SP) phase characterized by a slower but sustained downhill course that may still be accompanied by some relapses, whereas about 15% of patients experience a similar primary progressive (PP) decline from disease onset (2). Focal demyelinating lesions, resulting from acute inflammatory activity with blood-brain barrier (BBB) disruption and perivenular infiltrates of peripheral immune cells (including T-cells, B-cells, plasma cells and macrophages), form the pathological substrate for relapses (2). Recent insights have learned that progressive MS is more likely to rely, among other mechanisms, on innate immune processes behind an intact BBB. Microglia - more specifically microglia inflamed in MS (MIMS; whose transcriptional profile partially overlaps with DAMs) (20, 26) - have been found diffusely throughout the normal-appearing white matter (NAWM) and at the border of a particular subset of chronic slowly expanding lesions (also called chronically active or smoldering lesions), both of which are features increasingly associated with the progressive phase of the disease (27–29). Increased TSPO expression in the brain of patients with MS was found to predict future disability progression and several MS susceptibility genes are enriched in microglia, suggesting an important role of the innate immune response in MS (30, 31). However, similar to the potential double-edged role in AD, microglia also facilitate neuronal repair by clearing debris and stimulating remyelination (32). Indications of MS pathology presumably appear long before clinical diagnosis, as evidenced by various alterations at the clinical, biochemical and/or radiological level (33–37). Some of these prodromal circumstances are well-defined by the terms ‘clinically and radiologically isolated syndrome’ in case of first inflammatory demyelinating episode without proof of dissemination in time (CIS) or when magnetic resonance imaging (MRI) findings are strongly suggestive for MS but without clinical repercussions (RIS), respectively. Despite the identification of the aforementioned inflammatory players in MS pathology, it remains to be seen how they may translate to disease progression biomarkers. Thus far, only oligoclonal bands (OCB), which are the product of intrathecal antibody secretion by activated B-cells, have been included in the official criteria for MS diagnosis (38).

So far, drug development mainly focused on disease-specific characteristics, such as the removal of Aβ plaques in AD and altering the peripheral immune response in MS, but fails to fully cease disease progression (39, 40). Improving our understanding of the complex relationship between neuroinflammation and neurodegeneration in both disorders, which may have more in common than hitherto thought, can be valuable to uncover therapeutic targets that may truly halt disease progression. Notwithstanding the progress that recently has been made in the field of body fluid-based inflammatory biomarker research in AD and MS, its implementation in the real-world clinical practice turns out to be a slow process. With this systematic review, we have listed all CSF inflammatory biomarkers showing an association with clinical disease progression in AD and/or MS patients, in an attempt to identify the most promising candidates for further validation in clinical practice and to gain a deeper understanding in the inflammatory pathways underlying disease progression, which may ultimately culminate in ameliorated clinical care for patients affected by these chronic disorders. Moreover, we have found several methodological and conceptual commonalities in biomarker research for AD and MS, and summarize some general key points to enhance the quality of future efforts studying the relationship with disease progression.

## 2 Methods

### 2.1 Research question

We formulated our research question and conceptualized the search terms by using the ‘Patient – Intervention – Comparator – Outcome – Time – Setting (PICOTS) framework’ (41). Our aim was to provide a structured overview of the inflammatory biomarkers in the CSF (I) of patients belonging to the AD and/or MS continuum (P) that have been associated with disease progression (O), as opposed to situations of clinical stability (C), in cross-sectional and/or longitudinal (T) clinical studies (S). Papers were only found suitable for inclusion if disease progression was measured using (a) clinically validated tests representing baseline functional and/or cognitive status, (b) the evolution of such clinical scores over time and/or (c) the transitioning from one (possibly prodromal) disease stage to a more severe stage (i.e., MCI to AD; CIS or RIS to clinically definite MS; RR MS to SP MS). These criteria were also used as the core structure for grouping the retrieved papers, which allows a consistent synthesis of the results. Our review was conducted following the guidelines of the Preferred Reporting Items for Systematic Review and Analyses (PRISMA) (42), we refer to the **Supplementary Material** for the related checklist (**Table S1**). The protocol was submitted to the PROSPERO database (Internal Prospective Register of Systematic Reviews; registration number: CRD42021264741), maintained by the Centre for Reviews and Dissemination at the University of York (Heslington, UK), to help avoid duplicate efforts.

### 2.2 Search strategy

International peer-reviewed literature relevant to our PICOTS research question was screened up to March 24, 2022 using the PubMed and Web of Science databases. To be more inclusive, we developed separate search strategies for AD and MS. Medical Subject Heading search terms were entered in all fields of publication (e.g., abstract, title, keywords); the final search queries are presented in **Table 1**. Title and abstract from retained papers were subsequently evaluated for eligibility, with additional reading of the entire text in case of non-exclusion. Studies were considered suitable for inclusion and data collection only if they were written in English, were performed in humans and investigated the relationship between CSF inflammatory biomarkers and disease progression in AD and/or MS patients; the latter in accordance with the above described definition. Reviews, case reports and case series were rejected; there were no other exclusion criteria.

**Table 1:** Search query for systematic review.

	MS/AD	Search query	Results
**PubMed**	MS	(“CSF” OR “cerebrospinal fluid” OR “Biomarkers/cerebrospinal fluid”[Mesh]) AND (“multiple sclerosis” OR “MS” OR “Multiple Sclerosis”[Mesh] OR “CIS” OR “clinically isolated syndrome” OR “RIS” OR “radiologically isolated syndrome” OR “RRMS” OR “relapsing?remitting multiple sclerosis” OR “SPMS” OR “secondary progressive multiple sclerosis” OR “PPMS” OR “primary progressive multiple sclerosis” OR “Converters” OR “Non-converters”) AND (neuro?inflammat* OR “Inflammation”[Mesh]) AND (“predictive value” OR “prognostic value” OR “progression” OR “Predictive Value of Tests”[Mesh] OR “disease progression” OR “Disease Exacerbation” OR “Disease Progression”[Mesh] OR “functional decline” OR “cognitive decline” OR “cognitive dysfunction” OR “Cognitive Dysfunction”[Mesh] OR “neurodegeneration” OR “Nerve Degeneration”[Mesh])	364
	AD	(“CSF” OR “cerebrospinal fluid” OR “Biomarkers/cerebrospinal fluid”[Mesh]) AND (“alzheimer disease” OR “alzheimer’s disease” OR “Alzheimer Disease”[Mesh] OR “mild cognitive impairment” OR “MCI” OR “Prodromal” OR “Preclinical” OR “AD”) AND (neuro?inflammat* OR “Inflammation”[Mesh]) AND (“predictive value” OR “prognostic value” OR “progression” OR “Predictive Value of Tests”[Mesh] OR “disease progression” OR “Disease Exacerbation” OR “Disease Progression”[Mesh] OR “functional decline” OR “cognitive decline” OR “cognitive dysfunction” OR “Cognitive Dysfunction”[Mesh] OR “neurodegeneration” OR “Nerve Degeneration”[Mesh])	258
**Web of Science**	MS	**TS**= (‘multiple sclerosis’ OR ‘MS’ OR ‘RRMS’ OR ‘relapsing?remitting multiple sclerosis’ OR ‘SPMS’ OR ‘secondary progressive multiple sclerosis’ OR ‘PPMS’ OR ‘primary progressive multiple sclerosis’ OR ‘CIS’ OR ‘clinically isolated syndrome’ OR ‘RIS’ OR ‘radiologically isolated syndrome’) *AND* **TS**=(cerebrospinal fluid) *AND* **TS**=(‘prognostic value’ OR ‘disease progression’ OR ‘progression’ OR ‘predictive value’) *AND* **TS**=(inflam*)	491
	AD	**TS**=(‘alzheimer* disease’ OR ‘AD’ OR ‘MCI’ OR ‘mild cognitive impairment’ OR ‘Prodromal’ OR ‘Preclinical’) *AND* **TS**=(cerebrospinal fluid) *AND* **TS**=(‘prognostic value’ OR ‘disease progression’ OR ‘progression’ OR ‘predictive value’) *AND* **TS**=(inflam*)	344

AD, Alzheimer’s disease; MS, multiple sclerosis.

**Figure 1:**
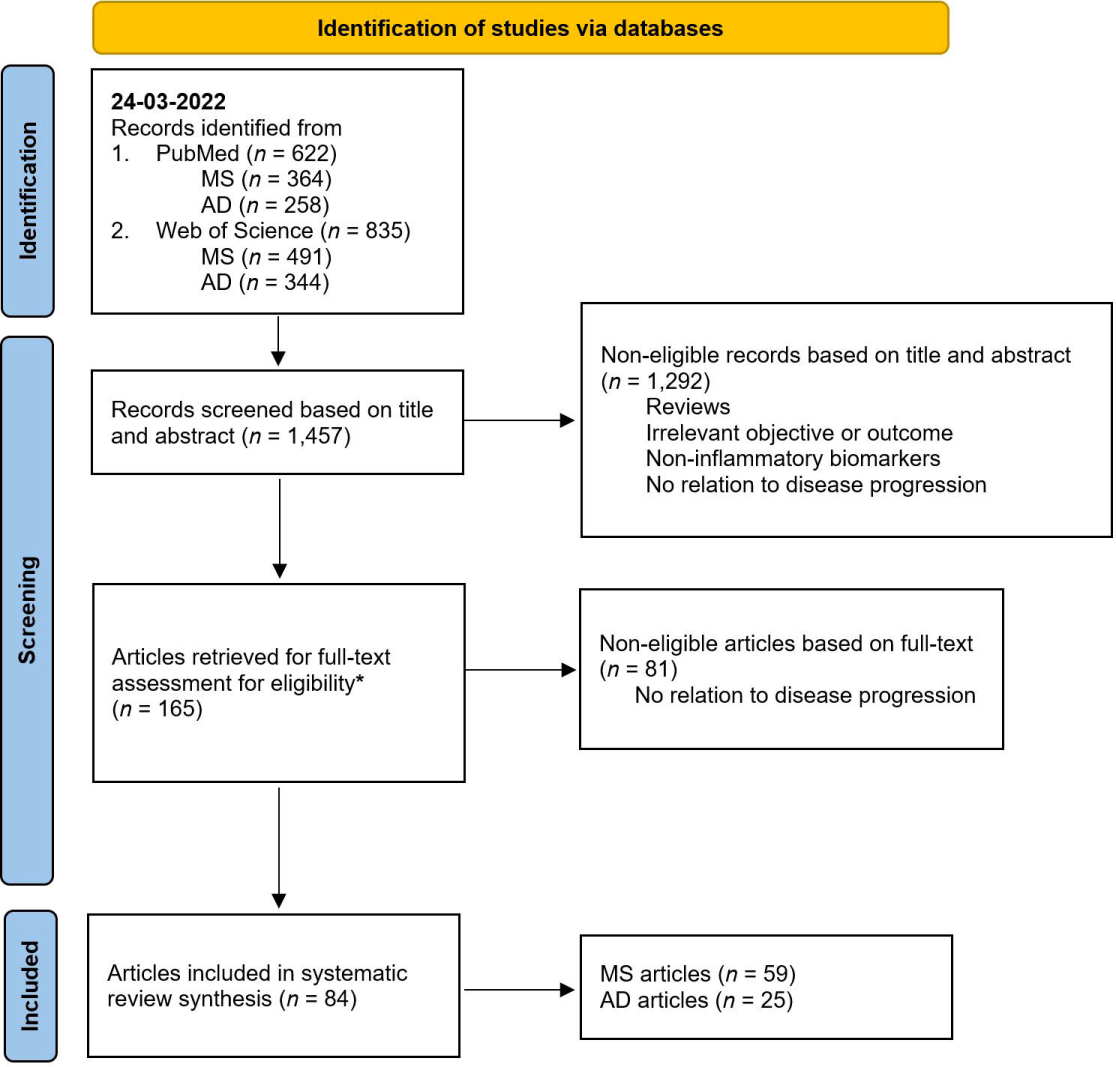
PRISMA 2020 flow chart diagram for systematic reviews. AD, Alzheimer’s disease; MS, multiple sclerosis. * From the 165 papers, 55 raised doubts for inclusion after full-text assessment. These papers were double-checked by all the authors. In the end, 19 papers were included after reaching a consensus, while the remaining 36 were excluded. Illustration adapted from Page et al., 2021 (42).

Search and selection procedures were performed by a single reviewer (J.T.); for flowchart see **Figure 1**. Title and abstract screening resulted in 165 candidate papers. Following full-text evaluation, 84 papers (all published between 1991 and 2022) complied to our criteria and were included for data extraction and quality assessment. More than double the number of papers were found in the field of MS (*n* = 59) compared to AD (n = 25). Uncertainties during the search were resolved by consensus after a discussion involving all authors.

### 2.3 Data extraction

Data extraction was performed in a standardized manner by J.T. and M.B. and consisted of the following items: first author, year of publication, journal, study aim, characteristics of the studied cohorts (diagnostic criteria, subgroups, age, clinical status), neurochemical quality measures (assay, intra-assay coefficient of variation, limit of detection, detectability rate, blinding of the researcher), inflammatory biomarkers of interest, observed median and/or mean levels of biomarkers in the studied cohorts, measures of disease progression (with follow-up data in case of longitudinal studies), statistical analyses and main findings. A composite evidentiary Excel spreadsheet (Microsoft Corp, Redmond, WA, USA) compiling all variables was created to ensure reproducibility and completeness of the dataset. The complete overview of the collected data can be found in the Supplementary Material (**Table S2**).

### 2.4 Quality assessment

We developed a set of questions to systematically evaluate the methodological quality of each included study, focusing on diagnostic criteria, neurochemical analysis, clinical scoring, cohort size, statistics, validation of findings and, in case of longitudinal studies, duration of follow-up. Separate questions for AD and MS papers were formulated for aspects that were considered disease-specific. The quality assessment was conducted independently by J.T. and M.B., and variations were discussed between both authors until a consensus was reached. Publications with a grade below 50%, from 50% to 80%, and above 80% of the potential maximum score were categorized as “Low”, “Moderate” or “High” in quality, respectively. The quality assessment for all included papers (*n* = 84) can be found in the Supplementary Material (**Table S3**). Although most papers were of low quality, none were excluded based on this criterion, in order to be able to provide a complete overview of the literature.

## 3 Results

### 3.1 Inflammatory biomarkers related to clinical status

#### 3.1.1 General overview

Of the 84 papers selected for data extraction, 43 focused on the cross-sectional relationship between inflammatory biomarkers and clinically validated scores representing functional status and/or cognition collected at baseline (AD/MS = 14/29). The Mini-Mental State Examination (MMSE) and Expanded Disability Status Scale (EDSS), which are measures of global cognitive impairment and general disability, respectively (43, 44), were the most frequently used scales in AD (MMSE: 85.7%) and MS (EDSS: 96.6%) studies. The vast majority of relationships were assessed using correlation analyses; a detailed overview of these results can be found in the Supplementary Material (**Table S4**).

#### 3.1.2 AD literature

Cognitive testing in AD studies included MMSE, the Clinical Dementia Rating (CDR), the Alzheimer’s Disease Assessment Scale – Cognitive Subscale (ADAS-Cog), Dementia Rating Scale-2 (DRS-2), the Frontal Assessment Battery (FAB), the Consortium to Establish a Registry for Alzheimer’s Disease (CERAD) neuropsychological battery and the Montreal Cognitive Assessment (MoCA) (45–50). The examined cohorts consisted either of MCI or dementia due to AD patients, with a sample size varying from 19 to 163 individuals.

From the 33 inflammatory biomarkers that were studied, only eight were significantly associated with cognitive impairment: activated helper and cytotoxic T-cells, monocyte chemoattractant protein-1 (MCP-1 or CCL2), C-X-C motif chemokine ligand 8 and 10 (CXCL8 and CXCL10, respectively), interleukin-1 beta (IL-1β), macrophage inflammatory protein-1 alpha (MIP-1α) and osteopontin (OPN) (**Table 2A**). However, consistent results across multiple studies were only found for OPN (which is a cytokine with regulatory effects on the function of microglia (51)), demonstrating an overall moderate correlation with baseline MMSE scores in subjects with AD (52, 53). These findings were mainly based on cohorts with lower mean MMSE values and older age, likely representative for a more advanced disease stage.

**Table 2A:** Overview inflammatory biomarkers significantly associated with baseline clinical scores in AD publications.

Biomarker	Study	Quality	Cohort (n)	Age (y)	MMSE	Clinical measure	Correlation coefficient
Activated CD4+ T-cells	*Lueg, 2015*	L	AD (54)	68.7	21	Verbal learning *(n = 46)*	**-0.441**
						Verbal retrieval *(n = 46)*	**-0.456**
						Visuospatial skills *(n = 46)*	-0.364
Activated CD8+ T-cells	*Lueg, 2015*	L	MCI (19)	68.0	26	Verbal learning *(n = 15)*	**-0.545**
						Verbal retrieval *(n = 15)*	-0.298
						Visuospatial skills *(n = 15)*	**-0.523**
			AD (54)	68.7	21	Verbal learning *(n = 46)*	**-0.371**
						Verbal retrieval *(n = 46)*	**-0.744**
						Visuospatial skills *(n = 46)*	**-0.334**
MCP-1 (CCL2)	*Kimura, 2018*	M	AD (69)	73.6	23	MMSE	**-0.245**
						FAB	**-0.306**
	*Correa, 2011*	L	AD (22)	74.7	16	MMSE	NR
	*Galimberti, 2006*	L	EOAD (22) LOAD (14)	59.4 72.4	16 19	MMSE *(EO+LO)*	**0.350**
CXCL8 (IL-8)	*Kimura, 2018*	M	AD (69)	73.6	23	FAB	**-0.293**
CXCL10	*Kimura, 2018*	M	AD (69)	73.6	23	MMSE	-0.183
						FAB	**-0.255**
	*Correa, 2011*	L	AD (22)	74.7	16	MMSE	NR
	*Galimberti, 2006*	L	EOAD (22) LOAD (14)	59.4 72.4	16 19	MMSE *(EO + LO)*	**0.370**
IL-1β	*Rui, 2021*	M	aMCI (33)	63.6	18	MMSE	**-0.357**
						MoCA	**-0.373**
			AD (33)	65.7	13	MMSE	**-0.486**
						MoCA	**-0.499**
	*Rizzi, 2017*	L	aMCI (33)	68.0	NR	CERAD	**0.299**
	*Hesse, 2016*	L	AD (NR)	68.0	22	MMSE	**-0.330**
	*Tarkowski,2003*	L	MCI (56)	72.0	29	MMSE *(n = 6)*	**0.460**
MIP-1β	*Taipa, 2019*	M	AD (32)	62.7	18	DRS-2	**0.429**
OPN	*Sun, 2013*	M	Newly AD (17) Chronic AD (18)	73.0 74.0	23 19	MMSE *(Newly + Chronic)*	**0.530**
	*Comi, 2010*	L	AD (67)	70.0	22	MMSE	**0.580**

AD, Alzheimer’s disease; (a)MCI, (amnestic) Mild Cognitive Impairment; CERAD, Consortium to Establish a Registry for Alzheimer’s disease neuropsychological battery; DRS-2, Dementia Rating Scale-2; EO(AD), Early Onset Alzheimer’s disease; FAB, Frontal Assessment Battery; H, High quality; L, Low quality; LO(AD), Late Onset Alzheimer’s disease; M, Moderate quality; MMSE, Mini-Mental State Examination; MoCA, Montreal Cognitive Assessment; NR, not reported. Significant p-values are marked in bold.

#### 3.1.3 MS literature

In total, 93 inflammatory biomarkers were evaluated for cross-sectional associations with clinical scores, including measures of general disability (EDSS), rate/severity of disability (Multiple Sclerosis Severity Score, MSSS; Age-Related Multiple Sclerosis Severity, ARMSS; Progression Index, PI; Multiple Sclerosis Disease Severity Scale, MS-DSS; Bayesian Risk Estimate for Multiple Sclerosis, BREMS) and fatigue (Fatigue Severity Scale, FSS) (54–59). The cohorts used for correlation analyses included between 16 and 244 subjects, but RR, SP and PP MS patients were often grouped together and considered as representative for the entire MS spectrum. Only 15 biomarkers demonstrated significant clinical relevance, with the majority describing a positive yet weak correlation with one or more of the clinical scores (**Table 2B**). More specifically, anti-neurofilament light chain (NF-L) antibodies, chitinase 3-like protein 1 (CHI3L1 or YKL-40), complement factor 3 (C3), glial fibrillary acidic protein (GFAP) and tumor necrosis factor alpha (TNF-α) were investigated by various independent studies, yet none of these biomarkers consistently showed significant results.

**Table 2B:** Overview inflammatory biomarkers significantly associated with baseline clinical scores in MS publications.

Biomarker	Study	Quality	Cohort (n)	Age (y)	EDSS	Clinical measure	Correlation coefficient
15(S)-PGF2α / 20:4 µg	*Lam, 2016*	L	RRMS (23) SPMS (24) PPMS (15)	35.9 49.4 55	1.5 5.8 4.0	EDSS *(RR + SP + PP)*	**0.313**
15(S)-PGF2α / CSF µg	*Lam, 2016*	L	RRMS (23) SPMS (24) PPMS (15)	35.9 49.4 55	1.5 5.8 4.0	EDSS *(RR + SP + PP)*	**0.280**
Anti-NF-L antibodies	*Ehling, 2004*	L	MS (130)	42.1	3.7	EDSS	NR
	*Silber, 2002*	L	RRMS (38) SPMS (18) PPMS (10)	29 28 41	NR NR NR	EDSS *(RR + SP + PP: n = 49)*	**0.620**
Anti-NF-H antibodies	*Silber, 2002*	L	RRMS (38) SPMS (18) PPMS (10)	29 28 41	NR NR NR	EDSS *(RR + SP + PP: n = 49)*	**0.510**
Anti-tubulin antibodies	*Silber, 2002*	L	RRMS (38) SPMS (18) PPMS (10)	29 28 41	NR NR NR	EDSS *(RR + SP + PP: n = 49)*	**0.340**
B-cell / monocyte ratio	*Cepok, 2001*	L	RRMS (21) SPMS (6) PPMS (4)	NR NR NR	NR NR NR	EDSS *(RR + SP + PP)*	NR
						PI *(RR + SP + PP)*	**0.570**
						PI *(RR + SP)*	0.590
						PI *(RR)*	0.630
YKL-40 (CHI3L1)	*Huss, 20 20*	M	RRMS (47) PMS (39)	34 53	2.0 6.0	EDSS *(RR)*	0.140
						EDSS *(P)*	-0.050
	*Gil-Perotin, 2019*	M	RRMS (99) SPMS (35) PPMS (23)	35 45 51	2.0 5.5 5.0	EDSS *(RR + SP + PP)*	**0.210**
	*Novakova, 2017*	M	RRMS (59)	37	2.5	EDSS	**0.274**
						MSSS	**0.297**
Complement factor C3	*Aeinehband, 2017*	L	MS (48)	41.2	3.0	EDSS	**0.170**
						MSSS	**0.130**
	*Sladkova, 2011*	L	CIS (20)	34.9	NR	EDSS	NR
GFAP	*Azzolini, 2022*	M	RRMS (51)	36.5	1.5	EDSS	NR
						BREMS	**0.501**
	*Abdelhak, 2018*	H	RRMS+ (24) RRMS- (18) SPMS (13) PPMS (25)	36 28 50 53	2.3 2.0 6.5 4.5	EDSS *(RR + SP + PP)*	**0.400**
						EDSS *(RR)*	NR
						EDSS *(SP+PP)*	NR
	*Novakova, 2017*	M	RRMS (59)	37	2.5	EDSS	NR
						MSSS	NR
IgG oligoclonal bands (OCB)	*Sladkova, 2011*	L	CIS (20)	34.9	NR	EDSS	**“positive”**
miR-142-3p	*De Vito, 2021*	M	MS (151)	39.5	2.0	EDSS	NR
						PI	**0.270**
sBCMA + IgG + IgG index	*Milstein, 2019*	L	RRMS (118) PMS (173)	42.3 55.3	NR NR	MSSS *(RR + P: n = 191)*	**0.240**
						MSSS *(RR: n = 71)*	0.190
						MSSS *(P: n = 120)*	**0.260**
						ARMSS *(RR + P: n = 190)*	**0.220**
						ARMSS *(RR: n = 70)*	0.080
						ARMSS *(P: n = 120)*	**0.250**
						MS-DSS *(RR + P: n = 244)*	**0.230**
						MS-DSS *(RR: n = 98)*	0.070
						MS-DSS *(P: n = 146)*	**0.240**
sCD14 + sCD163 + YKL-40	*Milstein, 2019*	L	RRMS (118) PMS (173)	42.3 55.3	NR NR	MSSS *(RR + P: n = 171)*	0.150
						ARMSS *(RR + P: n = 170)*	**0.220**
						MS-DSS *(RR + P: n = 211)*	**0.180**
sCD27	*Milstein, 2019*	L	RRMS (118) PMS (173)	42.3 55.3	NR NR	MSSS *(RR + P: n = 171)*	**0.180**
						MSSS *(RR: n = 63)*	0.200
						MSSS *(P: n = 108)*	0.230
						MS-DSS *(RR + P: n = 210)*	**0.200**
						MS-DSS *(RR: n = 86)*	0.060
						MS-DSS *(P: n = 124)*	**0.240**
TNF-α	*Stampanoni-Bassi, 2018*	L	RRMS (205)	34.8	1.5	EDSS	NR
	*Malekzadeh, 2017*	L	RRMS (23) SPMS (22) PPMS (11)	45.2 (MS)	2.0 5.6 5.0	EDSS *(RR + SP + PP)*	NR
						FSS *(RR + SP + PP)*	NR
	*Obradovic, 2012*	L	MS (60)	43.5	3.8	EDSS	NR
	*Sharief, 1991*	L	PPMS (32)	37.4	3.7	EDSS *(n = 17)*	**0.834**
						PI *(n = 17)*	**0.741**

ARMSS, Age-Related Multiple Sclerosis Severity; BREMS, Bayesian Risk Estimate for Multiple Sclerosis; CIS, Clinically Isolated Syndrome; EDSS, Expanded Disability Status Scale; FSS, Fatigue Severity Scale; H, High quality; L, Low quality; M, Moderate quality; MS, Multiple Sclerosis; MS-DSS, Multiple Sclerosis Disease Severity Scale; MSSS, Multiple Sclerosis Severity Score; NR, not reported; PI, Progression Index; P(MS), Progressive Multiple Sclerosis; PP(MS), Primary Progressive Multiple Sclerosis; RR(MS), Relapsing-remitting Multiple Sclerosis; RR(MS)+, acute exacerbation; RR(MS)-, remission; SP(MS), Secondary Progressive Multiple Sclerosis. Significant p-values are marked in bold.

### 3.2 Inflammatory biomarkers related to changes in clinical status

#### 3.2.1 General overview

In total, 33 papers (AD/MS = 10/23) investigated the relationship between inflammatory biomarkers and disease progression, with the latter based on the longitudinal evolution of validated clinical scores similar to the ones used in the previous section. Statistical methodology mainly relied on correlation analyses and/or regression modelling for both disease entities, but we observed a notable distinction between the AD and MS field for the determination of disease progression. After all, in AD publications, the majority of clinical scores were reported as a change in score over a given follow-up period (e.g., δMMSE defined as the change in MMSE score from baseline to follow-up) with only a few reports of raw scores obtained throughout the study period, whereas for MS the opposite was true with the majority of clinical scores only being cross-sectionally collected at the time of follow-up (e.g., EDSS score at follow-up) and merely a few studies reporting an actual change in scores over time. In case of prediction analyses, a predefined threshold was used when determining the probability of reaching a certain grade of disability with the inflammatory biomarker as a predictor (e.g., the probability to reach an EDSS score of 4.0). Once again, both the MMSE and EDSS were the most frequently used scores for AD (MMSE: 80.0%) and MS (EDSS: 87.0%), respectively. A detailed overview of all the results from the individual papers can be found in the Supplementary Material (**Table S5**).

#### 3.2.2 AD literature

AD literature described 41 distinct inflammatory biomarkers that were investigated for their relationship with disease progression, represented by various clinical scores including the MMSE, DRS-2, ADAS-Cog and Clinical Dementia Rating – Sum of Boxes (CDR-SB), as well as composites of memory and/or executive function (60). Cohorts consisted of patients with MCI, MCI due to AD (based on having an AD biomarker profile and/or clinical AD diagnosis at follow-up; MCI-AD), and dementia due to AD. Sample sizes varied from 32 up to 174 participants and follow-up periods ranged from 9 months to 5 years. Similar to what we observed in cross-sectional analyses, cohorts were mainly representative of the later stages of AD, characterized by lower MMSE scores and higher age. Twenty-two biomarkers were significantly associated with cognitive decline, many of which were also studied in the previous section describing cross-sectional relationships (**Table 3A**). Only MCP-1 (a crucial mediator of the innate immune response that has been implicated in the pathology of several neurodegenerative diseases (61)) showed promising results in more than one study (62, 63).

**Table 3A:** Overview inflammatory biomarkers associated with longitudinal clinical measures in AD publications.

Biomarker	Study	Quality	Cohort (n)	Age (y)	MMSE	Associated clinical outcome measure (follow-up time): statistical analysis	Result statistical analysis
MCP-1 (CCL2)	*Pillai, 2020*	L	MCI-AD (48)	68.1	24.8	δMMSE (9m)*	β = -1.540
						δMMSE (15m)*	β = -2.360
						δCDR-SB (9m)*	**β = 1.230**
						δCDR-SB (15m) *(MCI-AD: n = 40)*	**R = 0.540**
						δCDR-SB (15m)*	**β = 2.820**
			MCI (134)	74.9	26.9	δMMSE (12m)*	**β = -1.450**
						δMMSE (24m)*	β = -1.460
						δMMSE (36m)*	**β = -2.870**
						δCDR-SB (24m)*	β = 0.860
						δCDR-SB (36m)*	**β = 1.430**
						δCDR-SB (36m)	**R = 0.240**
						δCDR-SB (36m) *(MCI-AD: n = 97)*	**R = 0.270**
	*Westin, 2012*	L	MCI-AD (47)	74.0	26.7	Annual decline MMSE (60m)	**R = 0.420**
						Annual decline MMSE (60m)°	**β = 0.390**
CCL4	*Pillai, 2020*	L	MCI (134)	74.9	26.9	δMMSE (36m)*	β = -1.360
						δCDR-SB (36m)*	**β = 0.760**
CCL5	*Pillai, 2020*	L	MCI (134)	74.9	26.9	δMMSE (24m)*	β = -0.430
						δCDR-SB (24m) *(MCI: n = 118)*	R = 0.190
						δCDR-SB (24m)*	**β = 0.410**
YKL-40 (CHI3L1)	*Kester, 2015*	M	MCI (61)	68.0	27.0	MMSE decline: predictor (32m)	β = -0.320
			AD (65)	65.0	22.0	MMSE decline: predictor (46m)	**β = 0.650**
Complement factor C3	*Toledo, 2014*	M	MCI (163)	74.5	27.0	ADAS-Cog: C3xTime (42m)	**β = -0.120**
			AD (83)	74.8	23.7	ADAS-Cog: C3xTime (22m)	β = -0.009
Factor H (FH)	*Toledo 2014*	M	MCI (163)	74.5	27.0	ADAS-Cog: FHxTime (42m)	**β = -0.075**
			AD (83)	74.8	23.7	ADAS-Cog: FHxTime (22m)	β = -0.005
FGF basic	*Taipa, 2019*	M	AD (32)	62.7	18.2	δDRS-2 (12m)	**R = -0.664**
Fibrinogen (FGA)	*Pillai, 2020*	L	MCI-AD (48)	68.1	24.8	δCDR-SB (15m)*	β = -0.270
			MCI (134)	74.9	26.9	δMMSE (12m)*	**β = 0.490**
						δCDR-SB (24m)	R = -0.180
						δCDR-SB (24m)*	**β = -0.230**
Gas-6	*Sainaghi, 2017*	M	AD (50)	65.0	22.0	MMSE decline (24m)	**R = -0.800**
G-CSF	*Taipa, 2019*	M	AD (32)	62.7	18.2	δDRS-2 (12m)	**R = -0.521**
GM-CSF	*Taipa, 2019*	M	AD (32)	62.7	18.2	δDRS-2 (12m)	**R = -0.479**
	*Tarkowski, 2003*	L	MCI (56)	72.0	28.9	MMSE (9m) *(n = 0)*	R = NR
IFN-γ	*Taipa, 2019*	M	AD (32)	62.7	18.2	δDRS-2 (12m)	**R = -0.495**
IL-1β	*Taipa, 2019*	M	AD (32)	62.7	18.2	δDRS-2 (12m)	**R = -0.576**
	*Tarkowski, 2003*	L	MCI (56)	72.0	28.9	MMSE (9m) *(n = 6)*	**R = 0.340**
						δMMSE (9m) *(n = 6)*	**R = 0.300**
IL-4	*Taipa, 2019*	M	AD (32)	62.7	18.2	δDRS-2 (12m)	**R = -0.507**
IL-6	*Taipa, 2019*	M	AD (32)	62.7	18.2	δDRS-2 (12m)	**R = -0.553**
IL-9	*Taipa, 2019*	M	AD (32)	62.7	18.2	δDRS-2 (12m)	**R = -0.578**
IL-17	*Taipa, 2019*	M	AD (32)	62.7	18.2	δDRS-2 (12m)	**R = -0.577**
MIP-1β	*Taipa, 2019*	M	AD (32)	62.7	18.2	δDRS-2 (12m)	**R = -0.577**
MMP3	*Pillai, 2020*	L	MCI-AD (48)	68.1	24.8	δMMSE (9m) *(MCI-AD: n = 40)*	R = 0.380
						δMMSE (9m)*	**β = 2.050**
						δCDR-SB (9m) *(MCI-AD: n = 39)*	R = -0.36
						δCDR-SB (9m)*	**β = -0.970**
						δCDR-SB (15m)*	**β = -0.940**
			MCI (134)	74.9	26.9	δCDR-SB (36m)*	β = -0.530
sTNFR1 score	*Hu, 2021*	H	MCI (174)	75.2	NR	δCDR-SB (60m)	β = -0.026
						δCDR-SB (60m): sTNFR1 score x Months	**β = -0.020**
						Time to CDR > 4.0: High AD + High sTNFR1 scores	**Longer than Low sTNFR1**
						Risk for CDR > 4.0: High AD + High sTNFR1 scores	**HR = 0.454**
						δADNI-Mem-EF (60m)	β = -0.010
						δADNI-Mem-EF (60m): sTNFR1 score x Months	**β = 0.005**
sTREM2	*Pillai, 2021*	L	MCI (67)	74.1	26.8	CDR-SB decline (60m): sTREM2 x Visit number	β = 0.016
			AD (42)	74.2	23.5	CDR-SB decline (60m): sTREM2 x Visit number	**β = -1.334**
sTREM2 score	*Hu, 2021*	H	AD (97)	75.1	NR	δCDR-SB (36m)	β = 0.004
						δCDR-SB (36m): sTREM2 score x Months	**β = -0.040**
						Time to CDR-SB > 7.8 (36m): High AD + High sTREM2 score	**Longer than Low sTREM2**
						Time to CDR-SB > 7.8 (36m): High p-Tau + High sTREM2 score	**Longer than Low sTREM2**
						Risk to CDR-SB > 7.8 (36m): High AD + High sTREM2 scores	**HR = 0.412**
						δADNI-Mem-EF (36m)	β = -0.027
						δADNI-Mem-EF (36m): sTREM2 score x Months	**β = 0.006**

β, regression coefficient; AD, Alzheimer’s disease; ADAS-Cog, Alzheimer’s Disease Assessment Scale – Cognitive Subscale; ADNI-Mem-EF, Average of composite Alzheimer’s Disease Neuroimaging Initiative Memory and Executive Functioning Scores; CDR-SB, Clinical Dementia Rating – Sum of Boxes; DRS-2, Dementia Rating Scale-2; H, High quality; HR, Hazard ratio; L, Low quality; m, months; M, Moderate quality; MCI, Mild Cognitive Impairment; MCI-AD, MCI with AD biomarkers and/or AD diagnosis at follow-up; MMSE, Mini-Mental State Examination; n, number; R, Spearman or Pearson correlation coefficient. Significant p-values are marked in bold. *Multivariable analyses; °Multivariate analyses.

#### 3.2.3 MS literature

MS papers presented longitudinal associations for 29 inflammatory biomarkers with functional disability measured by clinical scoring instruments such as the EDSS, MSSS, PI and the Multiple Sclerosis Functional Composite Scale (MSFC), but also with cognitive performance, based on the Brief Repeatable Battery of Neuropsychological Tests (BRB) or Symbol Digit Modalities Test (SDMT) (64–66). Cohorts were once again mainly representing MS in general and included individuals with CIS, CIS that converted to clinically definite MS during the study, RR and/or progressive MS, with a sample size ranging from 15 to 6,398 participants and a follow-up period between 1 and 10 years. The majority of biomarkers (*n* = 20) showed significant results, though most were evaluated in single studies. OCB, YKL-40 (a glycoprotein secreted by glial cells (67)) and IL-1β (a cytokine involved in the regulation of the innate immune response via microglia and astrocytes (68)) were significantly associated with longitudinal measures of clinical status based on multiple publications and may reflect underlying pathways driving disease progression (**Table 3B**).

**Table 3B:** Overview inflammatory biomarkers associated with longitudinal clinical measures in MS publications.

Biomarker	Study	Quality	Cohort (n)	Age (y)	EDSS	Associated clinical outcome measure (follow-up time): statistical analysis	Result statistical analysis
κ-FLC	*Voortman, 2017*	L	CIS+RRMS (61)	28.8	2.0	δEDSS (57.6m)	R = NR
	*Makshakov, 2015*	L	Converters (98)	32.0	NR	EDSS (24m)	**R = 0.418**
κ-FLC / Λ -FLC ratio	*Rathbone, 2018*	L	CIS (43) RRMS (50)	45.0 40.5	NR NR	EDSS (60m) *(n = 29)*	**R = -0.370**
	*Voortman, 2017*	L	CIS+RRMS (61)	28.8	2.0	δEDSS (57.6my)	R = NR
Adiponectin (Adp)	*Signoriello, 2021*	M	MS (66)	43.9	2.0	EDSS (55.2m): Adp > 9.91 μg/ml	**Higher than Adp < 9.91**
						EDSS increase (55.2m): Adp > 9.91 μg/ml °	OR = 0.62
						MSSS (55.2m): Adp > 9.91 μg/ml	**Higher than Adp < 9.91**
						MSSS increase (55.2m): Adp > 9.91 μg/ml °	OR = 1.72*
						PI (55.2m): Adp > 9.91 μg/ml	**Higher than Adp < 9.91**
AF	*Decker, 2016*	M	MS (30)	31.2	NR	Annual δEDSS (>12m)	**R = 0.810**
BDNF	*Sarchielli, 2002*	L	SPMS (15)	38.4	3.7	EDSS increase (24m) *(n = 8)*	**Lower BDNF values**
CCL3	*Puthenparempil, 2020*	L	RRMS (30)	35.7	NR	Disease reactivation^$^(36.4m): CCL3 > 0.736 pg/mL	**OR = 4.9**
YKL-40 (CHI3L1)	*Gil-Perotin, 2018*	M	RRMS (25)	35.0	2.0	EDSS increase: predictor *	**β = 1.097**
							**HR = 2.996**
	*Comabella, 2010*	L	Converters (48)	26.8	NR	EDSS (12m) *(n = 44)*	**R = 0.340**
						EDSS (24m) *(n = 39)*	**R = 0.400**
						EDSS (36m) *(n = 41)*	**R = 0.470**
						EDSS (48m) (*n = 37)*	**R = 0.380**
						EDSS (60m) *(n = 29)*	R = 0.290
CXCL8 (IL-8)	*Stampanoni-Bassi, 2018*	L	RRMS (150)	NR	NR	EDSS (12m)	R = NR
						EDSS (36m)	**R = 0.242**
						PI (36m)	**R = 0.246**
CXCL12	*Farina, 2017*	M	MS (90)	42.5	1.5	EDSS (120m post onset) *(n = 21)*	**R = 0.679**
GFAP	*Norgren, 2004*	M	RRMS (58) SPMS (21) PPMS (15) PRMS (5)	34 42 44 31	1.5 3.5 3 6	EDSS (48m) *(RR + SP + PP + PR)*	R = 0.210
						PI (48m) *(RR + SP + PP + PR)*	**R = 0.240**
GM-CSF	*Farina, 2017*	M	MS (90)	42.5	1.5	EDSS (120m post onset) *(n = 21)*	**R = 0.626**
IgG oligoclonal bands (OCB)	*Giedraitiene, 2021*	M	RRMS (49)	47.3	2.8	EDSS (60m): OCB^+^	Similar to OCB^-^
						δSDMT(60m) = -3.1 – 1.0x(δRNFL_T) + 3.3xOCB	**R² = 0.599**
						δSDMT(60m) = -8.8 – 1.1x(δRNFL_PMB) + 4.4xOCB	**R² = 0.480**
	*Karrenbauer, 2021*	L	MS OCB^+^ (6494) MS OCB- (828)	38.1 40.8	NR NR	Reach EDSS 3.0 (NR): OCB^+^	Similar to OCB^-^
						Risk EDSS 3.0 (NR): OCB^+^ *(n = 5055)*	**HR = 1.29**
						Reach EDSS 4.0 (NR): OCB^+^	Similar to OCB^-^
						Risk EDSS 4.0 (NR): OCB^+^ *(n = 5802)*	**HR = 1.38**
						Reach EDSS 6.0 (NR): OCB^+^	Similar to OCB^-^
						Risk EDSS 6.0 (NR): OCB^+^ *(n = 6398)*	HR = 1.20
	*Oechtering, 2021*	L	MS+CIS (530)	35.7	NR	MSSS (61.2m): OCB^+^ vs. OCB^-^ *(n = 529)*	**β = 0.860**
						MSSS (61.2m): OCB^+^IgG^-^IgM^-^ *(n = 114)* vs. OCB^-^IgG^-^IgM^-^ *(n = 46)*	**β = 0.940**
						MSSS (61.2m): OCB^+^IgG^-^IgM^-^ vs. OCB^-^IgG^-^IgM^-^ °	**β = 0.730**
						MSSS (61.2m): OCB^+^IgG^+^IgM^-^ *(n = 228)* vs. OCB^-^IgG^-^IgM^-^	**β = 0.730**
						MSSS (61.2m): OCB^+^IgG^+^IgM^-^ vs. OCB^-^IgG^-^IgM^-^ °	**β = 0.860**
						MSSS (61.2m): OCB^+^IgG^+^IgM^+^ *(n = 111)* vs. OCB^-^IgG^-^IgM^-^	**β = 1.030**
						MSSS (61.2m): OCB^+^IgG^+^IgM^+^ vs. OCB^-^IgG^-^IgM^-^ °	**β = 1.110**
	*Farina, 2017*	M	MS (90)	42.5	1.5	EDSS (120m post onset): OCB^+^ *(n = 21)*	**Higher than OCB^-^**
						BRB (120m post onset): OCB^+^ test failed *(n = 21)*	**Higher than OCB^-^**
	*Koch, 2007*	L	MS (143)	39.0	3.0	EDSS increase vs. stable (60m)	Similar OCB^+^ / OCB^-^ ratio
							Similar OCB count
			PPMS (50)	45.5	4.0	EDSS increase vs. stable (60m)	Similar OCB^+^ / OCB^-^ ratio
							Similar OCB count
IgM oligoclonal bands (OCMB)	*Capuano, 2021*	M	RRMS (78)	32.9	2.0	Reach EDSS > 3.0 (115.2m) *(n = 20)*	**HR = 4.39**
						Reach EDSS > 3.0 (115.2m)° *(n = 20)*	**HR = 2.93**
						Reach EDSS > 4.0 (115.2m) *(n = 8)*	**HR = 5.39**
IL-1β	*Farina, 2017*	M	MS (90)	42.5	1.5	EDSS (120m post onset) *(n = 21)*	**R = 0.625**
	*Rossi, 2014*	L	RRMS (170)	36.3	2.2	EDSS (60m): IL-1β^+^	**Higher than IL-1β^-^**
						EDSS increase (60m): %RRMS IL-1β^+^	**Higher than IL-1β^-^**
						Reach EDSS 3.0 (60m): IL-1β^+^	**OR = 4.34**
						Reach EDSS > 3.0 (60m): IL-1β^+^	**OR = 3.38**
						Reach EDSS 4.0 (60m): IL-1β^+^	**OR = 4.12**
						Reach EDSS > 4.0 (60m): IL-1β^+^	**OR = 3.32**
						MSSS (60m): IL-1β^+^	**Higher than IL-1β^-^**
						MSFC worsening (60m): %RRMS IL-1β^+^	**Higher than IL-1β^-^**
						Disability progression based on MSFC (60M): IL-1β^+^	**OR = 2.13**
						MSFC worsening (60M): IL-1β^+^	**OR = 2.21**
						PI increase (60m): IL-1β^+^	**Higher than IL-1β^-^**
						BREMS (60m): IL-1β^+^	**OR = 26.61**
	*Rossi, 2014*	L	RRMS (170)	36.3	2.2	MSSS (48m): IL-1β^+^	**Higher than IL-1β^-^**
						PI (48m): IL-1β^+^	**Higher than IL-1β^-^**
IL-6	*Stampanoni-Bassi, 2018*	L	RRMS (150)	NR	NR	EDSS (12m)	R = NR
						EDSS (24m)	R = 0.194
						EDSS (36m)	**R = 0.274**
						PI (36m)	**R = 0.311**
IL-9	*Ruocco, 2015*	M	RRMS High-IL9 (50) RRMS Low IL9 (57)	32.2 31.8	1.3 2.0	EDSS (48m): IL-9 > 100 pg/ml	**Lower than IL-9 < 100**
						Reach EDSS 4.0 (48m): IL-9 > 100 pg/ml	**OR = 0.24**
						MSSS (48m): IL-9 > 100 pg/ml	**Lower than IL-9 < 100**
						PI (48m): IL-9 > 100 pg/ml	**Lower than IL-9 < 100**
IL-17	*Ruocco, 2015*	M	RRMS Low IL9 (57)	31.8	2.0	Increased PI (48m): IL-17^+^	**Higher than IL17^-^**
miR-142-3p	*Mandolesi, 2017*	L	Active RRMS (18) Nonactive RRMS (12)	32.1 43.2	1.8 1.7	PI (24-60m) *(n = 21)*	**R = 0.500**
Quotient κ-FLC	*Makshakov, 2015*	L	Converters (98)	32.0	NR	EDSS (24m)	**R = 0.410**
TNF-α	*Sharief, 1991*	L	PMS (17)	37.4	3.7	EDSS (24m)	**R = 0.873**
						PI (24m)	**R = 0.851**

β, regression coefficient; BREMS, Bayesian Risk Estimate for Multiple Sclerosis; CIS, Clinically Isolated Syndrome; Converters, CIS patients whom received a MS diagnosis; EDSS, Expanded Disability Status Scale; H, High quality; HR, Hazard ratio; L, Low quality; m, months; M, Moderate quality; MS, Multiple Sclerosis; MSFC, Multiple Sclerosis Functional Composite; MSSS, Multiple Sclerosis Severity Score; NR, Not reported; OR, Odds ratio; PI, Progression Index; P(MS), Progressive Multiple Sclerosis; PP(MS), Primary Progressive Multiple Sclerosis; PR(MS), Progressive Relapsing Multiple Sclerosis; RNFL_PMB, Papillomacular bundle retinal nerve fibre layer thickness; R, Spearman or Pearson correlation coefficient; RNFL_T, Temporal segment retinal nerve fiber layer thickness; RR(MS), Relapsing-Remitting Multiple Sclerosis; SDMT, Symbol Digit Modalities Test; SP(MS), Secondary Progressive Multiple Sclerosis. Significant p-values are marked in bold; *Multivariable analyses; °Multivariate analyses; $ Not specified whether clinical or radiological disease reactivation.

### 3.3 Inflammatory biomarkers related to transition to a more severe disease stage

#### 3.3.1 General overview

Thirty (AD/MS = 8/22) of the 84 distinct publications investigated the clinical relevance of inflammatory biomarkers with regard to transitioning from one (possibly prodromal) disease stage to a more severe disease stage. The progression rate (i.e., the ratio of participants progressing over those remaining stable) varied from 35 to 68% in AD publications and from 28 to 93% in MS reports. Numerous statistical methods were used for the subsequent data analyses, including between-group comparisons, prediction (such as Cox proportional hazard and regression modelling) and discrimination analyses with receiver operating characteristic measures. A comprehensive overview of all statistical analyses and the corresponding results is provided in the Supplementary Material (**Table S6**).

#### 3.3.2 AD literature

Cohorts consisted of MCI subjects, with a sample size varying from 31 to 174 individuals and an observation period ranging from 9 months to 5 years. Sixteen inflammatory biomarkers were investigated for their clinical relevance with regard to progression in the AD continuum; however, MCI cohorts showed characteristics suggestive for late-onset AD, based on a relatively older age and higher MMSE value. Eight biomarkers demonstrated significant relevance for patients that progressed from MCI to dementia due to AD, including MCP-1 (63), neuronal pentraxin 2 (NPTX2), OPN, YKL-40 and several TNF receptors (with related proteins that were also part of a composite score) (**Table 4A**). Only YKL-40 was assessed in two independent studies but conflicting results were reported (69, 70). Both of them showed similar cohort characteristics, but the study with the highest progression rate (i.e., higher number of MCI patients that developed dementia due to AD), what in itself could be due to the longer follow-up period (32.4 versus 24 months), favored YKL-40 as a predictive biomarker (70). Although OPN was only investigated in one study, it was the sole biomarker evaluated at two distinct time points within the same cohort. With this approach, authors could measure the intra-individual change of the CSF levels throughout the disease course. Results demonstrated increased levels in MCI patients that progressed to AD at follow-up, compared with those who remained stable (53).

**Table 4A:** Overview inflammatory biomarkers associated with progression across the AD spectrum.

Biomarker	Study	Quality	Cohort (n)	Age (y)	MMSE	Follow-up cohorts	Associated clinical outcome measure (follow-up time): statistical analysis	Result
MCP-1 (CCL2)	*Westin, 2012*	L	MCI (119)	74.0 64.0	26.7 27.3	MCI-AD (47) sMCI (52)	Time to progression MCI-AD (60m): MCP-1 > 757 pg/ml	**Shorter than MCP-1 < 757 pg/ml**
							Time to progression MCI-AD (60m): MCP-1°	**Shorter**
YKL-40 (CHI3L1)	*Swanson, 2016*	L	MCI (135)	74.6 74.8	26.9 27.0	MCI-AD (47) sMCI (82)	BL CSF levels in MCI-AD (24m)	Similar to sMCI
	*Kester, 2015*	M	MCI (53)	70.0 64.0	26.0 28.0	MCI-AD (36) sMCI (17)	BL CSF levels in MCI-AD (32.4m)	**Higher than sMCI**
							Risk of progression (32.4my)	**HR = 1.003**
NPTX2	*Swanson, 2016*	L	MCI (135)	74.6 74.8	26.9 27.0	MCI-AD (47) sMCI (82)	BL CSF levels in MCI-AD (24m)	**Lower than sMCI**
OPN	*Sun, 2013*	M	MCI (31)	72.0 73.0	27.9 27.1	MCI-AD (13) sMCI (18)	BL CSF levels in MCI-AD (36m)	Similar to sMCI
							BL CSF levels in MCI-AD (36m)	**Lower than FUP CSF levels MCI-AD**
sTNFR1 score	*Hu, 2021*	H	MCI (174)	75.2	NR	MCI-AD (99) sMCI (75)	Risk of progression (60m): High-AD + High sTNFR1 score	**HR = 0.541**
							Time to progression (60m): High-AD + High sTNFR1 score	**Longer than Low sTNFR1 score**
ysTNFR1 score	*Hu, 2021*	H	MCI (174)	75.2	NR	MCI-AD (99) sMCI (75)	Likelihood progression (60m): High-pTau^181^ + High ysTNRF1 score	**Lower than Low ysTNFR1 score**
			MCI (49)	69.3	NR	MCI-AD (18) sMCI (31)	Likelihood progression (43m): High-pTau^181^ + High ysTNRF1 score	**Lower than Low ysTNFR1 score**
TNFR1	*Zhao, 2020*	L	MCI (116)	74.2	26.8	MCI-AD (64) sMCI (52)	Progression free survival time (30.2m): High TNFR1^$^	**Shorter than Low TNFR1**
							Progression free survival time (30.2m): TN^-^, High TNFR1^$^	**Shorter than Low TNFR1**
							Progression free survival time (30.2m): TN^+^, High TNFR1^$^	Similar to Low TNFR1
TNFR2	*Zhao, 2020*	L	MCI (116)	74.2	26.8	MCI-AD (64) sMCI (52)	Progression free survival time (30.2m): High TNFR2^$^	**Longer than Low TNFR2**
							Progression free survival time (30.2m): TN^-^, High TNFR2^$^	**Longer than Low TNFR2**
							Progression free survival time (30.2m): TN^+^, High TNFR2^$^	Similar to Low TNFR2

AD, Alzheimer’s Disease; BL, Baseline; CSF, Cerebrospinal fluid; FUP, Follow-up; H, High quality; HR, Hazard Ratio; L, Low quality; m, months; M, Moderate quality; MCI, Mild Cognitive Impairment; MCI-AD, MCI with AD diagnosis at follow-up; sMCI, stable MCI at follow-up. TN, Tau pathology. Significant p-values are marked in bold; °Multivariate analyses; $ Cut-off values not reported.

#### 3.3.3 MS literature

The vast majority of publications collected from the MS literature investigated the transition from CIS to clinical definite MS, with cohort sizes varying from 18 to 139 participants and a follow-up duration between 10 months and 10 years. The clinical relevance of 62 distinct inflammatory biomarkers was assessed. Half of the studies presented significant results, yet only ten biomarkers were evaluated in multiple studies (**Table 4B**). YKL-40 and CXCL13 (a chemokine implicated in B cell aggregates that develop in the inflamed meninges of PMS patients (71)) showed the most consistent results, with increased CSF levels at baseline in CIS subjects who received a MS diagnosis during follow-up, as compared with those who did not. These studies highlight the importance of both glial cells as well as the innate immune response in the early stages of the disease.

**Table 4B:** Overview inflammatory biomarkers investigated in multiple studies and found to be associated with progression across the MS spectrum.

Biomarker	Study	Quality	Cohort (n)	Age (y)	EDSS	Follow-up cohorts	Associated clinical outcome measure (follow-up time): statistical analysis	Result
κ-FLC	*Voortman, 2017*	L	CIS (48)	NR	NR	Converters (23) Non-converters (25)	BL CSF levels in Converters (57.6m)	Similar to Non-converters
	*Makshakov, 2015*	L	CIS (139)	32.0 33.0	NR NR	Converters (98) Non-converters (41)	BL CSF levels in Converters (24m)	**Higher than Non-converters**
κ-FLC index	*Vecchio, 2020*	M	RIS+CIS (18)	36.3	NR	Converters (6) Non-converters (12)	BL CSF levels in Converters (43.2m)	**Higher than Non-converters**
							Risk of conversion (43.2m)	HR = 1.070
	*Gaetani, 2020*	L	CIS (23)	41.8	1.7	Converters (12) Non-converters (7)	Time to conversion (39.1m): κ-FLC index > 10.6 *(n = 19)*	**C-statistic = 0.630**
κ-FLC / Λ-FLC ratio	*Rathbone, 2018*	L	CIS (43)	45.0	NR	Converters (NR) Non-converters (NR)	BL CSF levels in Converters (60m) *(n = 29)*	Similar to Non-converters
	*Voortman, 2017*	L	CIS (48)	NR	NR	Converters (23) Non-converters (25)	BL CSF levels in Converters (57.6m)	**Lower than Non-converters**
							Risk of conversion 57.6m): κ / Λ ratio ≤ 3.38	**HR = 2.890**
							Likelihood of conversion (57.6m): κ / Λ ratio ≤ 3.38	**OR = 4.860**
Λ-FLC	*Voortman, 2017*	L	CIS (48)	NR	NR	Converters (23) Non-converters (25)	BL CSF levels in Converters (57.6m)	Similar to Non-converters
	*Makshakov, 2015*	L	CIS (139)	32.0 33.0	NR NR	Converters (98) Non-converters (41)	BL CSF levels in Converters (24m)	**Higher than Non-converters**
YKL-40 (CHI3L1)	*De Fino, 2019*	L	CIS (25)	37.4	1.3	Converters (10) Non-converters (14)	BL CSF levels in Converters (24m) *(n = 24)*	**Higher than Non-converters**
							ROC (24m): Converters vs. Non-converters *(n = 24)*	**AUC = 0.790**
	*Gil-Perotin, 2019*	M	RRMS (99)	35.0	2.0	Converters (14) Non-converters (85)	Risk of conversion (52.8m): SPMS °	**HR = 18.040**
	*Thouvenot, 2019*	L	RIS (71)	38.0	NR	Converters (20) Non-converters (51)	BL CSF levels in Converters (16m)	Similar to Non-converters
							Risk of conversion (16m): YKL-40 > 154.6 mg/ml	HR = 2.130
							Risk of conversion (16m): YKL-40 > 154.6 mg/ml °	HR = 1.560
	*Borras, 2016*	L	CIS (50)	33.0 38.0	NR NR	Converters (25) Non-converters (25)	BL CSF levels in Converters (49.2m)	**Higher than Non-converters**
	*Comabella, 2010*	L	CIS (84)	26.8 28.7	NR NR	Converters (48) Non-converters (36)	BL CSF levels in Converters (66m)	**Higher than Non-converters**
							Time to conversion (66m): YKL-40 > 287.9 mg/ml	**Shorter than YKL-40 < 287.9 mg/ml**
							Risk of conversion (66m): YKL-40 > 287.9 mg/ml	**HR = 2.500**
			CIS (52)	33.6 36.5	NR NR	Converters (26) Non-converters (26)	BL CSF levels in Converters (58.8m)	**Higher than Non-converters**
CXCL13	*Olesen, 2019*	L	ON (40)	41.5 39.3	NR NR	Converters (16) Non-converters (24)	BL CSF levels in Converters (28.2m)	**Higher than Non-converters**
							Likelihood of conversion (28.2m): CXCL13 > 37 pg/ml	**OR = 1.050**
	*Ferraro, 2015*	L	CIS (110)	35.0	2.0	Converters (94) Non-converters (16)	BL CSF levels in Converters (40m)	**Higher than Non-converters**
							Risk of conversion (40m): CXCL13 ≥ 15.4 pg/ml	**HR = 2.900**
							Risk of conversion (40m): CXCL13 ≥ 15.4 pg/ml °	**HR = NR**
	*Brettschneider, 2010*	L	CIS (91)	34.0	2.0	Converters (45) Non-converters (46)	BL CSF levels in Converters (24m)	**Higher than Non-converters**
IgG index	*Olesen, 2019*	L	ON (40)	41.5 39.3	NR NR	Converters (16) Non-converters (24)	BL CSF levels in Converters (28.2m)	**Higher than Non-converters**
							Likelihood of conversion (28.2m): IgG index > 0.64	**OR = 11.090**
	*Cinar, 2018*	L	CIS (41)	NR	NR	Converters (35) Non-converters (6)	Risk of conversion (12.8m): IgG index > 0.7	HR = 0.990
							Time to conversion (12.8m): IgG index > 0.7	Longer than IgG index < 0.7
IgG oligoclonal bands (OCB)	*Gaetani, 2020*	L	CIS (19)	41.8	1.7	Converters (12) Non-converters (7)	Time to conversion (39.1m): OCB^+^	Similar to OCB^-^
	*Kolcava, 2020*	L	CIS (64)	36.5	2.0	Converters (45) Non-converters (19)	BL CSF levels in Converters (27m)	**Higher than Non-converters**
							Risk of conversion (27m): OCB^+^	HR = 2.898
							Risk of conversion (27m): OCB^+^ °	HR = 2.348
							Likelihood of conversion (27m): OCB^+^	**OR = 8.306**
							Likelihood of conversion (27m): OCB^+^ °	**OR = 26.599**
	*Olesen, 2019*	L	ON (40)	41.5 39.3	NR NR	Converters (16) Non-converters (24)	BL CSF levels in Converters (28.2m):	**Higher than Non-converters**
							Likelihood of conversion (28.2m): OCB^+^	**OR = 13.000**
	*Cinar, 2018*	L	CIS (41)	NR	NR	Converters (35) Non-converters (6)	Time to conversion (12.8m): OCB^+^ *(n = 26)*	**Shorter than OCB^-^**
							Risk of conversion (12.8m): OCB^+^ *(n = 26)*	HR = 2.160
	*Farina, 2017*	M	RRMS (90)	42.5	1.5	Converters (19) Non-converters (71)	BL CSF levels in Converters (120m)	**Higher than Non-converters**
							Time to conversion (120m): OCB^+^	**Shorter than OCB^-^**
	*Brettschneider, 2010*	L	CIS (91)	34.0	2.0	Converters (45) Non-converters (46)	Truth table (24m): Converters vs. Non-converters	Sensitivity = 0.91 Specificity = 0.36 PPV = 0.59 NPV = 0.81
	*Koch, 2007*	L	RRMS (78)	34.0	2.0	Converters (19) Non-converters (59)	BL CSF levels in Converters (60M)	Similar to Non-converters
	*Tumani, 1998*	L	ON (36)	32.3	NR	Converters (18) Non-converters (18)	Truth table (48m): Converters vs. Non-converters	Sensitivity = 0.83 Specificity = 0.33 PPV = 0.56 NPV = 0.67
OPN	*De Fino, 2019*	L	CIS (24)	37.4	1.3	Converters (10) Non-converters (14)	BL CSF levels in Converters (24m)	Similar to Non-converters
							ROC (24m): Converters vs. Non-converters	**AUC = 0.72**
	*Borras, 2016*	L	CIS (50)	33.0 38.0	NR NR	Converters (25) Non-converters (25)	BL CSF levels in Converters (49.2m)	Similar to Non-converters
Positive CSF^$^	*Thouvenot, 2019*	L	RIS (71)	38.0	NR	Converters (20) Non-converters (51)	BL CSF levels in Converters (16m)	**Higher than Non-converters**
							Risk of conversion (16m) *(n = 70)*	HR = 2.900
							Risk of conversion (16m) ° *(n = 65)*	HR = 1.220
	*Cinar, 2018*	L	CIS (41)	NR	NR	Converters (35) Non-converters (6)	BL CSF levels in Converters (12.8m) *(n = 26)*	**Higher than in Non-converters**

AUC, Area Under the Curve; BL, Baseline; CIS, Clinically Isolated Syndrome; Converters, ON/CIS/RIS subjects whom received MS diagnosis or RRMS subjects whom received SPMS diagnosis; CSF, Cerebrospinal fluid; H, High quality; HR, Hazard Ratio; L, Low quality; M, Moderate quality; MS, Multiple Sclerosis; NPV, Negative Predictive Value; Non-converters, stable ON/CIS/RIS/RRMS subjects; NR, Not reported; ON, Acute Optic Neuritis; OR, Odds Ratio; PPV, Positive Predictive Value; RIS, Radiologically Isolated Syndrome; ROC, Receiver Operator Characteristics; RRMS, Relapsing Remitting Multiple Sclerosis. Significant p-values are marked in bold. °Multivariate analyses; $ Defined as presence of OCB and/or IgG index > 0.7.

## 4 Discussion

With this review, we provide an overview of the inflammatory CSF biomarkers that are related to disease progression in AD and/or MS. YKL-40 was the only biomarker showing relevance for both disorders. In addition, alterations in MCP-1 and OPN were associated with clinical deterioration in patients with AD exclusively, while the same was true for IL-1β, TNF-α, CXCL13, GFAP and OCB in subjects affected by MS. These combined results point towards a prominent role for innate immune response in the CNS, in which microglia are considered to be key mediators (72), in the progression of both disorders. However, we must acknowledge that the studies retrieved by our search were highly heterogenous in study design and methodology, largely complicating the interpretation of their findings. The ‘Standard for Reporting of Diagnostic Accuracy’ initiative states: “To comprehend the results of diagnostic accuracy studies, readers must understand the study design, conduct, analysis, and results of such studies. That goal can be achieved only through complete transparency from authors” (73). This statement nicely summarizes the main difficulties encountered when reviewing the literature. In the following paragraphs, we sum up the most important findings for both disorders, as well as some recommendations that, in our vision, may contribute to an enhanced clinical relevance and improved quality of result reporting when incorporated in future biomarker studies in this field (**Table 5**).

**Table 5:** Recommendations for future research, based on the 'Standard for Reporting of Diagnostic Accuracy’ initiative for diagnostic accuracy tests.

Topic	Items	Relevance
**AD Cohort**	MCI characterization	MCI is a complex disease entity characterized by different subtypes and not only indicative for AD
	AD biomarker confirmation	Verification of AD pathology based on well-established cut-offs for Aβ, T-tau and P-Tau.
**MS Cohort**	MS subtypes separated	Due to important differences between CIS, RR, SP and PP MS with regard to inflammation
	Relapsing versus Remitting Active versus Non-active	Due to important differences between relapse/remission and active/non-active with regard to inflammation
	Influence of DMT	Due to the altering effect that may affect the interpretation of inflammatory biomarkers
**Neurochemical analyses**	Transparent reporting quality measures	To ensure reliability of results based on proper analyses with the selected assay
**Statistical analyses**	Clinical versus statistical significance	Ensure clinical relevance of the results or translate the results to clinical context
**Longitudinal studies**	Appropriate follow-up duration	Including repeated clinical evaluation and diagnostic confirmation of the disease

Aβ, Amyloid-beta; AD, Alzheimer’s disease; CIS, Clinically Isolated Syndrome; DMT, Disease-modifying treatment; MCI, Mild Cognitive Impairment; MS, Multiple Sclerosis; RR(MS), Relapsing Remitting Multiple Sclerosis; SP(MS), Secondary Progressive Multiple Sclerosis; PP(MS), Primary Progressive Multiple Sclerosis; P-tau, Phospho-tau; T-tau, Total-tau.

Is neuroinflammation beneficial or detrimental in AD? To answer this question, we need to take a closer look at the complex biology and temporal behavior of microglia in this disorder. These residential CNS macrophages are responsible for maintaining local homeostasis and can elicit an inflammatory response against potentially harmful agents, both endo- and exogenous. Microglia are highly dynamic cells that were historically described as being activated into a pro- (M1) or anti-inflammatory phenotype (M2). In fact, these cells exists in various states depending on the specific context (age, spatial location, environment) and are active in both health and disease. In line with this, neuroinflammation cannot be generalized as being either detrimental or beneficial, but will, also depending on the context, exert adaptive or maladaptive effects (20, 74). A recent PET imaging study described increased TSPO expression in MCI and dementia due to AD patients, as compared with healthy controls. However, at follow-up, a decreased expression in MCI and increased expression in dementia due to AD was observed. These results prompted the two-peak hypothesis, stating that across the AD spectrum microglia may undergo two major morphological changes, related with changes in microglia function (75). This hypothesis may explain the distinction in microglia states between MCI and dementia due to AD observed at follow-up, despite both of them showing increased TSPO expression when compared with healthy controls. However, increased TSPO expression in the diseased brain is considered a neuroinflammatory marker, not a microglia specific marker (19). Furthermore, equating microglia morphology with function is considered wrong, as different morphologies can execute similar functions (20). We found promising results for YKL-40, MCP-1 and OPN in reflecting disease progression, particularly at the time of transition from the MCI to the dementia stage in the AD continuum (53, 63, 70). These proteins are mainly produced by microglia (51, 76, 77), and have shown increased expression near amyloid plaques or in the cytoplasm of AD brains (78, 79). MCP-1 (which is the most potent activator of the CCR2 receptor) and OPN have been implicated in several neurodegenerative diseases through their respective role as chemoattractant and regulator of microglia and DAMs (77, 80). *In vivo* studies using AD mouse models suggest that the MCP-1/CCR2 axis has varying effects on AD pathogenesis across the stage of the disease. In the early stages, it may enhance accumulation of microglia and clearance of Aβ, whereas in the late stage, it may cause exaggerated inflammatory responses and the development of insoluble Aβ within microglia (81). Perivascular macrophages (PVMs) are the main source of increased OPN in AD mouse models and postmortem AD brains, and seem to regulate phagocytic activity of microglia. Moreover, the notion that the timing of microglial phagocytosis across the disease stages may be crucial to determine whether the outcome will be beneficial or harmful has been lifted forward recently (82). As the CNS immune response seemingly changes depending on the stage of the disease, this may in part explain the inconsistency we found in our results as biomarker findings would then highly depend on the selected cohort and microglia state at the time of sampling. Moreover, worsening of AD pathology may not necessarily follow a linear course (83), further complicating the interpretation of biomarker results. Nonetheless, together these observations point towards a complex and dynamic role of the innate immune response and microglia across the AD spectrum and motivate a continued effort in studying the earliest stages of AD.

In MS, there seems to be a notable distinction between the types of inflammation predominantly underlying the relapsing versus progressive phase of the disease. Whereas the former is mostly driven by recurrent invasion of peripheral immune cells into the CNS with formation of classic active demyelinating lesions, the latter has been associated with seemingly more subtle yet chronically perseverating and compartmentalized immune-related phenomena, such as (a) meningeal collections of lymphoid cells, some of which are organized into follicle-like structures, with underlying subpial cortical demyelination and atrophy, (b) smoldering lesions characterized by a border zone of iron-rich MIMS and (c) widespread microglial inflammation and astrogliosis throughout the NAWM, sometimes also referred to as ‘dirty white matter’ (84, 85). Smoldering lesions can be visualized *in vivo* by susceptibility-weighted MRI as paramagnetic rim lesions and were found to be more abundant in patients with progressive MS (86), correlating with concurrent and forthcoming clinical disability and brain volume loss (87–90). In addition, such lesions have been linked to (presumably intracellular) sodium accumulation and neurofilament release (91, 92), which are considered as very early pathobiological features of the neurodegenerative cascade in MS (93). Recent data have demonstrated that ongoing inflammation in smoldering lesions is mediated by a microglial-astrocytic axis in which complement component C1q seems to be crucial for MIMS activation (26). In general, T- and B-cells do not seem to be directly causing neuronal damage in MS but interact with local microglia, hereby mediating tissue injury via a complex cascade that is still not fully understood (94, 95). At the histological level, microglia account for approximately 40% of the phagocytic cell population in classic active lesions (96), while slowly expanding chronic lesions demonstrate a preferential accumulation of MIMS at the lesion’s edge (97). In fact, it is hypothesized that “true MS” is best represented by such chronic inflammatory state, since relapses (representative of focal inflammation) do not predict disease outcome in untreated patients (98), and confirmed disability worsening occurring independently from relapse and/or MRI activity has frequently been described in cohorts of RR MS (99–101). Microglia seem to be characterized by a similar expression pattern in active and chronic lesions (95), suggesting a parallel function in both types. Since there are less active lesions during progressive MS, microglia become more prominent compared with peripheral immune cell infiltration (102), as the disease evolves. Beside the association between increased TSPO expression in the NAWM and an enhanced likelihood of experiencing progression independent of relapse activity, TSPO is also associated with higher EDSS and MSSS scores and found to be more common in SP compared with RR MS (30, 103). Interestingly, recent work using this technique showed that TSPO expression was most intense in the periventricular region, suggesting a diffusion of inflammatory CSF-derived factors into surrounding tissues (104), while the thalamic activation pattern appeared to be more sensitive than conventional grey matter atrophy measures in predicting future clinical disability worsening (105). These findings, together with our results, support the hypothesis of microglia driving disease progression across the entire MS spectrum.

Due to the role microglia seem to be playing in disease progression in AD and MS, it is not surprising that they are considered as interesting targets for future treatments. One possible approach could be to change the microglia state in one that clears debris and drives regeneration. However, as already mentioned, microglia are highly versatile depending on the specific context and it is likely that the environment, not the state, is the determining factor in deciding how microglia act in disease (20)Nonetheless, attempts are being made to alter the innate immune response in AD and MS through microglia. Bruton’s tyrosine kinase (BTK) is a non-receptor cytosolic signal transducing enzyme predominantly expressed by B-lymphocytes and myeloid cells. Its downstream signaling pathways are involved in multiple processes, including cellular growth, metabolism, proliferation and differentiation (106). Increased activity patterns have been observed post-mortem in AD brain and focal lesions of patients with progressive MS (107, 108). BTK inhibition has been found to reduce phagocytic activity of microglia in preclinical AD models but did not have a major impact on cytokine release (107). Further research is required to truly determine its potential within the AD continuum. On the other hand, BTK inhibition is a booming business in the MS field with several oral agents currently tested in clinical trials (109). For example, a recent phase IIb study has demonstrated a significantly decreased lesional accumulation on conventional brain MRI after 12 weeks of treatment with tolebrutinib, a selective BTK inhibitor for which evidence of BBB penetration exists in animals and humans, as compared with placebo (110). Multiple phase III trials with this agent are ongoing for the moment in relapsing, non-active SP and PP MS, and their results will be eagerly anticipated by the scientific community. Interestingly, glatiramere acetate and dimethyl fumarate, which are commonly used as DMT for MS, have shown therapeutic promise for AD and other models of neurocognitive decline, at least partially attributed to effects on CNS microglia (111–114).

We were unable to perform a meta-analysis due to the heterogeneous nature of the included studies. The observed diversity in AD and MS study designs is in agreement with other reviews (15, 115, 116) and long known to be questionable for evaluating candidate biomarkers. Cohort discrepancies were one of the major limiting factors for the interpretability and reproducibility of the findings yielded by our search. In AD studies, we observed a high variety in age and disease stage of participants at the time of inclusion. MCI cohorts, for example, were rarely described by subtype (e.g., amnestic versus non-amnestic, single- versus multiple-domain, etc.), by follow-up diagnosis (or even lacking follow-up) or by verification of AD pathology via CSF biomarkers (117). In MS papers, individuals were clearly subdivided based on established diagnostic criteria but eventually often grouped together as “general MS” for analytical purposes. Inflammation is highly fluctuating and/or situational in MS, even in the RR group alone as, for instance, increased levels of GFAP were found in a cohort of MS patients (compared with controls) but once you start to depict the subgroups you may find that the results are due to an increase at relapse in RR MS, as compared with stable or even progressive MS (118). The effects of DMT (119) may also influence the interpretation of CSF inflammatory biomarkers with regards to pathophysiological mechanisms such as relation to disease progression. We generally observed a lack of longitudinal studies with appropriate time of observation and periodic clinical evaluation, as well as diagnostic confirmation after extended follow-up in both AD and MS papers. Moreover, the majority of included studies did not report on the analytical quality, including the intra- and inter-assay coefficient of variance, upper and lower limit of quantification and detectability rate. Similarly, statistical analyses generally lacked reports of model assumptions (e.g., visual inspection of QQ-plots and residuals for linear regression models) and approaches for regression analyses were highly heterogenous. Some biomarkers were based on computations that showed statistically significant relevance concerning disease progression but might be too complex for the routine clinical setting. It will be important to make a distinction between statistical and clinical significance when looking for clinically relevant biomarkers and to translate outcomes into interpretable results for clinical use. The abovementioned aspects contributed to the limited quality we observed in a substantial number of papers based on our quality assessment tool (see **Table S3**). Additionally, there is also an important caveat with regard to our own approach for this systematic review. Disease progression was defined only by validated clinical scores, not by other measures such as imaging parameters (e.g., MRI brain volume changes) which will have influenced the selection of papers. However, we wanted to ensure the findings from this manuscript were relevant for the clinical practice, therefore we only included those measures that were examined in this setting. The EDSS score was confirmed as the most frequently used measure of clinical disability (appearing in 96.6% of the MS papers) but is also known to be a rather insensitive outcome, especially in older patients with a higher grade of disability (e.g., on average merely showing an increase of 1 point over 10 years in the natural history of MS) (120), which may be a reason why most of the biomarkers failed to demonstrate clinical relevance.

## 5 Conclusion

YKL-40 was identified as the only CSF-based biomarker related to clinical disease progression in both AD and MS. MCP-1 and OPN showed similar relevance in patients with AD, while the same accounts for IL-1β, TNF-α, CXCL13, GFAP and OCB in subjects affected by MS. Our findings support the increasingly recognized role of the innate immune system, including the microglia, in the pathology of both disorders. However, the retrieved biomarker studies for AD and MS were generally heterogeneous in nature, challenging the interpretational reliability of their results. We have framed some suggestions to tackle this problem in future research (see **Table 5**), focusing on better cohort characterization (e.g., the use of highly qualitative cohorts with biomarker confirmation for AD studies, particularly for the MCI stage, and clear separation of MS subtypes, as well as a distinct grouping of remitting and relapsing MS patients), transparency in neurochemical quality measures, interpretable statistical analyses and longitudinal studies with repeated clinical assessments and diagnostic confirmation during follow-up.

## Author contributions

JT, SE, MB and MD contributed to conception and design of the study. JT performed the systematic search and organized the database. JT and MB performed the quality assessment and analyses. JT wrote the first draft of the manuscript. MB and MD wrote sections of the manuscript. MB, SE and MD supervised the project. All authors contributed to the article and approved the submitted version.
