# Supplementary material for: Cerebrospinal fluid inflammatory biomarkers for disease progression in Alzheimer’s disease and multiple sclerosis: a systematic review
Source: Front Immunol. 2023 Jul 13;14:1162340. doi: 10.3389/fimmu.2023.1162340 (PMC10374015; doi:10.3389/fimmu.2023.1162340)
Supplement: Supplementary file 1 [file DataSheet_1.docx]

Supplementary Material

**Cerebrospinal fluid inflammatory biomarkers for disease progression in Alzheimer’s disease and multiple sclerosis: a systematic review**

**Joke Temmerman, Sebastiaan Engelborghs, Maria Bjerke, Miguel D’Haeseleer***

*** Correspondence:** Miguel D’haeseleer, MD, PhD: [miguel.dhaeseleer@uzbrussel.be](mailto:miguel.dhaeseleer@uzbrussel.be)

# Supplementary Tables

**File name**: Supplementary Table 1

**File format**: “Table S1 - PRISMA_2020_checklist.**docx**”.

**Title of data**: PRISMA 2020 Checklist

**Description of data**: PRISMA 2020 checklist for systematic reviews

**File name**: Supplementary Table 2

**File format**: “Table S2 - Data_Extraction.**xlsx**”

**Title of data**: Data extraction table

**Description of data**: Data extraction table from all the included studies categorized as a) clinical scores at baseline, b) change of such scores over time and c) transition from one disease stage to a more severe stage.

**File name**: Supplementary Table 3

**File format**: “Table S3 - Questionnaire quality assessment papers.**docx**”

**Title of data**: Quality assessment tool

**Description of data**: Quality assessment tool developed for this systematic review, including assessment for diagnostic criteria, neurochemical analysis, clinical scoring, cohort size, statistics and, in case of longitudinal studies, duration of follow-up.

**File name**: Supplementary Table 4

**File format**: “Table S4 - Clinical scores at baseline.**docx**”

**Title of data**: CSF inflammatory markers and clinical scores at baseline.

**Description of data**: Included studies for AD and MS focusing on the relationship between CSF inflammatory biomarkers and clinical scores obtained at baseline.

**File name**: Supplementary Table 5

**File format**: “Table S5 - Longitudinal clinical scores.**docx**”

**Title of data**: CSF inflammatory markers and longitudinal clinical scores

**Description of data**: Included studies for AD and MS focusing on the relationship between CSF inflammatory biomarkers and the change in clinical scores over time.

**File name**: Supplementary Table 6

**File format**: “Table S6 - Transition from one disease stage.**docx**”

**Title of data**: CSF inflammatory markers and transition from one disease stage to a more severe disease stage

**Description of data**: Included studies for AD and MS focusing on the relationship between CSF inflammatory biomarkers and the transition from one disease stage to a more severe disease stage.
