# Supplementary material for: Cerebrospinal fluid inflammatory biomarkers for disease progression in Alzheimer’s disease and multiple sclerosis: a systematic review
Source: Front Immunol. 2023 Jul 13;14:1162340. doi: 10.3389/fimmu.2023.1162340 (PMC10374015; doi:10.3389/fimmu.2023.1162340)
Supplement: Supplementary file 4 [file Table_3.docx]

**Cerebrospinal fluid inflammatory biomarkers for disease progression in Alzheimer’s disease and multiple sclerosis: a systematic review**

**Joke Temmerman1,2,3, Sebastiaan Engelborghs1,2,3, Maria Bjerke1,2,3,4*, Miguel D’Haeseleer1,3,5***

1. Vrije Universiteit Brussel, Center for Neurosciences (C4N); Laarbeeklaan 103, 1090 Jette, Brussels, Belgium.

2. Universiteit Antwerpen, Department of Biomedical Sciences and Institute Born-Bunge, Reference Center for Biological Markers of Dementia (BIODEM); Universiteitsplein 1, 2610 Wilrijk, Antwerp, Belgium.

3. Universitair Ziekenhuis Brussel, Department of Neurology; Laarbeeklaan 101, 1090 Jette, Brussels, Belgium.

4. Universitair Ziekenhuis Brussel, Department of Clinical Biology, Laboratory of Clinical Neurochemistry; Laarbeeklaan 101, 1090 Jette, Brussels, Belgium.

5. Nationaal Multiple Sclerose Centrum (NMSC); Vanheylenstraat 16, 1820 Melsbroek, Steenokkerzeel, Belgium.

Corresponding author: [miguel.dhaeseleer@uzbrussel.be](mailto:miguel.dhaeseleer@uzbrussel.be)

| **Quality questions** | **Response options** | **Quality result** |
| --- | --- | --- |
| **1.a What were the diagnosis criteria for AD?** | a) Clinical assessment NINCDS-ADRDA or NIA-AA criteria or no information was provided  b) Clinical assessment + AD-based biomarkers measured  c) Clinical assessment + AD-based biomarkers measured and applied to cohort criteria | 0  1  2 |
| **1.b What were the diagnosis criteria for MS?** | a) Poser or McDonald 2001-2005 criteria or no information was provided  b) McDonald 2010 criteria  c) McDonald 2017 criteria | 0  1  2 |
| **2.**  **Were measurements reported for the whole cohort wherein CSF had been withdrawn?** | a) Random samples were excluded for non-quantifiable/missing results or due to high CV, or nothing was reported  b) Lower detection limit or other value was assigned to out of range samples  c) All samples were quantifiable | 0  1  2 |
| **2.1 Were the coefficients of variations (CV) reported for intra- (or inter-) assay variations?** | a) No or qualitative measures only  b) Yes: the CV was > 15%  c) Yes: the CV was < 15% | 0  1  2 |
| **3.a Was the standard neuropsychological questioning for AD applied?** | a) No or not reported  b) Yes: one clinical score was assessed  c) Yes: full neuropsychological exam was performed | 0  1  2 |
| **3.b**  **Was the EDSS for MS reported?** | a) No or not reported  b) Yes: only the EDSS score  c) Yes: EDSS score and additional scores | 0  1  2 |
| **4. Was a power calculation performed to determine the required number of participants?** | a) No or not reported  b) Yes | 0  1 |
| **4.1 If a power calculation was undertaken, was the number of participants included appropriate?** | a) No or no reported  b) Yes | 0  1 |
| **5. Was the quality of the statistical analyses appropriate?** | a) No: No reporting of statistical methods or measures related to our criteria  b) Yes: reporting of statistical methods or measures related to our criteria  c) Yes: reporting of statistical methods and measures related to our criteria | 0  1  2 |
| **6. Were adjustments of p-values for multiple comparisons considered?** | a) No  b) Yes | 0  1 |
| **7. Was a validation of findings performed in an independent cohort?** | a) No  b) Yes | 0  1 |
| **8. In the case of longitudinal studies, was the follow-up duration at least 1 year?** | a) No  b) Yes | 0  1 |
| **Total score cross sectional studies** | | **/14** |
| **Total score longitudinal studies** | | **/15** |

| **Reference** | **Q1** | **Q2** | | **Q3** | **Q4** | | **Q5** | **Q6** | **Q7** | **Q8** | **Total score** | **Quality** |
| --- | --- | --- | --- | --- | --- | --- | --- | --- | --- | --- | --- | --- |
| Sharief & Hentges 1991 | 0 | 0 | 0 | 2 | 0 | - | 2 | 0 | 0 | 1 | **5/15** | **LOW** |
| Callea et al. 1999 | 0 | 2 | 2 | 1 | 0 | - | 1 | 0 | 0 | NA | **6/14** | **LOW** |
| Cepok et al. 2001 | 0 | 0 | 0 | 2 | 0 | - | 2 | 1 | 0 | 1 | **6/15** | **LOW** |
| Yuceyar et al. 2001 | 0 | 2 | 0 | 2 | 0 | - | 2 | 0 | 0 | NA | **6/14** | **LOW** |
| Silber et al. 2002 | 0 | 0 | 1 | 1 | 0 | - | 2 | 0 | 0 | NA | **4/14** | **LOW** |
| Tarkowski et al. 2003 | 1 | 1 | 0 | 1 | 0 | - | 2 | 0 | 0 | 0 | **5/15** | **LOW** |
| Ehling et al. 2004 | 0 | 0 | 0 | 1 | 0 | - | 1 | 1 | 0 | NA | **3/14** | **LOW** |
| Fainardi et al. 2006 | 0 | 2 | 2 | 1 | 0 | - | 1 | 0 | 0 | NA | **6/14** | **LOW** |
| Galimberti et al. 2006 | 0 | 0 | 0 | 1 | 0 | - | 2 | 0 | 0 | 1 | **4/15** | **LOW** |
| Piccio et al. 2008 | 0 | 2 | 0 | 2 | 0 | - | 1 | 1 | 0 | NA | **6/14** | **LOW** |
| Popp et al. 2009 | 0 | 0 | 0 | 1 | 0 | - | 2 | 0 | 0 | NA | **3/14** | **LOW** |
| Comi et al. 2010 | 2 | 0 | 0 | 1 | 0 | - | 2 | 0 | 0 | NA | **5/14** | **LOW** |
| Correa et al. 2011 | 1 | 0 | 0 | 2 | 0 | - | 1 | 0 | 0 | NA | **4/14** | **LOW** |
| Sladkova et al. 2011 | 1 | 0 | 0 | 1 | 0 | - | 1 | 0 | 0 | NA | **3/14** | **LOW** |
| Obradovic et al. 2012 | 0 | 0 | 0 | 1 | 0 | - | 1 | 0 | 0 | NA | **2/14** | **LOW** |
| Sun et al. 2013 | 0 | 2 | 2 | 1 | 0 | - | 2 | 0 | 0 | 1 | **8/15** | **MODERATE** |
| Toledo et al. 2014 | 1 | 0 | 2 | 2 | 0 | - | 2 | 1 | 0 | 1 | **9/15** | **MODERATE** |
| Aeinehband et al. 2015 | 0 | 0 | 0 | 2 | 0 | - | 2 | 0 | 0 | NA | **4/15** | **LOW** |
| Ferraro et al. 2015 | 1 | 0 | 0 | 1 | 0 | - | 2 | 0 | 0 | 1 | **5/15** | **LOW** |
| Lueg et al. 2015 | 1 | 0 | 0 | 2 | 0 | - | 2 | 1 | 0 | NA | **6/14** | **LOW** |
| Decker et al. 2016 | 1 | 2 | 2 | 1 | 0 | - | 2 | 0 | 0 | 1 | **9/15** | **MODERATE** |
| Hesse et al. 2016 | 2 | 0 | 0 | 1 | 0 | - | 2 | 0 | 0 | NA | **5/14** | **LOW** |
| Lam et al. 2016 | 1 | 0 | 0 | 1 | 0 | - | 2 | 0 | 0 | NA | **4/14** | **LOW** |
| Huss et al. 2020 | 2 | 0 | 2 | 1 | 0 | - | 2 | 0 | 0 | NA | **7/14** | **MODERATE** |
| Johansson et al. 2017 | 1 | 2 | 2 | 1 | 0 | - | 2 | 1 | 0 | NA | **9/14** | **MODERATE** |
| Malekzadeh et al. 2017 | 0 | 0 | 0 | 1 | 0 | - | 1 | 0 | 0 | NA | **2/14** | **LOW** |
| Novakova et al. 2017 | 1 | 1 | 0 | 2 | 0 | - | 2 | 1 | 0 | NA | **7/14** | **MODERATE** |
| Rizzi et al. 2017 | 0 | 0 | 0 | 1 | 1 | 1 | 2 | 0 | 0 | NA | **5/14** | **LOW** |
| Voortman et al. 2017 | 1 | 0 | 0 | 1 | 0 | - | 2 | 0 | 0 | 1 | **5/15** | **LOW** |
| Abdelhak et al. 2018 | 2 | 2 | 2 | 2 | 0 | - | 2 | 0 | 0 | NA | **10/14** | **MODERATE** |
| Bridel et al. 2018 | 0 | 0 | 2 | 1 | 0 | - | 2 | 0 | 1 | NA | **6/14** | **LOW** |
| Kimura et al. 2018 | 2 | 0 | 0 | 2 | 0 | - | 2 | 0 | 0 | NA | **6/14** | **LOW** |
| Pawlitzki et al. 2018 | 1 | 0 | 0 | 1 | 0 | - | 2 | 1 | 0 | NA | **5/14** | **LOW** |
| Stampanoni-Bassi et al. 2018 | 1 | 1 | 0 | 1 | 0 | - | 1 | 1 | 0 | 1 | **6/15** | **LOW** |
| Gil-Perotin et al. 2019 | 2 | 0 | 2 | 1 | 0 | - | 2 | 1 | 0 | 1 | **9/15** | **MODERATE** |
| Milstein et al. 2019 | 1 | 0 | 0 | 2 | 0 | - | 2 | 1 | 0 | NA | **6/14** | **LOW** |
| Taipa et al. 2019 | 2 | 0 | 1 | 2 | 0 | - | 1 | 1 | 0 | 1 | **8/15** | **LOW** |
| Gaetani et al. 2020 | 1 | 2 | 0 | 1 | 0 | - | 1 | 0 | 0 | 1 | **6/15** | **LOW** |
| Magliozzi et al. 2020 | 0 | 0 | 0 | 1 | 0 | - | 2 | 0 | 0 | 1 | **4/15** | **LOW** |
| De Vito et al. 2021 | 1 | 0 | 0 | 2 | 1 | 1 | 2 | 0 | 0 | NA | **7/14** | **MODERATE** |
| Marastoni et al. 2021 | 1 | 0 | 0 | 1 | 0 | - | 1 | 1 | 0 | NA | **4/14** | **LOW** |
| Rui et al. 2021 | 1 | 2 | 0 | 1 | 0 | - | 2 | 0 | 0 | NA | **6/14** | **LOW** |
| Azzolini et al. 2022 | 2 | 2 | 0 | 2 | 0 | - | 2 | 1 | 0 | NA | **9/14** | **MODERATE** |
| Tumani et al. 1998 | 0 | 0 | 0 | 0 | 0 | - | 2 | 0 | 0 | 1 | **3/15** | **LOW** |
| Koch et al. 2007 | 0 | 0 | 0 | 2 | 0 | - | 2 | 0 | 0 | 1 | **5/15** | **LOW** |
| Brettschneider et al. 2010 | 0 | 0 | 0 | 1 | 0 | - | 2 | 0 | 0 | 1 | **4/15** | **LOW** |
| Comabella et al. 2010 | 0 | 0 | 2 | 1 | 0 | - | 2 | 0 | 1 | 1 | **7/15** | **LOW** |
| Ruet et al. 2010 | 1 | 0 | 0 | 0 | 0 | - | 2 | 0 | 0 | 1 | **4/15** | **LOW** |
| Avsar et al. 2012 | 1 | 0 | 0 | 1 | 0 | - | 1 | 0 | 0 | 1 | **4/15** | **LOW** |
| Westin et al. 2012 | 0 | 0 | 2 | 1 | 0 | - | 2 | 1 | 0 | 1 | **7/15** | **LOW** |
| Rossi et al. 2015 | 1 | 0 | 0 | 1 | 0 | - | 2 | 0 | 0 | 0 | **4/15** | **LOW** |
| Makshakov et al. 2015 | 0 | 0 | 0 | 1 | 0 | - | 2 | 0 | 0 | 1 | **4/15** | **LOW** |
| Kester et al. 2015 | 1 | 0 | 2 | 1 | 0 | - | 2 | 1 | 0 | 1 | **8/15** | **MODERATE** |
| Borras et al. 2016 | 0 | 1 | 0 | 0 | 0 | - | 2 | 0 | 1 | 1 | **5/15** | **LOW** |
| Swanson et al. 2016 | 1 | 0 | 0 | 2 | 0 | - | 2 | 0 | 0 | 1 | **6/15** | **LOW** |
| Farina et al. 2017 | 1 | 0 | 2 | 2 | 0 | - | 2 | 1 | 0 | 1 | **9/15** | **MODERATE** |
| Cinar et al. 2018 | 0 | 0 | 0 | 0 | 0 | - | 2 | 0 | 0 | 1 | **3/15** | **LOW** |
| Rathbone et al. 2018 | 1 | 0 | 0 | 1 | 0 | - | 2 | 0 | 0 | 1 | **5/15** | **LOW** |
| De Fino et al. 2019 | 1 | 0 | 0 | 1 | 0 | - | 2 | 0 | 0 | 1 | **5/15** | **LOW** |
| Olesen et al. 2019 | 1 | 0 | 0 | 0 | 0 | - | 2 | 0 | 0 | 1 | **4/15** | **LOW** |
| Timirci-Kahraman et al. 2019 | 1 | 0 | 0 | 1 | 0 | - | 2 | 0 | 0 | 1 | **5/15** | **LOW** |
| Thouvenot et al. 2019 | 0 | 0 | 0 | 0 | 0 | - | 2 | 1 | 0 | 1 | **4/15** | **LOW** |
| Vecchio et al. 2020 | 2 | 0 | 2 | 0 | 0 | - | 2 | 1 | 0 | 1 | **8/15** | **MODERATE** |
| Zhao et al. 2020 | 1 | 0 | 0 | 2 | 0 | - | 1 | 1 | 0 | 1 | **6/15** | **LOW** |
| Sarchielli et al. 2002 | 0 | 0 | 0 | 1 | 0 | - | 1 | 0 | 0 | 1 | **3/15** | **LOW** |
| Norgren et al. 2004 | 0 | 2 | 0 | 2 | 0 | - | 2 | 0 | 0 | 1 | **7/15** | **MODERATE** |
| Rossi et al. 2014 | 0 | 1 | 0 | 2 | 0 | - | 2 | 0 | 0 | 1 | **6/15** | **LOW** |
| Rossi et al. 2014 | 0 | 1 | 0 | 2 | 0 | - | 2 | 0 | 0 | 1 | **6/15** | **LOW** |
| Ruocco et al. 2015 | 1 | 1 | 0 | 2 | 0 | - | 2 | 0 | 0 | 1 | **7/15** | **LOW** |
| Mandolesi et al. 2017 | 1 | 0 | 0 | 2 | 0 | - | 2 | 0 | 0 | 1 | **6/15** | **LOW** |
| Sainaghi et al. 2017 | 2 | 0 | 2 | 1 | 0 | - | 2 | 0 | 0 | 1 | **8/15** | **MODERATE** |
| Puthenparempil et al. 2020 | 2 | 0 | 0 | 1 | 0 | - | 2 | 0 | 0 | 1 | **6/15** | **LOW** |
| Pillai et al. 2020 | 2 | 0 | 0 | 2 | 0 | - | 2 | 1 | 1 | 0 | **8/15** | **MODERATE** |
| Capuano et al. 2021 | 1 | 2 | 0 | 1 | 0 | - | 2 | 0 | 0 | 1 | **7/15** | **LOW** |
| Pillai et al. 2021 | 2 | 0 | 0 | 1 | 0 | - | 2 | 1 | 1 | 1 | **8/15** | **MODERATE** |
| Kolcava et al. 2020 | 1 | 0 | 0 | 1 | 0 | - | 2 | 1 | 0 | 1 | **6/15** | **LOW** |
| Signoriello et al. 2021 | 2 | 0 | 0 | 2 | 0 | - | 2 | 0 | 0 | 1 | **7/15** | **LOW** |
| Hu et al. 2021 | 0 | 2 | 2 | 2 | 1 | 1 | 2 | 1 | 1 | 1 | **13/15** | **HIGH** |
| Giedraitiene et al. 2021 | 2 | 0 | 0 | 2 | 0 | - | 2 | 0 | 0 | 1 | **7/15** | **LOW** |
| Karrenbauer et al. 2021 | 1 | 0 | 0 | 1 | 0 | - | 1 | 0 | 0 | 0 | **3/15** | **LOW** |
| Oechtering et al. 2021 | 2 | 0 | 0 | 0 | 0 | - | 2 | 0 | 0 | 1 | **5/15** | **LOW** |
