# Supplementary material for: Cerebrospinal fluid inflammatory biomarkers for disease progression in Alzheimer’s disease and multiple sclerosis: a systematic review
Source: Front Immunol. 2023 Jul 13;14:1162340. doi: 10.3389/fimmu.2023.1162340 (PMC10374015; doi:10.3389/fimmu.2023.1162340)
Supplement: Supplementary file 5 [file Table_4.docx]

**Cerebrospinal fluid inflammatory biomarkers for disease progression in Alzheimer’s disease and multiple sclerosis: a systematic review**

**Joke Temmerman^1,2,3^, Sebastiaan Engelborghs^1,2,3^, Maria Bjerke^1,2,3,4^*, Miguel D’Haeseleer^1,3,5^***

1. Vrije Universiteit Brussel, Center for Neurosciences (C4N); Laarbeeklaan 103, 1090 Jette, Brussels, Belgium.

2. Universiteit Antwerpen, Department of Biomedical Sciences and Institute Born-Bunge, Reference Center for Biological Markers of Dementia (BIODEM); Universiteitsplein 1, 2610 Wilrijk, Antwerp, Belgium.

3. Universitair Ziekenhuis Brussel, Department of Neurology; Laarbeeklaan 101, 1090 Jette, Brussels, Belgium.

4. Universitair Ziekenhuis Brussel, Department of Clinical Biology, Laboratory of Clinical Neurochemistry; Laarbeeklaan 101, 1090 Jette, Brussels, Belgium.

5. Nationaal Multiple Sclerose Centrum (NMSC); Vanheylenstraat 16, 1820 Melsbroek, Steenokkerzeel, Belgium.

Corresponding author: [miguel.dhaeseleer@uzbrussel.be](mailto:miguel.dhaeseleer@uzbrussel.be)

CSF inflammatory markers and clinical scores at baseline

| ***MS*** | | | | **Association to inflammatory biomarker assessed with** | | | | | |
| --- | --- | --- | --- | --- | --- | --- | --- | --- | --- |
| **Inflammatory biomarker** | **Reference**  **(first author, year)** | **Cohort** | **Cohort n** | **EDSS** | **MSSS** | **ARMSS** | **MS-DSSS** | **PI** | **Other tests** |
| 8-iso-15(S)-PGF2α/20:4µg | Lam et al. 2016 | RRMS SPMS  PPMS | 23  24  15 | MS  R_S_ = -0.142  *p* = 0.315 |  |  |  |  |  |
| 15(S)-PGF2α/20:4µg | Lam et al. 2016 | RRMS SPMS  PPMS | 23  24  15 | MS  R_S_ = 0.313 ***p* = 0.024** |  |  |  |  |  |
| 15(S)-PGF2α/CSFµg | Lam et al. 2016 | RRMS  SPMS  PPMS | 23  24  15 | MS  R_S_ = 0.280  ***p* = 0.044** |  |  |  |  |  |
| α/β T-cells | Cepok et al. 2001 | RRMS  SPMS  PPMS | 21  6  4 | MS  R_S_ = NR  *p* > 0.05 |  |  |  |  |  |
| Β2-microglobulin | Sladkova et al. 2011 | CIS  RRMS | 20  45 | NR |  |  |  |  |  |
| ɣ/δ T-cells | Cepok et al. 2001 | RRMS  SPMS  PPMS | 21  6  4 | MS  R_S_ = NR  *p* > 0.05 |  |  |  |  |  |
| Κ-FLC | Voortman et al. 2017  Gaetani et al. 2020 | CIS  RRMS  RIS  CIS  RRMS  PMS | 48  13  3  23  34  4 | MS  R_S_ = NR  *p* > 0.05  MS  R_S_ = NR  *p* > 0.05 |  |  |  |  |  |
| Κ-FLC/ Λ-FLC ratio | Voortman et al. 2017 | CIS  RRMS | 48  13 | MS  R_S_ = NR  *p* > 0.05 |  |  |  |  |  |
| Λ-FLC | Voortman et al. 2017  Gaetani et al. 2020 | CIS  RRMS  RIS  CIS  RRMS  PMS | 48  13  3  23  34  4 | MS  R_S_ = NR  *p* > 0.05  MS  R_S_ = NR  *p* > 0.05 |  |  |  |  |  |
| AAT | Sladkova et al. 2011 | CIS  RRMS | 20  45 | NR |  |  |  |  |  |
| AChE | Aeinehband et al. 2015 | RRMS  SPMS  PPMS | 33  9  6 | MS  R² = 0.064  *p* = 0.086 |  |  |  |  |  |
| Agalactosylation factor (AF) = GSL-II/RCA-I binding to IgG | Decker et al. 2016 | CIS  RRMS  SPMS  PPMS | 9  33  8  2 | MS  R_S_ = NR  *p* > 0.05 |  |  |  |  |  |
| Anti-NfL antibodies | Ehling et al. 2004  Silber et al. 2002 | RRMS  SPMS  PPMS  RRMS  SPMS  PPMS | 69  38  23  38  10  18 | MS  R_?_ = NR  *p* > 0.05  MS  R_S_ = 0.62  ***p* = 0.0001** |  |  |  |  |  |
| Anti-NfH antibodies | Silber et al. 2002 | RRMS  SPMS  PPMS | 38  10  18 | MS  R_S_ = 0.51  ***p* = 0.007** |  |  |  |  |  |
| Anti-tubulin antibodies | Silber et al. 2002 | RRMS  SPMS  PPMS | 24  16  9 | MS  R_S_ = 0.34  ***p* = 0.008** |  |  |  |  |  |
| B-cell / monocyte ratio | Cepok et al. 2001 | RRMS  SPMS  PPMS | 21  6  4 | MS  R_S_ = NR  *p* > 0.05 |  |  |  | MS  R_S_ = 0.57  ***p* = 0.0009**  RRMS+SPMS  R_S_ = 0.59  *p* = NR  RRMS  R_S_ = 0.63  *p* = NR |  |
| BAFF | Marastoni et al. 2021 | PPMS | 16 | PPMS  R_S_ = NR  *p* > 0.05 |  |  |  |  |  |
| BuChE | Aeinehband et al. 2015 | RRMS  SPMS  PPMS | 33  9  6 | MS  R² = 0.08  *p* = 0.051 | NR |  |  |  |  |
| MCP-1 (CCL2) | Novakova et al. 2017  Stampanoni-Bassi et al. 2018  Marastoni et al. 2021 | RRMS  RRMS  PPMS | 59  205  16 | RRMS  R_S_ = NR  *p* > 0.05  RRMS  R_Par_ = NR  *p* > 0.10  PPMS  R_S_ = NR  *p* > 0.05 | RRMS  R_S_ = NR  *p* > 0.05 |  |  |  |  |
| CCL8 | Marastoni et al. 2021 | PPMS | 16 | PPMS  R_S_ = NR  *p* > 0.05 |  |  |  |  |  |
| CCL19 | Marastoni et al. 2021 | PPMS | 16 | PPMS  R_S_ = NR  *p* > 0.05 |  |  |  |  |  |
| CCL20 (MIP3A) | Marastoni et al. 2021 | PPMS | 16 | PPMS  R_S_ = NR  *p* > 0.05 |  |  |  |  |  |
| CCL25 | Marastoni et al. 2021 | PPMS | 16 | PPMS  R_S_ = NR  *p* > 0.05 |  |  |  |  |  |
| CD4+ T-cells | Cepok et al. 2001 | RRMS  SPMS  PPMS | 21  6  4 | MS  R_S_ = NR  *p* > 0.05 |  |  |  |  |  |
| CD8+ T-cells | Cepok et al. 2001 | RRMS  SPMS  PPMS | 21  6  4 | MS  R_S_ = NR  *p* > 0.05 |  |  |  |  |  |
| YKL-40 (CHI3L1) | Gil-Perotin et al. 2019  Huss et al. 2020  Novakova et al. 2017 | RRMS  SPMS  PPMS  RRMS  SPMS  PPMS  RRMS | 99  35  23  47  13  26  59 | MS  R_P_ = 0.21  ***p* = 0.009**  RRMS  R_S_ = 0.14  *p* > 0.05  SPMS+PPMS  R_S_ = -0.05  *p* > 0.05  RRMS  R_S_ = 0.274  ***p* = 0.036** | RRMS  R_S_ = 0.297  ***p* = 0.022** |  |  |  |  |
| CHIT | Novakova et al. 2017 | RRMS | 59 | RRMS  R_S_ = NR  *p* > 0.05 | RRMS  R_S_ = NR  *p* > 0.05 |  |  |  |  |
| Complement factor C3 | Aeinehband et al. 2015  Sladkova et al. 2011 | RRMS  SPMS  PPMS  CIS  RRMS | 33  9  6  20  45 | MS  R² = 0.17  ***p* = 0.0034**  NR | MS  R² = 0.13  ***p* = 0.023** |  |  |  |  |
| Complement factor C4 | Sladkova et al. 2011 | CIS  RRMS | 20  45 | NR |  |  |  |  |  |
| CX3CL1 | Marastoni et al. 2021 | PPMS | 16 | PPMS  R_S_ = NR  *p* > 0.05 |  |  |  |  |  |
| CXCL8 (IL-8) | Stampanoni-Bassi et al. 2018  Malekzadeh et al. 2017  Marastoni et al. 2021 | RRMS  RRMS  SPMS  PPMS  PPMS | 205  23  22  11  16 | RRMS  R_Par_ = NR  *p* > 0.10  MS  R_S_ = NR  *p* > 0.05  PPMS  R_S_ = NR  *p* > 0.05 |  |  |  |  | MS: FSS  R_S_ = NR  *p* > 0.05 |
| CXCL10 | Marastoni et al. 2021 | PPMS | 16 | PPMS  R_S_ = NR  *p* > 0.05 |  |  |  |  |  |
| CXCL11 | Marastoni et al. 2021 | PPMS | 16 | PPMS  R_S_ = NR  *p* > 0.05 |  |  |  |  |  |
| CXCL12 | Marastoni et al. 2021 | PPMS | 16 | PPMS  R_S_ = NR  *p* > 0.05 |  |  |  |  |  |
| CXCL13 | Ferraro et al. 2015  Novakova et al. 2017  Lam et al. 2016  Marastoni et al. 2021 | CIS  RRMS  RRMS  SPMS  PPMS  PPMS | 110  59  23  24  15  16 | CIS  R_S_ = NR  *p* > 0.05  RRMS  R_S_ = NR  *p* > 0.05  MS  R_S_ = -0.157  *p* = 0.296  PPMS  R_S_ = NR  *p* > 0.05 | RRMS  R_S_ = NR  *p* > 0.05 |  |  |  |  |
| GFAP | Abdelhak et al. 2018  Novakova et al. 2017  Azzolini et al. 2022 | RRMS  SPMS  PPMS  RRMS  RRMS | 42  13  25  59  51 | MS  R_S_ = 0.4  ***p* < 0.01**  RRMS  R_S_ = NR  *p* > 0.05  SPMS+PPMS  R_S_ = NR  *p* > 0.05  RRMS  R_S_ = NR  *p* > 0.05  RRMS  R_Par_ = NR  *p* = 0.431 | MS  R_S_ = NR  *p* > 0.05  RRMS  R_S_ = NR  *p* > 0.05  SPMS+PPMS  R_S_ = NR  *p* > 0.05  RRMS  R_S_ = NR  *p* > 0.05 | MS:  R_S_ = NR  *p* > 0.05  RRMS:  R_S_ = NR  *p* > 0.05  SPMS+PPMS:  R_S_ = NR  *p* > 0.05 |  |  | RRMS: BREMS  R_Par_ = 0.501  ***p* < 0.001** |
| [GFAP*YKL-40]/NfL | Huss et al. 2020 | RRMS  SPMS  PPMS | 47  13  26 | RRMS  R_S_ = 0.13  *p* > 0.05  SPMS+PPMS  R_S_ = 0.17  *p* > 0.05 |  |  |  |  |  |
| G-CSF | Stampanoni-Bassi et al. 2018 | RRMS | 205 | RRMS  R_Par_ = NR  *p* > 0.10 |  |  |  |  |  |
| GM-CSF | Stampanoni-Bassi et al. 2018  Marastoni et al. 2021 | RRMS  PPMS | 205  16 | RRMS  R_Par_ = NR  *p* > 0.10  PPMS  R_S_ = NR  *p* > 0.05 |  |  |  |  |  |
| Haptoglobin | Sladkova et al. 2011 | CIS  RRMS | 20  45 | NR |  |  |  |  |  |
| IFNα2 | Marastoni et al. 2021 | PPMS | 16 | PPMS  R_S_ = NR  *p* > 0.05 |  |  |  |  |  |
| IFNɣ | Stampanoni-Bassi et al. 2018  Marastoni et al. 2021 | RRMS  PPMS | 205  16 | RRMS  R_Par_ = NR  *p* > 0.10  PPMS  R_S_ = NR  *p* > 0.05 |  |  |  |  |  |
| IFNλ2 | Marastoni et al. 2021 | PPMS | 16 | PPMS  R_S_ = NR  *p* > 0.05 |  |  |  |  |  |
| IgG | Cepok et al. 2001 | RRMS  SPMS  PPMS | 21  6  4 | MS  R_S_ = NR  *p* > 0.05 |  |  |  |  |  |
| IgG oligoclonal bands (OCB) | Sladkova et al. 2011 | CIS  RRMS | 20  45 | CIS  R_S_ = “positive”  ***p* < 0.05** |  |  |  |  |  |
| IgG quotient | Sladkova et al. 2011 | CIS  RRMS | 20  45 | NR |  |  |  |  |  |
| IgA | Cepok et al. 2001 | RRMS  SPMS  PPMS | 21  6  4 | MS  R_S_ = NR  *p* > 0.05 |  |  |  |  |  |
| IgM | Cepok et al. 2001  Magliozzi et al. 2020 | RRMS  SPMS  PPMS  RRMS | 21  6  4  103 | MS  R_S_ = NR  *p* > 0.05  RRMS  R_S_ = -0.04  *p* = 0.678 |  |  |  |  |  |
| IL-1β | Stampanoni-Bassi et al. 2018  Malekzadeh et al. 2017  Marastoni et al. 2021 | RRMS  RRMS  SPMS  PPMS  PPMS | 205  23  22  11  16 | RRMS  R_Par_ = NR  *p* > 0.10  MS  R_S_ = NR  *p* > 0.05  MS  R_S_ = NR  *p* > 0.05 |  |  |  |  | MS: FSS  R_S_ = NR  *p* > 0.05 |
| IL-2 | Stampanoni-Bassi et al. 2018 | RRMS | 205 | RRMS  R_Par_ = NR  *p* > 0.10 |  |  |  |  |  |
| IL-4 | Stampanoni-Bassi et al. 2018  Obradovic et al. 2012  Marastoni et al. 2021 | RRMS  RRMS  SPMS  PPMS | 205  48  12  16 | RRMS  R_Par_ = NR  *p* > 0.10  MS  NR  *p* > 0.05^$^  PPMS  R_S_ = NR  *p* > 0.10 |  |  |  |  |  |
| IL-5 | Stampanoni-Bassi et al. 2018 | RRMS | 205 | RRMS  R_Par_ = NR  *p* > 0.10 |  |  |  |  |  |
| IL-6 | Stampanoni-Bassi et al. 2018  Malekzadeh et al. 2017  Marastoni et al. 2021 | RRMS  RRMS  SPMS  PPMS  PPMS | 205  23  22  11  16 | RRMS  R_Par_ = NR  *p* > 0.10  MS  R_S_ = NR  *p* > 0.05  MS  R_S_ = NR  *p* > 0.05 |  |  |  |  | MS: FSS  R_S_ = NR  *p* > 0.05 |
| IL-7 | Stampanoni-Bassi et al. 2018 | RRMS | 205 | RRMS  R_Par_ = NR  *p* > 0.10 |  |  |  |  |  |
| IL-10 | Stampanoni-Bassi et al. 2018  Marastoni et al. 2021 | RRMS  PPMS | 205  16 | RRMS  R_Par_ = NR  *p* > 0.10  PPMS  R_S_ = NR  *p* > 0.10 |  |  |  |  |  |
| IL-12 | Stampanoni-Bassi et al. 2018 | RRMS | 205 | RRMS  R_Par_ = NR  *p* > 0.10 |  |  |  |  |  |
| IL-12p40 | Marastoni et al. 2021 | PPMS | 16 | PPMS  R_S_ = NR  *p* > 0.05 |  |  |  |  |  |
| IL-13 | Stampanoni-Bassi et al. 2018 | RRMS | 205 | RRMS  R_Par_ = NR  *p* > 0.10 |  |  |  |  |  |
| IL-17 | Stampanoni-Bassi et al. 2018 | RRMS | 205 | RRMS  R_Par_ = NR  *p* > 0.10 |  |  |  |  |  |
| IL-22 | Marastoni et al. 2021 | PPMS | 16 | PPMS  R_S_ = NR  *p* > 0.05 |  |  |  |  |  |
| IL-35 | Marastoni et al. 2021 | PPMS | 16 | PPMS  R_S_ = NR  *p* > 0.05 |  |  |  |  |  |
| Lymphocytes | Cepok et al. 2001 | RRMS  SPMS  PPMS | 21  6  4 | MS  R_S_ = NR  *p* > 0.05 |  |  |  |  |  |
| MIF | Pawlitzki et al. 2018 | MS | 38 | MS  R_S_ = -0.2  *p* = 0.3 |  |  |  |  |  |
| miR-142-3p | De Vito et al. 2021 | CIS/RIS  RRMS  PMS | 18  108  25 | MS  R_S_ = NR  *p* > 0.05 |  |  |  | MS  R_S_ = 0.270  ***p* < 0.001** |  |
| MIP-1β | Stampanoni-Bassi et al. 2018 | RRMS | 205 | RRMS  R_Par_ = NR  *p* > 0.10 |  |  |  |  |  |
| MMP-1 | Marastoni et al. 2021 | PPMS | 16 | PPMS  R_S_ = NR  *p* > 0.05 |  |  |  |  |  |
| MMP-2 | Marastoni et al. 2021 | PPMS | 16 | PPMS  R_S_ = NR  *p* > 0.05 |  |  |  |  |  |
| MMP-9 | Lam et al. 2016 | RRMS  SPMS  PPMS | 23  24  15 | MS  R_S_ = -0.256  *p* = 0.080 |  |  |  |  |  |
| Mononuclear cells | Lam et al. 2016 | RRMS  SPMS  PPMS | 23  24  15 | MS  R_S_ = -0.267  *p* = 0.070 |  |  |  |  |  |
| NK cells | Cepok et al. 2001 | RRMS  SPMS  PPMS | 21  6  4 | MS  R_S_ = NR  *p* > 0.05 |  |  |  |  |  |
| NK-like T-cells | Cepok et al. 2001 | RRMS  SPMS  PPMS | 21  6  4 | MS  R_S_ = NR  *p* > 0.05 |  |  |  |  |  |
| NO_2_^-^ | Yuceyar et al. 2001 | RRMS  SPMS | 15  10 | MS  R_S_ = NR  *p* > 0.05 |  |  |  | MS  R_S_ = NR  *p* > 0.05 |  |
| NO_2_^-^ + NO_3_^-^ | Yuceyar et al. 2001 | RRMS  SPMS | 15  10 | MS  R_S_ = NR  *p* > 0.05 |  |  |  | MS  R_S_ = NR  *p* > 0.05 |  |
| NO_3_^-^ | Yuceyar et al. 2001 | RRMS  SPMS | 15  10 | MS  R_S_ = NR  *p* > 0.05 |  |  |  | MS  R_S_ = NR  *p* > 0.05 |  |
| OPN | Lam et al. 2016  Marastoni et al. 2021 | RRMS  SPMS  PPMS  PPMS | 23  24  15  16 | MS  R_S_ = 0.027  *p* = 0.852  PPMS  R_S_ = NR  *p* > 0.05 |  |  |  |  |  |
| Orosomucoid | Sladkova et al. 2011 | CIS  RRMS | 20  45 | NR |  |  |  |  |  |
| PAF | Callea et al. 1999 | RRMS  SPMS | 11  9 | MS  NR  *p* > 0.05 |  |  |  |  |  |
| sBCMA + IgG + IgG index | Milstein et al. 2019 | RRMS  PMS | 118  173 |  | MS (*n* = 191 )  R_S_ = 0.24  ***p* = 0.007**  RRMS (*n* = 71)  R_S_ = 0.19  *p* = 0.285  PMS (*n* = 120)  R_S_ = 0.26  ***p* = 0.029** | MS (*n* = 190)  R_S_ = 0.22  ***p* = 0.012**  RRMS (*n* = 70)  R_S_ = 0.08  *p* = 0.713  PMS (*n* = 120)  R_S_ = 0.25  ***p* = 0.043** | MS (*n* = 244)  R_S_ = 0.23  ***p* = 0.002**  RRMS (*n* = 98)  R_S_ = 0.07  *p* = 0.079  PMS (*n* = 146)  R_S_ = 0.24  ***p* = 0.022** |  |  |
| sCD14 + sCD163 + YKL-40 | Milstein et al. 2019 | RRMS  PMS | 118  173 |  | MS (*n* = 171)  R_S_ = 0.15  *p* = 0.082  RRMS (*n* = 63)  R_S_ = 0.14  *p* = 0.477  PMS (*n* = 108)  R_S_ = 0.15  *p* =0.321 | MS (*n* = 170)  R_S_ = 0.22  ***p* = 0.012**  RRMS (*n* = 62)  R_S_ = 0.22  *p* = 0.469  PMS (*n* = 108)  R_S_ = 0.19  *p* = 0.108 | MS (*n* = 211)  R_S_ = 0.18  ***p* = 0.0018**  RRMS (*n* = 86)  R_S_ = 0.22  *p* = 0.323  PMS (*n* = 125)  R_S_ = 0.12  *p* = 0.469 |  |  |
| sCD163 | Marastoni et al. 2021 | PPMS | 16 | PPMS  R_S_ = NR  *p* > 0.05 |  |  |  |  |  |
| sCD27 | Milstein et al. 2019 | RRMS  PMS | 118  173 |  | MS (*n* = 171)  R_S_ = 0.18  ***p* = 0.044**  RRMS (*n* = 63)  R_S_ = 0.20  *p* = 0.285  PMS (*n* = 108)  R_S_ = 0.23  *p* =0.061 | MS (*n* = 170)  R_S_ = 0.15  *p* = 0.079  RRMS (*n* = 62)  R_S_ = 0.09  *p* = 0.713  PMS (*n* = 108)  R_S_ = 0.20  *p* =0.108 | MS (*n* = 210)  R_S_ = 0.20  ***p* = 0.014**  RRMS (*n* = 86)  R_S_ = 0.06  *p* = 0.564  PMS (*n* = 124)  R_S_ = 0.24  ***p* = 0.022** |  |  |
| sHLA-I | Fainardi et al. 2006 | RRMS  SPMS  PPMS | 69  21  13 | MS  R_S_ = NR  *p* > 0.05 |  |  |  |  |  |
| sHLA-G | Fainardi et al. 2006 | RRMS  SPMS  PPMS | 69  21  13 | MS  R_S_ = NR  *p* > 0.05 |  |  |  |  |  |
| SPARCL-1 | Bridel et al. 2018 | Dutch - RRMS  - SPMS  Swiss - RRMS  - SPMS  Swedish - RRMS  - SPMS | Dutch - 47  - 29  Swiss - 65  - 27  Swedish - 42  - 39 | Dutch  R_P_ = 0.185  *p* = 0.119  Swiss  R_P_ = 0.027  *p* = 0.801  Swedish  R_P_ = 0.100  *p* = 0.409 |  |  |  |  |  |
| sTNFR1 | Marastoni et al. 2021 | PPMS | 16 | PPMS  R_S_ = NR  *p* > 0.05 |  |  |  |  |  |
| sTNFR2 | Marastoni et al. 2021 | PPMS | 16 | PPMS  R_S_ = NR  *p* > 0.05 |  |  |  |  |  |
| sTREM2 | Piccio et al. 2008  Azzolini et al. 2022 | RRMS  PPMS  RRMS | 52  21  51 | MS  R_?_ = NR  *p* > 0.05  RRMS  R_Par_ = NR  *p* = 0.903 | MS  R_?_ = NR  *p* > 0.05 |  |  |  | RRMS : BREMS  R_Par_ = NR  *p* = 0.142 |
| TNF | Marastoni et al. 2021 | PPMS | 16 | PPMS  R_S_ = NR  *p* > 0.05 |  |  |  |  |  |
| TWEAK (TNFSF12) | Marastoni et al. 2021 | PPMS | 16 | PPMS  R_S_ = NR  *p* > 0.05 |  |  |  |  |  |
| APRIL (TNFSF13) | Marastoni et al. 2021 | PPMS | 16 | PPMS  R_S_ = NR  *p* > 0.05 |  |  |  |  |  |
| LIGHT (TNFSF14) | Marastoni et al. 2021 | PPMS | 16 | PPMS  R_S_ = NR  *p* > 0.05 |  |  |  |  |  |
| TNF-α | Stampanoni-Bassi et al. 2018  Malekzadeh et al. 2017  Obradovic et al. 2012  Sharief & Hentges 1991 | RRMS  RRMS  SPMS  PPMS  RRMS  SPMS  PPMS | 205  23  22  11  48  12  17 | RRMS  R_Par_ = NR  *p* > 0.10  MS  R_S_ = NR  *p* > 0.05  MS  NR  *p* > 0.05^$^  PPMS  R_S_ = 0.834  ***p* < 0.001** |  |  |  | PPMS  R_S_ = 0.741  ***p* < 0.001** | MS: FSS  R_S_ = NR  *p* > 0.05 |
| Transferrin | Sladkova et al. 2011 | CIS  RRMS | 20  45 | NR |  |  |  |  |  |

| ***AD*** | | | | **Association to inflammatory biomarker assessed with** | | | | |
| --- | --- | --- | --- | --- | --- | --- | --- | --- |
| **Inflammatory biomarker** | **Reference**  **(first author, year)** | **Cohort** | **Cohort n** | **MMSE** | **CDR** | **ADAS-Cog** | **DRS-2** | **Other tests** |
| Activated CD4+ T-cells | Lueg et al. 2015 | MCI  AD | 19  54 | MCI:  R_P_ = 0.252  *p* > 0.05  AD  R_P_ = -0.214  *p* > 0.05 |  |  |  | MCI: Verbal learning  n = 15  R_P_ = -0.298  *p* > 0.05  MCI: Verbal retrieval  n = 15  R_P_ = -0.362  *p* > 0.05  MCI: Verbal fluency  n = 15  R_P_ = -0.183  *p* > 0.05  MCI: Visuospatial skills  n = 15  R_P_ = -0.176  *p* > 0.05  MCI: Object naming  n = 15  R_P_ = 0.112  *p* > 0.05  MCI: Set shifting  n = 15  R_P_ = -0.139  *p* > 0.05  AD: Verbal learning  n = 46  R_P_ = -0.441  ***p* < 0.05**  AD: Verbal retrieval  n = 46  R_P_ = -0.465  ***p* < 0.05**  AD: Verbal fluency  n = 46  R_P_ = -0.269  *p* > 0.05  AD: Visuospatial skills  n = 46  R_P_ = -0.364  *p* > 0.05  AD: Object naming  n = 46  R_P_ = -0.105  *p* > 0.05  AD: Set shifting  n = 46  R_P_ = 0.054  *p* > 0.05 |
| Activated CD8+ T-cells | Lueg et al. 2015 | MCI  AD | 19  54 | MCI  R_P_ = -0.209  *p* > 0.05  AD  R_P_ = -0.020  *p* > 0.05 |  |  |  | MCI: Verbal learning  n = 15  R_P_ = -0.545  ***p* < 0.05**  MCI: Verbal retrieval  n = 15  R_P_ = -0.298  *p* > 0.05  MCI: Verbal fluency  n = 15  R_P_ = -0.327  *p* > 0.05  MCI: Visuospatial skills  n = 15  R_P_ = - 0.523  ***p* < 0.05**  MCI: Object naming  n = 15  R_P_ = -0.141  *p* > 0.05  MCI: Set shifting  n = 15  R_P_ = -0.207  *p* > 0.05  AD: Verbal learning  n = 46  R_P_ = -0.371  ***p* < 0.05**  AD: Verbal retrieval  n = 46  R_P_ = -0.744  ***p* < 0.05**  AD: Verbal fluency  n = 46  R_P_ = -0.164  *p* > 0.05  AD: Visuospatial skills  n = 46  R_P_ = -0.334  ***p* < 0.05**  AD: Object naming  n = 46  R_P_ = -0.025  *p* > 0.05  AD: Set shifting  n = 46  R_P_ = -0.041  *p* > 0.05 |
| Anti-Aβ auto antibodies | Kimura et al. 2018 | AD | 69 | AD  R_S_ = 0.009  *p* = 0.943 | AD  R_S_ = 0.090  *p* = 0.532 |  |  | AD: FAB  R_S_ = 0.048  *p* = 0.709 |
| MCP-1 (CCL2) | Kimura et al. 2018  Correa et al. 2011  Galimberti et al. 2006 | AD  AD  AD | 69  22  36 | AD  R_S_ = -0.245  ***p* = 0.0497**  AD  R_?_ = NR  *p* > 0.05  AD  R_S_ = 0.35  ***p* = 0.04** | AD  R_S =_ 0.252  *p* = 0.081 |  |  | AD: FAB  R_S_ = -0.306  ***p* = 0.016** |
| Complement factor C3 | Toledo et al. 2014 | MCI  AD | 163  83 | MCI  β = -0.062  *p* = 1.0  AD  β = -0.45  *p* = 0.53 |  | MCI  β = -0.O61  *p* = 1.0  AD  β = -0.0095  *p* = 1.0 |  | MCI: Composite memory  β = -0.050  *p* = 1.0  AD: Composite memory  β = 0.10  *p* = 0.76  MCI: Composite executive function  β = 0.20  *p* = 0.18  AD: Composite executive function  β = -0.061  *p* = 1.0 |
| CXCL8 (IL-8) | Kimura et al. 2018  Correa et al. 2011  Hesse et al. 2016  Galimberti et al. 2006 | AD  AD  AD  AD | 69  22  ?  36 | AD  R_S_ = -0.180  *p* = 0.190  AD  R_?_ = NR  *p* > 0.05  AD  R_S_ = 0.02  *p* = 0.89  NR | AD  R_S_ = 0.183  *p* = 0.246 |  |  | AD: FAB  R_S_ = -0.293  ***p* = 0.035** |
| CXCL10 | Kimura et al. 2018  Correa et al. 2011  Galimberti et al. 2006 | AD  AD  AD | 69  22  36 | AD  R_S_ = -0.183  *p* = 0.143  AD  R_?_ = NR  *p* > 0.05  AD  R_S_ = 0.37  ***p* = 0.03** | AD  R_S_ = 0.189  *p* = 0.188 |  |  | AD: FAB  R_S_ = -0.255  ***p* = 0.043** |
| Eotaxin (CCL11) | Taipa et al. 2019 | AD | 32 |  |  |  | AD  R_?_ = 0.373  *p* > 0.05 |  |
| FGF basic | Taipa et al. 2019 | AD | 32 |  |  |  | AD  R_?_ = 0.051  *p* > 0.05 |  |
| Factor H | Toledo et al. 2014 | MCI  AD | 163  83 | MCI  β = -0.17^£^  *p* = 1.0  AD  β = -0.14^£^  *p* = 0.54 |  | MCI  β = -0.077^£^  *p* = 1.0  AD  β = -0.017^£^  *p* = 1.0 |  | MCI: Composite memory  β = -0.014^£^  *p* = 1.0  AD: Composite memory  β = 0.083^£^  *p* = 0.59  MCI: Composite executive function  β = 0.077^£^  *p* = 0.29  AD: Composite executive function  β = -0.057^£^  *p* = 1.0 |
| G-CSF | Taipa et al. 2019 | AD | 32 |  |  |  | AD  R_?_ = 0.174  *p* > 0.05 |  |
| GM-CSF | Taipa et al. 2019  Tarkowski et al. 2003 | AD  MCI | 32  56 | NR |  |  | AD  R_?_ = - 0.062  *p* > 0.05 |  |
| IFNɣ | Taipa et al. 2019  Popp et al. 2009 | AD  AD | 32  31 | AD  R_S_ = NR  *p* = 0.563 |  |  | AD  R_?_ = - 0.138  *p* > 0.05 |  |
| IL-1β | Taipa et al. 2019  Hesse et al. 2016  Tarkowski et al. 2003  Rizzi & Roriz-Cruz 2017  Rui et al. 2021 | AD  AD  MCI  aMCI  aMCI  AD | 32  NR  56  33  33  33 | AD  R_S_ = -0.33  ***p* = 0.02**  MCI (n = 6)  R_S_ = 0.46  ***p* = 0.0006**  aMCI  R_?_ = 0.3574  ***p* = 0.0446**  AD  R_?_ = 0.4862  ***p* = 0.0041** |  |  | AD  R_?_ = 0.117  *p* > 0.05 | CERAD  R_S_ = 0.299  ***p* = 0.046**  aMCI: MoCA  R_?_ = 0.3734  ***p* = 0.0323**  AD: MoCA  R_?_ = 0.4993  ***p* = 0.0031** |
| IL-1ra | Taipa et al. 2019 | AD | 32 |  |  |  | AD  R_?_ = 0.242  *p* > 0.05 |  |
| IL-2 | Taipa et al. 2019 | AD | 32 |  |  |  | AD  R_?_ = - 0.220  *p* > 0.05 |  |
| IL-4 | Taipa et al. 2019 | AD | 32 |  |  |  | AD  R_?_ = 0.033  *p* > 0.05 |  |
| IL-5 | Taipa et al. 2019 | AD | 32 |  |  |  | AD  R_?_ = 0.211  *p* > 0.05 |  |
| IL-6 | Kimura et al. 2018  Taipa et al. 2019  Popp et al. 2009  Rizzi & Roriz-Cruz 2017 | AD  AD  AD  aMCI | 69  32  31  33 | AD  R_S_ = -0.184  *p* = 0.137  AD  R_?_ = NR  *p* = 0.724 | AD  R_S_ = 0.159  *p* = 0.266 |  | AD  R_?_ = 0.112  *p* > 0.05 | AD: FAB  R_S_ = -0.217  *p* = 0.085  CERAD  NR |
| IL-7 | Taipa et al. 2019 | AD | 32 |  |  |  | AD  R_?_ = 0.415  *p* > 0.05 |  |
| IL-9 | Taipa et al. 2019 | AD | 32 |  |  |  | AD  R_?_ = 0.267  *p* > 0.05 |  |
| IL-10 | Taipa et al. 2019 | AD | 32 |  |  |  | AD  R_?_ = 0.193  *p* > 0.05 |  |
| IL-12 | Taipa et al. 2019 | AD | 32 |  |  |  | AD  R_?_ = 0.10  *p* > 0.05 |  |
| IL-12/23p40 | Johansson et al. 2017 | AD | 30 | AD  R_S_ = -0.23  *p* > 0.05 |  |  |  |  |
| IL-13 | Taipa et al. 2019 | AD | 32 |  |  |  | AD  R_?_ = 0.394  *p* > 0.05 |  |
| IL-17 | Taipa et al. 2019 | AD | 32 |  |  |  | AD  R_?_ = 0.205  *p* > 0.05 |  |
| MIF | Popp et al. 2009 | AD | 31 | AD  R_S_ = NR  *p* = 0.610 |  |  |  |  |
| MIP-1β | Taipa et al. 2019 | AD | 32 |  |  |  | AD  R_?_ = 0.429  ***p* < 0.05** |  |
| OPN | Comi et al. 2010  Sun et al. 2013 | AD  AD | 67  35 | AD  R_S_ = 0.58  ***p* < 0.0001**  AD  R_P_ = 0.53  ***p* < 0.001** |  |  |  |  |
| PDGF-BB | Taipa et al. 2019 | AD | 32 |  |  |  | AD  R_?_ = 0.311  *p* > 0.05 |  |
| TGF-β | Tarkowski et al. 2003 | MCI | 56 | MCI  R_S_ = -0.03  *p* > 0.05 |  |  |  |  |
| TNF-α | Taipa et al. 2019  Hesse et al. 2016  Popp et al. 2009  Tarkowski et al. 2003  Rizzi & Roriz-Cruz 2017 | AD  AD  AD  MCI  aMCI | 32  ?  31  56  33 | AD  R_S_ = -0.03  *p* = 0.86  AD  R_S_ = NR  *p* = 0.429  MCI  R_S_ = 0.04  *p* > 0.05 |  |  | AD  R_?_ = 0.373  *p* > 0.05 | CERAD  NR |
| VEGF | Taipa et al. 2019 | AD | 32 |  |  |  | AD  R_?_ = 0.373  *p* > 0.05 |  |

**Significant results are reported in bold**

***Legend***

R_S_  Spearman correlation

R_P_ Pearson correlation

R_Par_ Partial correlation

R_?_ Spearman or Pearson correlation

^$^ Linear regression curve

^£^ Mixed-effects model

***Abbreviations***

AD Alzheimer’s disease

aMCI Amnestic mild cognitive impairment

CIS Clinically isolated syndrome

MCI Mild cognitive impairment

MS Multiple sclerosis

n Number

NR Not reported

PMS Progressive multiple sclerosis

PPMS Primary progressive multiple sclerosis

RIS Radiologically isolated syndrome

RRMS Relapsing-remitting multiple sclerosis

SPMS Secondary progressive multiple sclerosis

***Clinical scores***

ADAS-Cog Alzheimer's Disease Assessment Scale-Cognitive subscale

ARMSS Age-Related Multiple Sclerosis Severity

BREMS Bayesian Risk Estimate for Multiple Sclerosis

CDR Clinical Dementia Rating

CERAD Consortium to Establish a Registry for Alzheimer's Disease (CERAD) neuropsychological battery

DRS-2 Dementia Rating Scale-2

EDSS Expanded Disability Status Scale

FAB Frontal Assessment Battery

FSS Fatigue Severity Scale

MMSE Mini-Mental State Exam

MoCA Montreal Cognitive Assessment

MS-DSSS Multiple Sclerosis-Disease Severity Scale

MSSS Multiple Sclerosis Severity Score

PI Progression index (EDSS / disease duration)
