# Supplementary material for: Cerebrospinal fluid inflammatory biomarkers for disease progression in Alzheimer’s disease and multiple sclerosis: a systematic review
Source: Front Immunol. 2023 Jul 13;14:1162340. doi: 10.3389/fimmu.2023.1162340 (PMC10374015; doi:10.3389/fimmu.2023.1162340)
Supplement: Supplementary file 6 [file Table_5.docx]

**Cerebrospinal fluid inflammatory biomarkers for disease progression in Alzheimer’s disease and multiple sclerosis: a systematic review**

**Joke Temmerman^1,2,3^, Sebastiaan Engelborghs^1,2,3^, Maria Bjerke^1,2,3,4^*, Miguel D’Haeseleer^1,3,5^***

1. Vrije Universiteit Brussel, Center for Neurosciences (C4N); Laarbeeklaan 103, 1090 Jette, Brussels, Belgium.

2. Universiteit Antwerpen, Department of Biomedical Sciences and Institute Born-Bunge, Reference Center for Biological Markers of Dementia (BIODEM); Universiteitsplein 1, 2610 Wilrijk, Antwerp, Belgium.

3. Universitair Ziekenhuis Brussel, Department of Neurology; Laarbeeklaan 101, 1090 Jette, Brussels, Belgium.

4. Universitair Ziekenhuis Brussel, Department of Clinical Biology, Laboratory of Clinical Neurochemistry; Laarbeeklaan 101, 1090 Jette, Brussels, Belgium.

5. Nationaal Multiple Sclerose Centrum (NMSC); Vanheylenstraat 16, 1820 Melsbroek, Steenokkerzeel, Belgium.

Corresponding author: [miguel.dhaeseleer@uzbrussel.be](mailto:miguel.dhaeseleer@uzbrussel.be)

CSF inflammatory markers and longitudinal clinical scores

| ***MS*** | | | | | **Association to inflammatory biomarker assessed with** | | | | |
| --- | --- | --- | --- | --- | --- | --- | --- | --- | --- |
| **Inflammatory biomarker** | **Reference**  **(first author, year)** | **Cohort** | **Cohort n** | **Timepoint clinical score** | **EDSS** | **MSSS** | **MSFC** | **PI** | **Other** |
| Κ-FLC | Makshakov et al. 2015  Voortman et al. 2017 | MS-converters  CIS + RRMS | 98  61 (48+13) | 2 years  BL  4.8^*^ years | R_S_ = 0.4184  ***p* = 0.0072**  δEDSS  R_S_ = NR  *p* > 0.05 |  |  |  |  |
| Κ-FLC / Λ-FLC | Voortman et al. 2017  Rathbone et al. 2018 | CIS + RRMS  CIS + RRMS | 61 (48+13)  29 (NR + NR) | BL  4.8^*^ years  5 years | δEDSS  R_S_ = NR  *p* > 0.05  R_S_ = -0.37  ***p* = 0.049**  Reach EDSS > 3.0  κ/λ FLC < 9  Sensitivity = 0.75  Specificity = 0.57 |  |  |  |  |
| Λ-FLC | Makshakov et al. 2015  Voortman et al. 2017 | MS-converters  CIS + RRMS | 98  61 (48+13) | 2 years  BL  4.8^*^ years | R_S_ = NR  *p* > 0.05  δEDSS  R_S_ = NR  *p* > 0.05 |  |  |  |  |
| Adiponectin (Adp) | Signoriello et al. 2021 | MS | 66 | 4.6^^^ years | Cut-off 9.91µg/mL  High-Adp vs. Low-Adp  ***p* = 0.03**  High-Adp EDSS increase: multivariate  OR = 0.62  95%CI = 0.27 - 1.39  *p* = 0.25 | Cut-off 9.91µg/mL  High-Adp vs. Low-Adp  ***p* = 0.04**  High-Adp MSSS increase: multivariate  OR = 1.72  95%CI = 1.05 - 2.79  ***p* = 0.02** |  | Cut-off 9.91µg/mL  High-Adp vs. Low-Adp  *p* = 0.40 |  |
| AF = GSL-II/RCA-I binding to IgG | Decker et al. 2016 | MS | 30 | > 1 year | δEDSS per year  R_S_ = 0.81  ***p* = 0.0002**  EDSS increase > 0.25 per year  AF > 1.6  Sensitivity = 0.89  Specificity = 0.60 |  |  |  |  |
| BDNF | Sarchielli et al. 2002 | SPMS | 15 | 2 years | EDSS increase (*n* = 8) vs. EDSS stable (*n* = 7)  *p* = NR |  |  |  |  |
| CCL3 | Puthenparempil et al. 2020 | RRMS | 30 | 36.4^^^ months | CCL3-low: < 0.495 pg/ml (*n* = 9)  CCL3-med: 0.495 – 0.798 pg/ml (*n* = 15)  CCL3-high: > 0.798 pg/ml (*n* = 16)  Low vs. Med vs. High: disease reactivation^$^  OR = 4.9  95%CI = 1.8 – 13.3  ***p* = 0.002** |  |  |  |  |
| YKL-40 (CHI3L1) | Comabella et al. 2010  Gil-Perotin et al. 2018 | MS-converters  RRMS | 48  25 | 1 year (*n* = 44)  2 years (*n* = 39)  3 years (*n* = 41)  4 years (*n* = 37)  5 years (*n* = 29)  4.4^*^ years | R_S_ = 0.34  ***p* = 0.025**  R_S_ = 0.40  ***p* = 0.012**  R_S_ = 0.47  ***p* = 0.002**  R_S_ = 0.38  ***p* = 0.022**  R_S_ = 0.29  *p* = 0.131  1 point EDSS increase predictor: bivariate  **Log rank *p* = 0.018**  1 point EDSS increase predictor: multivariate:  Coefficient = 1.097  SD = 0.438  95%CI = 1.27 – 7.07  ***p* = 0.008** |  |  |  |  |
| CXCL8 (IL-8) | Stampanoni-Bassi et al. 2018 | RRMS | 150 | 1 year  3 years | R_S_ = NR  *p* > 0.2  R_S_ = 0.242  ***p* = 0.016** |  |  | R_S_ = 0.246  ***p* = 0.014** |  |
| CXCL12 | Farina et al. 2017 | MS OCB+ | 21 | 10 years after disease onset | R_S_ = 0.679  ***p* = 0.022** |  |  |  |  |
| GFAP | Norgren et al. 2004 | RRMS + SPMS + PPMS + PRMS | 99 (58+21+15+5) | 4 years | R_Par_ = 0.21  *p* = 0.05 |  |  | R_Par_ = 0.24  ***p* = 0.02** |  |
| GM-CSF | Farina et al. 2017 | MS OCB+ | 21 | 10 years after disease onset | R_S_ = 0.626  ***p* = 0.039** |  |  |  |  |
| IgA | Magliozzi et al. 2020  Oechtering et al. 2021 | RRMS  MS+CIS | 70  530 | 2 years  5.1 years^*^ | R_S_ = NR  *p* > 0.05 | IgA+ vs. IgA- (n = 529) univariate  Coefficient = -0.12  95%CI = -0.71 – 0.47  *p* = 0.697 |  |  |  |
| IgG | Magliozzi et al. 2020  Oechtering et al. 2021 | RRMS  MS+CIS | 70  530 | 2 years  5.1 years^*^ | R_S_ = NR  *p* > 0.05 | IgG+ vs. IgG- (n = 529) univariate  Coefficient = 0.38  95%CI = -0.07 – 0.72  *p* = 0.106 |  |  |  |
| IgG index | Giedraitiene et al. 2021 | RRMS | 49 | 5 years | Elevated IgG index vs. Normal IgG index  *p* > 0.05 |  |  |  |  |
| IgG oligoclonal bands (OCB) | Koch et al. 2007  Farina et al. 2017  Giedraitiene et al. 2021  Karrenbauer et al. 2021  Oechtering et al. 2021 | MS  PPMS  MS OCB+  MS OCB-  RRMS  MS OCB+  MS OCB-  OCB-IgG-IgM-  OCB+IgG-IgM-  OCB+IgG+IgM-  OCB+IgG+IgM+ | 143  50  50  40  49  6494  828  46  114  229  111 | 5 years  10 years after disease onset  5 years  NR  5.1 years^*^ | EDSS increase vs. EDSS stable  Neg/pos: *p* = 0.42  Number: *p* = 0.11  EDSS increase vs. EDSS stable  Neg/pos: *p* = 0.26  Number**: *p* = 0.08**  OCB+ vs. OCB-  ***p* < 0.0001**  OCB+ vs. OCB-  *p* > 0.05  Reach EDSS 3.0: OCB+ vs. OCB-  *p* = 0.055  Risk EDSS 3.0 (OCB+, n = 5055)  HR = 1.29  95%CI = 1.12 – 1.48  ***p* < 0.001**  Reach EDSS 4.0: OCB+ vs. OCB-  *p* = 0.466  Risk EDSS 4.0 (OCB+, n = 5802)  HR = 1.38  95%CI = 1.17 – 1.63  ***p* < 0.001**  Reach EDSS 6.0: OCB+ vs. OCB-  *p* = 0.139  Risk EDSS 6.0 (OCB+, n = 6398)  HR = 1.20  95%CI = 0.98 – 1.41  *p* = 0.08 | OCB+ vs. OCB- (n = 529) univariate  Coefficient = 0.86  95%CI = 0.25 – 1.46  ***p* < 0.01**  OCB+IgG-IgM- vs. OCB-IgG-IgM- univariate  Coefficient = 0.94  95%CI = 0.17 – 1.70  ***p* = 0.016**  OCB+IgG-IgM- vs. OCB-IgG-IgM- multivariate  Coefficient = 0.73  95%CI = 0.01 – 1.45  ***p* = 0.047**  OCB+IgG+IgM- (n = 228) vs. OCB-IgG-IgM- univariate  Coefficient = 0.73  95%CI = 0.03 – 1.44  ***p* = 0.042**  OCB+IgG+IgM- (n = 228) vs. OCB-IgG-IgM- multivariate  Coefficient = 0.86  95%CI = 0.19 – 1.52  ***p* = 0.012**  OCB+IgG+IgM+ vs. OCB-IgG-IgM- univariate  Coefficient = 1.03  95%CI = 0.26 – 1.77  ***p* < 0.01**  OCB+IgG+IgM+ vs. OCB-IgG-IgM- multivariate  Coefficient = 1.11  95%CI = 0.38 – 1.84  ***p* < 0.01** |  |  | OCB+ vs. OCB-: failed BRB  ***p* = 0.013**  Model SDMT  [δSDMT = -3.1 – 1.0*(δRNFL_T) + 3.3*OCB]  R² = 0.599  ***p* < 0.01**  Model SDMT  [δSDMT = -8.8 – 1.1*(δRNFL_PMB) + 4.4*OCB]  R² = 0.480  ***p* < 0.01** |
| IgM | Magliozzi et al. 2020  Oechtering et al. 2021 | RRMS  MS+CIS | 70  530 | BL  2 years  5.1 years^*^ | δEDSS  R_S_ = 0.09  *p* = 0.498 | IgM+ vs. IgM- (n = 529) univariate  Coefficient = 0.07  95%CI = -0.36 – 0.50  *p* = 0.748 |  |  |  |
| IgM index | Capuano et al. 2021 | RRMS | 78 | 9.6^*^ years | IgM-index > 0.10: reach EDSS > 3.0 univariate (*n* = 20)  HR = 1.88  95% CI = 0.60 - 5.97  *p* = 0.280 |  |  |  |  |
| IgM oligoclonal bands (OCMB) | Capuano et al. 2021 | RRMS | 78 | 9.6^*^ years | Reach EDSS > 3.0 univariate (*n* = 20)  HR = 4.39  95% CI = 1.64-11.78  ***p* = 0.003**  Reach EDSS > 3.0 multivariate (*n* = 20)  HR = 2.93  95%CI = 1.05 - 8.12  ***p* = 0.039**  Reach EDSS > 4.0 univariate (*n* = 8)  HR = 5.39  95% CI = 1.07-27.12  ***p* = 0.041** |  |  |  |  |
| IL-1β | Rossi et al. 2014  Rossi et al. 2014  Farina et al. 2017 | RRMS IL1β+  RRMS IL1β-  RRMS IL1β+  RRMS IL1β-  MS OCB+ | 77  93  77  93  21 | 5^*^ years  4 years  10 years after disease onset | IL1β+ vs. IL1β-  ***p* < 0.01**  %RRMS EDSS worsening: IL1β+ vs. IL1β-  ***p* = 0.03**  Reach EDSS 3.0 RRMS IL1β+ vs. IL1β-  Coefficient = 1.47  SE = 0.42  OR = 4.34  95%CI = 1.93 – 9.83  ***p* < 0.001**  Reach EDSS > 3.0 RRMS IL1β+ vs. IL1β-  Coefficient = 1.22  SE = 0.35  OR = 3.38  95%CI = 1.69 – 6.79  ***p* < 0.001**  Reach EDSS 4.0 RRMS IL1β+ vs. IL1β-  Coefficient = 1.41  SE = 0.45  OR = 4.12  95%CI = 1.69 – 10.02  ***p* = 0.001**  Reach EDSS > 4.0 RRMS IL1β+ vs. IL1β-  Coefficient = 1.20  SE = 0.39  OR = 3.32  95%CI = 1.54 – 7.19  ***p* = 0.002**  R_S_ = 0.625  ***p* = 0.040** | IL1β+ vs. IL1β-  ***p* < 0.01**  IL1β+ vs. IL1β-  ***p* < 0.01** | %RRMS MSFC worsening  ***p* = 0.02**  MSFC progression RRMS IL1β+ vs. IL1β-  Coefficient = 0.75  SE = 0.33  OR = 2.13  95%CI = 1.11 – 4.09  ***p* = 0.02**  MSFC worsening RRMS IL1β+ vs. IL1β-  Coefficient = 0.79  SE = 0.35  OR = 2.21  95%CI = 1.10 – 4.41  ***p* = 0.02** | IL1β+ vs. IL1β-  ***p* < 0.01**  IL1β+ vs. IL1β-  ***p* < 0.01** | BREMS RRMS IL1β- vs. IL1β+  Coefficient = 3.28  SE = 1.51  OR = 26.61  95%CI = 1.37 – 515.47  ***p* = 0.03** |
| IL-4 | Ruocco et al. 2015 | RRMS IL4+  RRMS IL4- | 41  66 | 4 years |  |  |  | IL4+ vs. IL4-  *p* > 0.05 |  |
| IL-6 | Stampanoni-Bassi et al. 2018 | RRMS | 150 | 1 year  2 years  3 years | R_S_ = NR  *p* > 0.2  R_S_ = 0.194  *p* = 0.064  R_S_ = 0.274  ***p* = 0.012** |  |  | R_s_ = 0.311  ***p* = 0.004** |  |
| IL-9 | Ruocco et al. 2015 | RRMS High-IL9  RRMS Low-IL9 | 50  57 | 4 years | Cut-off: 100 pg/ml  High-IL9 vs. Low-IL9  ***p* < 0.05**  Reach EDSS 4.0: High-IL9  Coefficient = -1.43  SE = 0.65  OR = 0.24  ***p* = 0.02** | High-IL9 vs. Low-IL9  ***p* < 0.05** |  | High-IL9 vs. Low-IL9  ***p* < 0.05** |  |
| IL-17 | Ruocco et al. 2015 | RRMS Low-IL9 | 57 | 4 years |  |  |  | Low-IL9 + IL17+ vs. Low-IL9 + IL17- : increase PI  ***p* < 0.05** |  |
| Lipid-specific IgM oligoclonal bands (LS-OCMB) | Capuano et al. 2021 | RRMS | 78 | 9.6^*^ years | Reach EDSS > 3.0 univariate (*n* = 20)  HR = 2.03  95% CI: 0.75 - 5.51  *p* = 0.162  Reach EDSS > 4.0 univariate (*n* = 8)  HR = 1.28  95% CI = 0.25 - 6.40  *p* = 0.766 |  |  |  |  |
| miRNA-142-3p | Mandolesi et al. 2017 | RRMS | 21 | 2-5 years |  |  |  | R_S_ = 0.5  ***p* = 0.022** |  |
| Q-κ | Makshakov et al. 2015 | MS-converters | 98 | 2 years | R_S_ = 0.4101  ***p* = 0.0117** |  |  |  |  |
| Q-λ | Makshakov et al. 2015 | MS-converters | 98 | 2 years | R_S_ = NR  *p* > 0.05 |  |  |  |  |
| TNF-α | Sharief & Hentges 1991 | Chronic PMS | 17 | 24 months | R_S_ = 0.873  ***p* < 0.001** |  |  | R_S_ = 0.851  ***p* < 0.001** |  |

| ***AD*** | | | | | **Association to inflammatory biomarker assessed with** | | | | |
| --- | --- | --- | --- | --- | --- | --- | --- | --- | --- |
| **Inflammatory biomarker** | **Reference**  **(first author, year)** | **Cohort** | **Cohort n** | **Timepoint clinical score** | **MMSE** | **CDR-SB** | **DRS-2** | **ADAS-COG** | **Other** |
| AAT (SERPINA1) | Pillai et al. 2020 | MCI-AD (*discovery*)  MCI (*validation*) | 48  134 | BL  9 months  15months  BL  12 months | δMMSE 9m: multivariable  Coefficient: 1.50  95%CI: -0.85 - 3.85  *p* = 0.20  δMMSE 15m: multivariable  Coefficient: 1.53  95%CI: -1.24 - 4.30  *p* = 0.27 | δCDR-SB: multivariable  Coefficient: 0.37  95%CI: -0.10 - 0.85  *p* = 0.12 |  |  |  |
| MCP-1 (CCL2) | Westin et al. 2012  Pillai et al. 2020 | MCI-AD  MCI-AD (*discovery*)  MCI (*validation*)  - MCI-AD | 47  48  134  - 97 | 5^*^ years  BL  9 months  15months  BL  12 months  24 months  36 months | Annual decrease MMSE  R_S_ = 0.42  ***p* = 0.004**  Annual decrease MMSE: multivariate  Β = 0.39  t = 2.70  ***p* = 0.009**  δMMSE 9m: multivariable  Coefficient: -1.54  95%CI: -4.09 - 1.02  *p* = 0.23  δMMSE 15m: multivariable  Coefficient: -2.36  95%CI: -5.47 - 0.76  *p* = 0.13  δMMSE 12m: multivariable  Coefficient: -1.45  95%CI: -2.54 - -0.36  ***p* = 0.009**  δMMSE 24m: multivariable  Coefficient: -1.46  95%CI: -3.19 - 0.26  *p* = 0.096  δMMSE 36m: multivariable  Coefficient: -2.87  95%CI: -5.16 - -0.58  ***p* = 0.015** | δCDR-SB 9m: multivariable  Coefficient: 1.23  95%CI: 0.24 - 2.22  ***p* = 0.016**  δCDR-SB 15m  (*n* = 40)  R_P_ = 0.54  ***p* = 0.015**  δCDR-SB 15m: multivariable  Coefficient: 2.82  95%CI: 1.30 - 4.34  ***p* < 0.001**  δCDR-SB 24m: multivariable  Coefficient: 0.86  95%CI: -0.04 - 1.75  *p* = 0.062  δCDR-SB 36m :  R_P_ = 0.24  ***p* = 0.024**  δCDR-SB 36m: MCI-AD (*n* = 97)  R_P_ = 0.27  ***p* = 0.038**  δCDR-SB 36m: multivariable  Coefficient: 1.43  95%CI: 0.13 - 2.73  ***p* = 0.031** |  |  |  |
| CCL4 | Pillai et al. 2020 | MCI-AD (*discovery*)  MCI (*validation*) | 48  134 | BL  15months  BL  12 months  36 months | δMMSE: multivariable  Coefficient: 1.48  95%CI: -0.15 - 3.12  *p* = 0.073  δMMSE 36m: multivariable  Coefficient: -1.36  95%CI: -2.71 - 0.00  *p* = 0.050 | δCDR-SB 12m  R_P_ = 0.18  *p* = 0.36  δCDR-SB 12m: multivariable  Coefficient: 0.30  95%CI: -0.03 - 0.63  *p* = 0.071  δCDR-SB 36m: multivariable  Coefficient: 0.76  95%CI: 0.01 - 1.52  ***p* = 0.047** |  |  |  |
| CCL5 (RANTES) | Pillai et al. 2020 | MCI (*validation*) | 134 | BL  24 months  36 months | δMMSE24m: multivariable  Coefficient: -0.43  95%CI: -1.18 - 0.31  *p* = 0.25  δMMSE 36m: multivariable  Coefficient: 0.74  95%CI: -0.50 - 1.98  *p* = 0.24 | δCDR-SB 24m  (*n* = 118)  R_P_ = 0.19  *p* = 0.67  δCDR-SB 24m: multivariable  Coefficient: 0.41  95%CI: 0.02 - 0.79  ***p* = 0.041** |  |  |  |
| CCL13 | Westin et al. 2012 | MCI-AD | 47 | 5^*^ years | Annual decrease MMSE  R_S_ = NR  *p* > 0.05 |  |  |  |  |
| CCL26 | Westin et al. 2012 | MCI-AD | 47 | 5^*^ years | Annual decrease MMSE  R_S_ = NR  *p* > 0.05 |  |  |  |  |
| Complement factor C3 | Toledo et al. 2014 | MCI  AD | 163  83 | 184^^^ weeks  98.3^^^ weeks | C3xTime  Β = 0.40  *p* = 0.16  C3xTime  Β = -0.009  *p* = 1.0 |  |  | C3xTime  Β = -0.12  ***p* = 0.041**  C3xTime  Β = -0.0089  *p* = 1.0 | Composite memory: C3xTime  Β = 0.067  *p* = 0.051  Composite executive function: C3xTime  Β = 0.028  *p* = 0.70  Composite memory: C3xTime  Β = 0.085  *p* = 0.18  Composite executive function: C3xTime  Β = 0.12  *p* = 0.092 |
| YKL-40 (CHI3L1) | Kester et al. 2015 | MCI  AD | 61  65 | 2.7^^^ years  3.8^^^ years | MMSE decline predictor  Β = -0.32  SE = 0.19  *p* = 0.11  MMSE decline predictor  Β = 0.65  SE = 0.29  ***p* = 0.03**  YKL40 Highest tertile AD  MMSE decline 1.5 per year  YKL40 Lowest tertile AD  MMSE decline 2.8 per year |  |  |  |  |
| CRP | Pillai et al. 2020 | MCI (*validation*) | 134 | BL  24 months | δMMSE: multivariable  Coefficient: -0.26  95%CI: -0.65 - 0.14  *p* = 0.20 |  |  |  |  |
| Eotaxin (CCL11) | Taipa et al. 2019  Westin et al. 2012 | AD  MCI-AD | 32  47 | BL  1 year  5^*^ years | Annual decrease MMSE  R_S_ = NR  *p* > 0.05 |  | δDRS-2  R_?_ = - 0.395  *p* > 0.05 |  |  |
| Factor H | Toledo et al. 2014 | MCI  AD | 163  83 | 184^^^ weeks  98.3^^^ weeks | FHxTime  Β = 0.29  *p* = 0.15  FHxTime  Β = -0.0072  *p* = 1.0 |  |  | FHxTime  Β = -0.075  ***p* = 0.041**  FHxTime  Β = -0.0052  *p* = 1.0 | Composite memory: FHxTime  Β = 0.037  *p* = 0.089  Composite executive function: FHxTime  Β = 0.26  *p* = 0.51  Composite memory: FHxTime  Β = 0.048  *p* = 0.18  Composite executive function: FHxTime  Β = 0.072  *p* = 0.085 |
| FGF basic | Taipa et al. 2019 | AD | 32 | BL  1 year |  |  | δDRS-2  R_?_ = - 0.664  ***p* < 0.01** |  |  |
| Fibrinogen (FGA) | Pillai et al. 2020 | MCI-AD (*discovery*)  MCI (*validation*) | 48  134 | BL  15months  BL  12 months | δMMSE: multivariable  Coefficient: 0.49  95%CI: 0.13 - 0.86  ***p* = 0.008** | δCDR-SB: multivariable  Coefficient: -0.27  95%CI: -0.73 - 0.20  *p* = 0.25  δCDR-SB  R_P_ = -0.18  *p* = 0.36  δCDR-SB: multivariable  Coefficient: -0.23  95%CI: -0.40 - -0.05  ***p* = 0.011** |  |  |  |
| Gas-6 | Sainaghi et al. 2017 | AD | 50 | 2 years | MMSE decline  R_S_ = -0.80  ***p* < 0.0001** |  |  |  |  |
| G-CSF | Taipa et al. 2019 | AD | 32 | BL  1 year |  |  | δDRS-2  R_?_ = - 0.521  ***p* < 0.05** |  |  |
| GM-CSF | Tarkowski et al. 2003  Taipa et al. 2019 | MCI  AD | 56  32 | BL  9 months  BL  1 year | MMSE 9m  R_S_ = NR  *p* > 0.05 |  | δDRS-2  R_?_ = - 0.479  ***p* < 0.05** |  |  |
| Haptoglobin | Pillai et al. 2020 | MCI-AD (*discovery*) | 37 | BL  15months | δMMSE:  R_P_ = 0.36  *p* = 0.50 |  |  |  |  |
| ICAM-1 | Zhao et al. 2020 | MCI | 116 | 30.2^^^ months | MMSE decline predictor  NR  *p* > 0.05 |  |  | ADAS-Cog decline predictor  NR  *p* > 0.05 |  |
| IFN-ɣ | Taipa et al. 2019 | AD | 32 | BL  1 year |  |  | δDRS-2  R_?_ = - 0.495  ***p* < 0.05** |  |  |
| IL-1β | Tarkowski et al. 2003  Taipa et al. 2019 | MCI  AD | 56  32 | BL  9 months  BL  1 year | MMSE 9m  R_S_ = 0.34  ***p* = 0.001**  δMMSE  R_S_ = 0.30  ***p* = 0.03** |  | δDRS-2  R_?_ = - 0.576  ***p* < 0.01** |  |  |
| IL-1Ra (IL1RN) | Taipa et al. 2019 | AD | 32 | BL  1 year |  |  | δDRS-2  R_?_ = - 0.177  *p* > 0.05 |  |  |
| IL-2 | Taipa et al. 2019 | AD | 32 | BL  1 year |  |  | δDRS-2  R_?_ = - 0.440  *p* > 0.05 |  |  |
| IL-4 | Taipa et al. 2019 | AD | 32 | BL  1 year |  |  | δDRS-2  R_?_ = - 0.507  ***p* < 0.05** |  |  |
| IL-5 | Taipa et al. 2019 | AD | 32 | BL  1 year |  |  | δDRS-2  R_?_ = - 0.365  *p* > 0.05 |  |  |
| IL-6 | Taipa et al. 2019 | AD | 32 | BL  1 year |  |  | δDRS-2  R_?_ = - 0.553  ***p* < 0.05** |  |  |
| IL-7 | Taipa et al. 2019 | AD | 32 | BL  1 year |  |  | δDRS-2  R_?_ = - 0.367  *p* > 0.05 |  |  |
| IL-9 | Taipa et al. 2019 | AD | 32 | BL  1 year |  |  | δDRS-2  R_?_ = - 0.578  ***p* < 0.01** |  |  |
| IL-10 | Taipa et al. 2019 | AD | 32 | BL  1 year |  |  | δDRS-2  R_?_ = - 0.376  *p* > 0.05 |  |  |
| IL-12 | Taipa et al. 2019 | AD | 32 | BL  1 year |  |  | δDRS-2  R_?_ = - 0.391  *p* > 0.05 |  |  |
| IL-13 | Taipa et al. 2019 | AD | 32 | BL  1 year |  |  | δDRS-2  R_?_ = - 0.277  *p* > 0.05 |  |  |
| IL-17 | Taipa et al. 2019 | AD | 32 | BL  1 year |  |  | δDRS-2  R_?_ = - 0.577  ***p* < 0.01** |  |  |
| MIP-1β | Taipa et al. 2019 | AD | 32 | BL  1 year |  |  | δDRS-2  R_?_ = - 0.577  ***p* < 0.01** |  |  |
| MMP-3 | Pillai et al. 2020 | MCI-AD (*discovery*)  MCI (*validation*) | 48  134 | BL  9 months  15months  BL  36 months | δMMSE 9m  (*n* = 40)  R_P_ = 0.38  *p* = 0.50  δMMSE 9m : multivariable  Coefficient: 2.05  95%CI: 0.32 - 3.78  ***p* = 0.022** | δCDR-SB 9m  (*n* = 39)  R_P_ = -0.36  *p* = 0.77  δCDR-SB 9m: multivariable  Coefficient: -0.97  95%CI: -1.64 - -0.29  ***p* = 0.006**  δCDR-SB 15m: multivariable  Coefficient: -0.94  95%CI: -1.82 - -0.05  ***p* = 0.039**  δCDR-SB : multivariable  Coefficient: -0.53  95%CI: -1.25 - 0.19  *p* = 0.15 |  |  |  |
| PDGF-BB | Taipa et al. 2019 | AD | 32 | BL  1 year |  |  | δDRS-2  R_?_ = -0.424  *p* > 0.05 |  |  |
| sTNFR2 | Pillai et al. 2021 | MCI  AD | 67  42 | 5.0^^^ years  3.0^^^ years |  | CDR-SB decline: sTNFR2  Beta = 0.829879  SE = 3.489585  *p* = 0.812  CDR-SB decline: Visit number x sTNFR2  Beta = 0.129465  SE = 0.720227  *p* = 0.18  CDR-SB decline: sTNFR2  Beta = 1.417211  SE = 4.269298  *p* = 0.741  CDR-SB decline: Visit number x sTNFR2  Beta = 2.457261  SE = 2.061383  *p* = 0.236  *p*FDR = 0.472 |  |  |  |
| sTNFR1-score (*PCA-score of sTNFR1-related protein = sTNFR1 + sTNFR2 + sVCAM-1 + sICAM-1*) | Hu et al. 2021 | MCI | 174 | 0 – 60 months |  | CDR-SB changes 0-60m :  Beta = -0.026  95%CI = -0.139 - 0.086  *p* = 0.642  CDR-SB changes 0-60m & sTNFR1 score x Months  Beta = -0.020  95%CI = -0.033-0.008  ***p* = 0.002**  Time to CDR > 4.0: High-AD score + Low-sTNFR1 score vs. High-AD score + High- sTNFR1 score  ***p* = 0.007**  Time to CDR > 4.0: Low-AD score + High-sTNFR1 score vs. High-AD score + High-sTNFR1  ***p* = 0.023**  Risk for CDR > 4.0: High AD score + High sTNFR1  HR = 0.454  95% CI = 0.265 - 0.778  ***p* = 0.004** |  |  | ADNI-Mem-EF changes 0-60m:  Beta = -0.010  95%CI = -0.042-0.022  *p* = 0.547  ADNI-Mem-EF changes 0-60m & sTNFR1 score x Months :  Beta = 0.005  95%CI = 0.002 - 0.008  ***p* < 0.001** |
| sTREM2 | Pillai et al. 2021 | MCI  AD | 67  42 | 5.0^^^ years  3.0^^^ years |  | CDR-SB decline: sTREM2  Beta = -0.42004  SE = 0.632681  *p* = 0.508  CDR-SB decline: Visit number x sTREM2  Beta = 0.015941  SE = 0.120067  *p* = 0.894  CDR-SB decline: sTREM2  Beta = 0.096417  SE = 0.961389  *p* = 0.92  CDR-SB decline: Visit number x sTREM2  Beta = -1.33428  SE = 0.47665  ***p* = 0.006**  ***p*FDR = 0.048** |  |  |  |
| sTREM2-score (*PCA of sTREM2 from two different analytical techniques*) | Hu et al. 2021 | AD | 97 | 0 – 36 months |  | CDR-SB changes 0-36m  Beta = 0.004  95%CI = -0.162-0.170  *p* = 0.964^£^  CDR-SB changes 0-36m & sTREM2 score x Months  Beta = -0.040  95%CI = -0.065-0.016  ***p* = 0.001**^£^  Time to CDR-SB > 7.8 High-AD score + high-sTREM2 score vs. low- sTREM2 score  ***p* = 0.001**  Time to CDR-SB > 7.8: High-pTau score + high-sTREM2 score vs. low-sTREM2 score  ***p* = 0.028**  Risk to CDR-SB > 7.8: High-AD score + High- sTREM2 score  HR = 0.412  95% CI = 0.193 - 0.878  ***p* = 0.022** |  |  | ADNI-Mem-EF changes 0-35m:  Beta = 0.027  95%CI = -0.008-0.062  *p* = 0.127^£^  ADNI-Mem-EF changes 0-36m & sTREM2 score x Months  Beta = 0.006  95%CI = 0.001 - 0.011  *p* = 0.015^£^ |
| TGF-β | Tarkowski et al. 2003 | MCI | 56 | BL  9 months | MMSE 9m  R_S_ = -0.03  *p* > 0.05 |  |  |  |  |
| TNF-α | Tarkowski et al. 2003  Zhao et al. 2020 | MCI  MCI | 56  116 | BL  9 months  30.2^^^ months | MMSE 9m  R_S_ = 0.03  *p* > 0.05  MMSE decline predictor  NR  *p* > 0.05 |  |  | ADAS-Cog decline predictor  NR  *p* > 0.05 |  |
| VCAM-1 | Pillai et al. 2020  Zhao et al. 2020 | MCI-AD (*discovery*)  MCI | 40  116 | BL  15months  30.2^^^ months | δMMSE  R_P_ = -0.34  *p* = 0.50  MMSE decline predictor  NR  *p* > 0.05 |  |  | ADAS-Cog decline predictor  NR  *p* > 0.05 |  |

**Significant results are reported in bold**

***Legend***

^*^ Median

^^^ Mean

^$^ Not specified whether clinical or radiological disease reactivation

^£^ Significance set at *p* < 0.00625 to correct for multiple comparisons

R_S_ Spearman correlation

R_P_ Pearson correlation

R_Par_ Partial correlation

R_?_ Spearman or Pearson correlation

***Abbreviations***

95%CI 95% Confidence Interval

AD Alzheimer’s disease

BL Baseline

CIS Clinically isolated syndrome

HR Hazard ratio

MCI Mild cognitive impairment

MCI-AD MCI patients progressed towards AD

MS Multiple sclerosis

MS-converters CIS patients converted towards MS

n Number

NR Not reported

OR Odds ratio

PCA Principal Component Analysis

PMS Progressive multiple sclerosis

RIS Radiologically isolated syndrome

RNFL_PMB Papillomacular bundle retinal nerve fibre layer thickness

RNFL_T Temporal segment retinal nerve fibre layer thickness

RRMS Relapsing-remitting multiple sclerosis

SD Standard deviation

SE Standard error

SPMS Secondary progressive multiple sclerosis

***Clinical scores***

ADAS-Cog Alzheimer's Disease Assessment Scale-Cognitive subscale

ADNI-Mem-EF Average of composite Alzheimer's Disease Neuroimaging Initiative Memory and Executive Function scores (ADNI-Mem-EF)

BRB Brief Repeatable Battery of Neuropsychological Tests

BREMS Bayesian Risk Estimate for Multiple Sclerosis

CDR-SB Clinical Dementia Rating – Sum of Boxes

DRS-2 Dementia Rating Scale-2

EDSS Expanded Disability Status Scale

MMSE Mini-Mental State Exam

MSFC Multiple Sclerosis Functional Composite

MSSS Multiple Sclerosis Severity Score

PI Progression index (EDSS / disease duration)

SDMT Symbol Digit Modalities Test
