# Supplementary material for: Cerebrospinal fluid inflammatory biomarkers for disease progression in Alzheimer’s disease and multiple sclerosis: a systematic review
Source: Front Immunol. 2023 Jul 13;14:1162340. doi: 10.3389/fimmu.2023.1162340 (PMC10374015; doi:10.3389/fimmu.2023.1162340)
Supplement: Supplementary file 7 [file Table_6.docx]

**Cerebrospinal fluid inflammatory biomarkers for disease progression in Alzheimer’s disease and multiple sclerosis: a systematic review**

**Joke Temmerman^1,2,3^, Sebastiaan Engelborghs^1,2,3^, Maria Bjerke^1,2,3,4^*, Miguel D’Haeseleer^1,3,5^***

1. Vrije Universiteit Brussel, Center for Neurosciences (C4N); Laarbeeklaan 103, 1090 Jette, Brussels, Belgium.

2. Universiteit Antwerpen, Department of Biomedical Sciences and Institute Born-Bunge, Reference Center for Biological Markers of Dementia (BIODEM); Universiteitsplein 1, 2610 Wilrijk, Antwerp, Belgium.

3. Universitair Ziekenhuis Brussel, Department of Neurology; Laarbeeklaan 101, 1090 Jette, Brussels, Belgium.

4. Universitair Ziekenhuis Brussel, Department of Clinical Biology, Laboratory of Clinical Neurochemistry; Laarbeeklaan 101, 1090 Jette, Brussels, Belgium.

5. Nationaal Multiple Sclerose Centrum (NMSC); Vanheylenstraat 16, 1820 Melsbroek, Steenokkerzeel, Belgium.

Corresponding author: [miguel.dhaeseleer@uzbrussel.be](mailto:miguel.dhaeseleer@uzbrussel.be)

CSF inflammatory markers and transition from one disease stage to a more severe disease stage

| ***MS*** | | | | | **FUP cohort** | | **Converters vs. Nonconverters statistics** | | | |
| --- | --- | --- | --- | --- | --- | --- | --- | --- | --- | --- |
| **Inflammatory biomarker** | **Reference**  **(first author, year)** | **Cohort BL** | **Cohort n** | **FUP duration** | **Converters n** | **Non-converters n** | **Between-group comparison** | **ROC – AUC measures** | **Cox proportional Hazard**  **Kaplan-Meier** | **Logistic regression** |
| Κ-FLC | Makshakov et al. 2015  Voortman et al. 2017 | CIS  CIS | 139  48 | 2 years  4.8^*^ years | 98  23 | 41  25 | ***p* < 0.00001^1^**  *p* > 0.05^1^ |  |  |  |
| Κ-FLC index (*corrected for blood-CSF barrier permeability*) | Vecchio et al. 2020  Gaetani et al. 2020 | RIS+CIS  CIS | 18  19 | 3.6^^^ years  39.1^*^ months | 6  12 | 12  7 | ***p* = 0.03^1^** |  | Time to conversion  Cut-off = 10.6  C = 0.63  ***p* = 0.02**  50% of patients with κ-FLC index > 10.6 converting in 21 months | Risk of conversion  HR = 1.07  95%CI = 0.99–1.16  *p* = 0.09 |
| Κ-FLC / Λ -FLC ratio | Voortman et al. 2017  Rathbone et al. 2018 | CIS  CIS | 48  43 | 4.8^*^ years  5 years | 23  NR | 25  NR | ***p* < 0.05^1^**  *p* > 0.05**^1^** |  | Risk of conversion  Cut-off = 3.38  HR = 2.89  95%CI = 1.17 – 7.14  ***p* = 0.016** | Likelihood of conversion  Cut-off = 3.38  OR = 4.86  95%CI = 1.43 – 16.50  ***p* = 0.011** |
| Κ-FLC / IgG ratio | Vecchio et al. 2020 | RIS+CIS | 18 | 3.6^^^ years | 6 | 12 | ***p* = 0.003^1^** |  |  | Risk of conversion  HR = 1.05  95%CI = 1.01 – 1.10  ***p* = 0.02** |
| Λ-FLC | Makshakov et al. 2015  Voortman et al. 2017 | CIS  CIS | 139  48 | 2 years  4.8^*^ years | 98  23 | 41  25 | ***p* < 0.01^1^**  *p* > 0.05^1^ |  |  |  |
| Λ-FLC-index  (*corrected for blood-CSF barrier permeability*) | Vecchio et al. 2020  Gaetani et al. 2020 | RIS+CIS  CIS | 18  19 | 3.6^^^ years  39.1^*^ months | 6  12 | 12  7 | *p* > 0.05**^1^** |  | Time to conversion  *p* > 0.05 |  |
| Λ-FLC / IgG ratio | Vecchio et al. 2020 | RIS+CIS | 18 | 3.6^^^ years | 6 | 12 | *p* > 0.05**^1^** |  |  |  |
| A1AG1 | Borras et al. 2016 | CIS | 50 | 4.08^*^ years *(converters)*  3.25* years  *(non-converters)* | 25 | 25 | ***p* < 0.0001**² |  |  |  |
| A1AG1var | Borras et al. 2016 | CIS | 50 | 4.08^*^ years *(converters)*  3.25^*^ years  *(non-converters)* | 25 | 25 | *p* > 0.05² |  |  |  |
| A1AG1 + AACT-2 | Borras et al. 2016 | CIS | 50 | 4.08^*^ years *(converters)*  3.25* years  *(non-converters)* | 25 | 25 |  | AUC = 0.79  Sensitivity = 0.76  Specificity = 0.75 |  |  |
| A2MG | Borras et al. 2016 | CIS | 50 | 4.08^*^ years *(converters)*  3.25* years  *(non-converters)* | 25 | 25 | *p* > 0.05² |  |  |  |
| AACT1 | Borras et al. 2016 | CIS | 50 | 4.08^*^ years *(converters)*  3.25* years  *(non-converters)* | 25 | 25 | ***p* < 0.0003**² |  |  |  |
| AACT2 | Borras et al. 2016 | CIS | 50 | 4.08^*^ years *(converters)*  3.25* years  *(non-converters)* | 25 | 25 | ***p* < 0.0001**² |  |  |  |
| AACT3 | Borras et al. 2016 | CIS | 50 | 4.08^*^ years *(converters)*  3.25^*^ years  *(non-converters)* | 25 | 25 | *p* > 0.05² |  |  |  |
| BAFF | De Fino et al. 2019 | CIS | 24 | 19.6^^^ months | 10 | 14 | *p* > 0.05^1^ |  |  |  |
| YKL-40 (CHI3L1) | Comabella et al. 2010  Borras et al. 2016  De Fino et al. 2019  Gil-Perotin et al. 2019  Thouvenot et al. 2019 | CIS (*first validation*)  CIS (*second validation*)  CIS  CIS  RRMS  RIS | 84  52  50  24  99  71 | 5.5^^^ years (*converters*)  4.1^^^ years (*non-converters*)  4.9^^^ years (*converters*)  2.3^^^ years (*non-converters*)  4.08^*^ years *(converters)*  3.25^*^ years  *(non-converters)*  19.6^^^ months  4.4^^^ years  16^^^ months | 48  26  25  10  14  20 | 36  26  25  14  85  51 | ***p* = 2.3*10^(-5)^** ^1^  ***p* = 0.018**^1^  ***p* < 0.0001**²  ***p* = 0.031**^1^  *p* = 0.097^1^ | AUC = 0.79  Range = 0.67 – 0.90  ***p* = 0.002**  Cut-off = 154.6 mg/ml | Cut-off = 287.9 mg/ml  Time to conversion: low vs. high  **Log-rank *p* = 0.003**  Risk of conversion  HR = 2.5  95%CI = 1.3 – 4.7  ***p* = 0.004**  Progression to SPMS: Multivariate  Estimate = 2.839  SD = 1.050  HR = 18.044  95%CI = 2.31 – 141.3  ***p* = 0.0002**  Risk of conversion: univariate (*n* = 68)  HR = 2.13  95%CI = 0.77 – 5.87  *p* = 0.145  Risk of conversion: multivariate (*n* = 65)  HR = 1.56  95%CI = 0.52 – 4.71  *p* = 0.432 |  |
| YKL-40 + CLUS | Borras et al. 2016 | CIS | 50 | 4.08^*^ years *(converters)*  3.25^*^ years  *(non-converters)* | 25 | 25 |  | AUC = 0.83  Sensitivity = 0.76  Specificity = 0.83 |  |  |
| YKL-40+ CNDP1 | Borras et al. 2016 | CIS | 50 | 4.08^*^ years *(converters)*  3.25^*^ years  *(non-converters)* | 25 | 25 |  | AUC = 0.86  Sensitivity = 0.84  Specificity = 0.83 |  |  |
| YKL-40 + CNDP1 + SEM7A | Borras et al. 2016 | CIS | 50 | 4.08^*^ years *(converters)*  3.25^*^ years  *(non-converters)* | 25 | 25 |  | AUC = 0.86  Sensitivity = 0.84  Specificity = 0.87 |  |  |
| CLUS | Borras et al. 2016 | CIS | 50 | 4.08^*^ years *(converters)*  3.25^*^ years  *(non-converters)* | 25 | 25 | ***p* < 0.005**² |  |  |  |
| CMGA | Borras et al. 2016 | CIS | 50 | 4.08^*^ years *(converters)*  3.25^*^ years  *(non-converters)* | 25 | 25 | *p* > 0.05² |  |  |  |
| CNDP1 | Borras et al. 2016 | CIS | 50 | 4.08^*^ years *(converters)*  3.25^*^ years  *(non-converters)* | 25 | 25 | ***p* < 0.0001**² |  |  |  |
| CNTN1 | Borras et al. 2016 | CIS | 50 | 4.08^*^ years *(converters)*  3.25^*^ years  *(non-converters)* | 25 | 25 | *p* > 0.05² |  |  |  |
| Complement factor C3 | Borras et al. 2016 | CIS | 50 | 4.08^*^ years *(converters)*  3.25^*^ years  *(non-converters)* | 25 | 25 | *p* > 0.05² |  |  |  |
| CXCL8 (IL-8) | Rossi et al. 2015 | RIS  CIS | 18  39 | 11^*^ months  10^*^ months | 10  23 | 8  16 | ***p* = 0.03**  ***p* = 0.001** |  |  | Multivariate  Cut-off = 1000 pg/ml  Coefficient = 3.99  SE = 1.66  OR = 54.44  95%CI = 1.06 – 1435.38  ***p* = 0.01**  Multivariate  Cut-off = 1000 pg/ml  Coefficient = 2.29  SE = 0.97  OR = 9.97  95%CI = 1.46 – 67.87  ***p* = 0.02** |
| CXCL13 | Brettschneider et al. 2010  Ferraro et al. 2015  Olesen et al. 2019 | CIS  CIS  ON | 91  110  40 | 2 years  40^*^ months  28.2^*^ months (*converters*)  29.6^*^ months (*non-converters*) | 45  94  16 | 46  16  24 | ***p* < 0.05**^1^  ***p* < 0.0001**^1^  ***q* = 0.0006**^1^ | Cut-off = 7.7pg/ml  AUC = 0.64 Sensitivity = 0.62  Specificity = 0.76  PPV = 0.70  NPV = 0.69  Cut-off = 15.4 pg/ml  AUC = 0.64  Sensitivity = 0.61  Specificity = 0.67  Cut-off = 37 pg/ml  AUC = 0.72  95%CI = 0.55 – 0.90  Sensitivity = 0.50  Specificity = 0.95 | Risk of conversion: univariate  HR = 2.9  95%CI = 1.2 – 3.9  ***p* = 0.007**  Risk of conversion: multivariate  ***p* = 0.045** | Predictor MS  OR = 1.05  95%CI = 1.00 – 1.10  ***p* = 0.030** |
| CXCL13 + OCB | Brettschneider et al. 2010 | CIS | 91 | 2 years | 45 | 46 |  | Sensitivity = 0.60  Specificity = 0.78  PPV = 0.71  NPV = 0.63 |  |  |
| CXCL13 + MRZ | Brettschneider et al. 2010 | CIS | 91 | 2 years | 45 | 46 |  | Sensitivity = 0.32  Specificity = 0.90  PPV = 0.77  NPV = 0.57 |  |  |
| CYTC | Borras et al. 2016 | CIS | 50 | 4.08^*^ years *(converters)*  3.25^*^ years  *(non-converters)* | 25 | 25 | *p* > 0.05² |  |  |  |
| GFAP | Avsar et al. 2012 | CIS | 46 | 4.5^^^ years | 9 | 37 | *p* > 0.05^3^ | Converters vs. Non-converters vs. RRMS  AUC = 0.81  Accuracy = 67.07%  k = 5  ***p* < 0.05** |  |  |
| Haptoglobin | Borras et al. 2016 | CIS | 50 | 4.08^*^ years *(converters)*  3.25^*^ years  *(non-converters)* | 25 | 25 | *p* > 0.05² |  |  |  |
| HOXB3 | Timirci-Kahraman et al. 2019 | CIS | 42 | 78.3^^^ months (*converters*)  96.4^^^ months (*non-converters*) | 23 | 19 | ***p* < 0.05**^3^ |  |  |  |
| IgG intrathecal (*>0.1 mg/dL (IgG ), >0.7 (IgG Index) or > 3.3 mg/24 h (IgG SR*)) | Tumani et al. 1998 | ON | 36 | 4 years | 18 | 18 |  | Four cell method  Sensitivity = 0.44  Specificity = 0.56  PPV = 0.50  NPV = 0.50 |  |  |
| IgG index | Olesen et al. 2019  Cinar et al. 2018 | ON  CIS | 40  41 | 28.2^*^ months (*converters*)  29.6^*^ months (*non-converters*)  12.8^^^ months | 16  35 | 24  6 | ***q* = 0.0052** | Cut-off = 0.64  Sensitivity = 0.75  Specificity = 0.82  Cut-off = 0.7  Sensitivity = 0.69  Specificity = 0.50  PPV = 0.89  NPV = 0.21  Accuracy = 0.66 | Risk of conversion  Cut-off = 0.7  HR = 0.997  *p* = 0.992  Time to conversion  Cut-off = 0.7  *p* = 0.092 | Predictor MS  OR = 11.09  95%CI = 1.58 – 77.59  ***p* = 0.015** |
| IgG index + OCB + Leukocytes | Olesen et al. 2019 | ON | 40 | 28.2^*^ months (*converters*)  29.6^*^ months (*non-converters*) | 16 | 24 |  | AUC = 0.86  Optimism AUC = 0.83  95%CI = 0.74 – 1.00 |  |  |
| IgITS (> 2 bands OCB and / or raised IgG index) | Ruet et al. 2010 | APTM | 106 | 4.3^^^ years | 78 | 28 |  | Sensitivity = 0.78  Specificity = 0.48  PPV = 0.81  NPV = 0.45  Accuracy = 0.70 |  | Univariate  OR = 3.37  95%CI = 1.32 – 8.58  ***p* = 0.01**  Multivariate  OR = 4.09  95%CI = 1.32 – 12.67  ***p* = 0.004** |
| IgG oligoclonal bands (OCB) | Tumani et al. 1998  Koch et al. 2007  Brettschneider et al. 2010  Farina et al. 2017  Cinar et al. 2018  Olesen et al. 2019  Gaetani 2020  Kolcava et al. 2020 | ON  RRMS  CIS  RRMS OCB+  RRMS OCB-  CIS  CIS (*OCB measure*)  ON  CIS  CIS | 36  78  91  50  40  41  28  40  19  64 | 4 years  5 years  2 years  10 years  10 years  12.8^^^ months  28.2^*^ months (*converters*)  29.6^*^ months (*non-converters*)  39.1^*^ months  27^*^ months | 18  19  45  15  4  35  26  16  12  45 (*MS*)  -29 (*CDMS*)  -~~16 (~~*~~MRI+~~*~~)~~ | 18  59  46  35  36  6  2  24  7  19 | Number OCB  *p* = 0.31  OCB+ vs. OCB-  *p* = 0.5  OCB+ vs. OCB-  ***p* = 0.018**^3^  Time to conversion: OCB+ vs. OCB-  ***p* < 0.0001**  Time to conversion: OCB+ vs. OCB-  *p* = 0.20  OCB+  ***q* = 0.0021**^1^  OCB+**:** Nonconverters vs. CDMS  ***p* = 0.002**^1^ | Four cell method  Sensitivity = 0.83  Specificity = 0.33  PPV = 0.56  NPV = 0.67  Sensitivity = 0.91  Specificity = 0.36  PPV = 0.59  NPV = 0.81  OCB+  Sensitivity = 0.74  Specificity = 0.67  PPV = 0.93  NPV = 0.31  Accuracy = 0.73  AUC = 0.78  95%CI = 0.63 – 0.93  Sensitivity = 0.81  Specificity = 0.75 | Risk of conversion: OCB+  HR = 2.16  *p* = 0.052  Time to conversion  *p* > 0.05  Risk of conversion CDMS univariate: OCB+  HR = 2.898  95%CI = 1.166 - 7.201  ***p* = 0.022**  Risk of conversion CDMS multivariate: OCB+  HR = 2.348  95%CI = 0.918 - 6.004  *p* = 0.075 | Predictor MS  OR = 13.00  95%CI = 2.74 – 61.79  ***p* = 0.001**  CDMS predictor univariate: OCB+  OR = 8.306  95%CI = 2.218 - 31.098  ***p* = 0.002**  CDMS predictor multivariate: OCB+  OR = 26.599  95%CI = 2.868 - 246.665  ***p* = 0.004** |
| IgG oligoclonal bands (OCB) + MRZ reaction | Tumani et al. 1998  Brettschneider et al. 2010 | ON  CIS | 36  91 | 4 years  2 years | 18  45 | 18  46 |  | Four cell method  Sensitivity = 0.56  Specificity = 0.33  PPV = 0.63  NPV = 0.75  Sensitivity = 0.39  Specificity = 0.74  PPV = 0.62  NPV = 0.53 |  |  |
| IL-1β | Olesen et al. 2019 | ON | 40 | 28.2^*^ months (*converters*)  29.6^*^ months (*non-converters*) | 16 | 24 |  | Cut-off: 0.20 pg/ml  Sensitivity = 0.47  Specificity = 0.65 |  | Predictor MS  OR = 1.11  95%CI = 0.14 - 8.99  *p* = 0.923 |
| IL-6 | Olesen et al. 2019 | ON | 40 | 28.2^*^ months (*converters*)  29.6^*^ months (*non-converters*) | 16 | 24 |  | Cut-off: 2.67 pg/ml  Sensitivity = 0.67  Specificity = 0.75 |  | Predictor MS  OR = 1.03  95%CI = 0.86 - 1.22  *p* = 0.772 |
| IL-10 | Olesen et al. 2019 | ON | 40 | 28.2^*^ months (*converters*)  29.6^*^ months (*non-converters*) | 16 | 24 | ***q* = 0.04**^1^ | Cut-off: 0.21 pg/ml  AUC = 0.81  95%CI = 0.66 – 0.96  Sensitivity = 0.60  Specificity = 0.94 |  | Predictor MS  OR = 1 942  95%CI = 1.03 - 10^6^  ***p* = 0.049** |
| IL-17a | Olesen et al. 2019 | ON | 40 | 28.2^*^ months (*converters*)  29.6^*^ months (*non-converters*) | 16 | 24 |  | Cut-off: 0.017 pg/ml  Sensitivity = 0.93  Specificity = 0.47 |  | Predictor MS  OR = 18.18  95%CI = 10^(-6)^ - 10^6^  *p* = 0.778 |
| KLK6 | Borras et al. 2016 | CIS | 50 | 4.08^*^ years *(converters)*  3.25^*^ years  *(non-converters)* | 25 | 25 | *p* > 0.05² |  |  |  |
| KLK6-2 | Borras et al. 2016 | CIS | 50 | 4.08^*^ years *(converters)*  3.25^*^ years  *(non-converters)* | 25 | 25 | ***p* < 0.0007**² |  |  |  |
| Leukocytes | Olesen et al. 2019 | ON | 40 | 28.2^*^ months (*converters*)  29.6^*^ months (*non-converters*) | 16 | 24 |  | Cut-off = 6/µL  AUC = 0.84  95%CI = 0.70 – 0.97  Sensitivity = 0.75  Specificity = 0.83 |  | Predictor MS  OR = 1.09  95%CI = 1.02 – 1.156  ***p* = 0.007** |
| MBP | Avsar et al. 2012 | CIS | 46 | 4.5^^^ years | 9 | 37 | NR |  |  |  |
| MOG | Avsar et al. 2012 | CIS | 46 | 4.5^^^ years | 9 | 37 | *p* > 0.05^3^ |  |  |  |
| MRZ reaction | Tumani et al. 1998  Brettschneider et al. 2010 | ON  CIS | 36  91 | 4 years  2 years | 18  45 | 18  46 |  | Four cell method  Sensitivity = 0.61  Specificity = 0.72  PPV = 0.69  NPV = 0.65  Sensitivity = 0.42  Specificity = 0.74  PPV = 0.64  NPV = 0.55 |  |  |
| MUC18 | Borras et al. 2016 | CIS | 50 | 4.08^*^ years *(converters)*  3.25^*^ years  *(non-converters)* | 25 | 25 | *p* > 0.05² |  |  |  |
| OPN | Borras et al. 2016  De Fino et al. 2019 | CIS  CIS | 50  24 | 4.08^*^ years *(converters)*  3.25^*^ years  *(non-converters)*  19.6^^^ months | 25  10 | 25  14 | *p* > 0.05²  *p* = 0.320^1^ | AUC = 0.72  Range = 0.58 – 0.87  ***p* = 0.018** |  |  |
| PGCB | Borras et al. 2016 | CIS | 50 | 4.08^*^ years *(converters)*  3.25^*^ years  *(non-converters)* | 25 | 25 | *p* > 0.05² |  |  |  |
| Pleocytosis *( >5 leukocytes/µL CSF*) | Kolcava et al. 2020 | CIS | 64 | 27^*^ months | 45 (*MS*)  -29 (*CDMS*)  -~~16 (~~*~~MRI+~~*~~)~~ | 19 | Pleocytosis+: Nonconverters vs. CDMS  *p* = 0.049^1^ |  | Risk of conversion CDMS univariate: Pleocytosis+  HR = 2.327  95%CI = 1.084 - 4.998  ***p* = 0.030** | CDMS predictor univariate: Pleocytosis+  OR = 5.194  95%CI = 1.002 - 26.940  *p* = 0.050  CDMS predictor multivariate: Pleocytosis+  OR = 5.941  95%CI = 1.664 - 21.207  ***p* = 0.006** |
| PON1 | Borras et al. 2016 | CIS | 50 | 4.08^*^ years *(converters)*  3.25^*^ years  *(non-converters)* | 25 | 25 | *p* > 0.05² |  |  |  |
| “Positive CSF” = OCB presence and/or IgG index > 0.7 | Thouvenot et al. 2019  Cinar et al. 2018 | RIS  CIS  CIS (*OCB measure*) | 71  41  28 | 16^*^ months  12.8^^^ months | 20  35  26 | 51  6  2 | ***p* = 0.044**  ***p* = 0.035** | Sensitivity = 0.94  Specificity = 0.33  PPV = 0.89  NPV = 0.50  Accuracy = 0.85 | Risk of conversion : univariate (*n* = 70)  HR = 2.9  95%CI = 0.83 – 10.2  *p* = 0.097  Risk of conversion : multivariate (*n* = 65)  HR = 2.22  95%CI = 0.57 – 8.59  *p* = 0.127 |  |
| Q-κ | Makshakov et al. 2015 | CIS | 139 | 2 years | 98 | 41 | ***p* < 0.00001**^1^ |  |  |  |
| Q-λ | Makshakov et al. 2015 | CIS | 139 | 2 years | 98 | 41 | ***p* < 0.01**^1^ |  |  |  |
| sCD163 | De Fino et al. 2019 | CIS | 24 | 19.6^^^ months | 10 | 14 | ***p* = 0.038** | AUC = 0.87  Range = 0.76 – 0.98  ***p* < 0.001** |  |  |
| SCG2 | Borras et al. 2016 | CIS | 50 | 4.08^*^ years *(converters)*  3.25^*^ years  *(non-converters)* | 25 | 25 | *p* > 0.05² |  |  |  |
| SEM7A | Borras et al. 2016 | CIS | 50 | 4.08^*^ years *(converters)*  3.25^*^ years  *(non-converters)* | 25 | 25 | ***p* < 0.03**² |  |  |  |
| TNF-α | Olesen et al. 2019 | ON | 40 | 28.2^*^ months (*converters*)  29.6^*^ months (*non-converters*) | 16 | 24 | ***q* = 0.021**^1^ | Cut-off = 0.27 pg/ml  Sensitivity = 0.73  Specificity = 0.80 |  | Predictor MS  OR = 576  95%CI = 2.82 - 10^5  ***p* = 0.019** |
| TRAIL | Olesen et al. 2019 | ON | 40 | 28.2^*^ months (*converters*)  29.6^*^ months (*non-converters*) | 16 | 24 |  | Cut-off = 0.66 pg/ml  Sensitivity = 0.10  Specificity = 0.42 |  | Predictor MS  OR = 0.01  95%CI = 3*10^(-5)^ - 1.05  *p* = 0.052 |
| Transthyretine | Borras et al. 2016 | CIS | 50 | 4.08^*^ years *(converters)*  3.25^*^ years  *(non-converters)* | 25 | 25 | *p* > 0.05² |  |  |  |

| ***AD*** | | | | | **FUP cohort** | | **MCI-AD vs. sMCI statistics** | | | |
| --- | --- | --- | --- | --- | --- | --- | --- | --- | --- | --- |
| **Inflammatory biomarker** | **Reference**  **(first author, year)** | **Cohort BL** | **Cohort n** | **FUP duration** | **MCI-AD** | **sMCI** | **Between-group comparison** | **Cox proportional Hazard**  **Kaplan-Meier** | **Linear regression** | **Linear mixed model** |
| MCP-1 (CCL2) | Westin et al. 2012 | MCI | 119 | 5^*^ years | 47 | 52 | Cut-offs  292 - 584 pg/ml (*low*)  585 - 756 pg/ml (*mid*)  757 - 1369 pg/ml (*high*)  Time to progression: low vs. high  ***p* < 0.003** |  | Multivariate  ***p* = 0.016** |  |
| CCL13 | Westin et al. 2012 | MCI | 119 | 5^*^ years | 47 | 52 | *p* > 0.05^1^ |  |  |  |
| CCL26 | Westin et al. 2012 | MCI | 119 | 5^*^ years | 47 | 52 | *p* > 0.05^1^ |  |  |  |
| YKL-40 (CHI3L1) | Kester et al. 2015  Swanson et al. 2016 | MCI  MCI | 53  135 | 2.7^^^ years  24 months | 36  47 | 17  82 | ***p* < 0.01**^3^ | Risk of progression  HR = 1.003  95%CI = 1.001 – 1.006  ***p* = 0.02** |  | F = 0.358  *p* = 0.551 |
| Complement factor C3 | Toledo et al. 2014 | MCI | 160 | 184^^^  weeks | 79 | 81 |  | Risk of progression  HR = 0.62  *p* = 0.14 |  |  |
| Eotaxin (CCL11) | Westin et al. 2012 | MCI | 119 | 5^*^ years | 47 | 52 | *p* > 0.05^1^ |  |  |  |
| GM-CSF | Tarkowski et al. 2003 | MCI | 56 | 9 months | 31 | 25 | NR |  |  |  |
| IL-1β | Tarkowski et al. 2003 | MCI | 56 | 9 months | 31 | 25 | *p* > 0.05^3^ |  |  |  |
| NPTX2 | Swanson et al. 2016 | MCI | 135 | 24 months | 47 | 82 |  |  |  | F = 4.04  ***p* = 0.047** |
| OPN | Sun et al. 2013 | MCI | 31 | 3 years | 13 | 18 | *p* > 0.05  MCI-AD: BL vs.FUP  ***p* < 0.05** |  |  |  |
| sTNFR1-score (*PCA-score of sTNFR1-related protein = sTNFR1 + sTNFR2 + sVCAM-1 + sICAM-1*) | Hu et al. 2021 | Discovery MCI | 174 | 0 – 60 months | 99 | 75 | Time to progression: High AD-score + Low sTNFR1-score vs. High AD-score + High sTNFR1-score  ***p* = 0.014** | Risk of progression: High AD-score + High sTNFR1-score  HR = 0.541  95%CI = 0.314 - 0.933  ***p* = 0.027** |  |  |
| ysTNFR1-score (*regression-based prediction for sTNFR1 score = sTNFR1 + sTNFR2 + sVCAM-1*) | Hu et al. 2021 | Discovery MCI  Validation MCI | 174  49 | 0 – 60 months  0 – 43 months | 99  18 | 75  31 |  |  | Low ysTNFR1-score + high p-Tau-score vs. High ysTNFR1-score + high p-Tau-score:  ***p* = 0.049**  Low ysTNFR1-score + high p-Tau-score vs. High ysTNFR1-score + high p-Tau-score  ***p* = 0.038** |  |
| TGF-β | Tarkowski et al. 2003 | MCI | 56 | 9 months | 31 | 25 | *p* > 0.05^3^ |  |  |  |
| TNF-α | Tarkowski et al. 2003 | MCI | 56 | 9 months | 31 | 25 | *p* > 0.05^3^ |  |  |  |
| TNFR1 | Zhao et al. 2020 | MCI | 116 | 30.2^^^ months | 64 | 52 |  | Cut-offs = NR  Progression AD-free survival  Total MCI: high vs. low  ***p* = 0.001**  TN-: high vs. low  ***p* = 0.001**  TN+: high vs. low  *p* = 0.539 |  |  |
| TNFR2 | Zhao et al. 2020 | MCI | 116 | 30.2^^^ months | 64 | 52 |  | Cut-offs = NR  Progression AD-free survival:  Total MCI: high vs. low  ***p* < 0.001**  TN-: high vs. low  ***p* = 0.039**  TN+: high vs. low  *p* = 0.085 |  |  |

***Legend***

^*^ Median

^^^  Mean

**^1^** Non-parametric test (e.g. Mann-Whitney U test )

² Scope of conclusion

³ Parametric test (e.g. ANOVA)

***Abbreviations***

95%CI 95% Confidence Interval

APTM Acute partial transverse myelitis

AUC Area under the curve

BL Baseline

CDMS Clinical Definite Multiple Sclerosis

CIS Clinically isolated syndrome

FUP Follow-up

HR Hazard ratio

MCI-AD Progression from MCI to AD

n Number

NPV Negative predictive value

NR Not reported

ON Optic Neuritis

OR Odds ratio

PCA Principle Component Analysis

PPV Positive predictive value

RIS Radiologically isolated syndrome

ROC Receiver operating characteristic

RRMS Relapsing-remitting multiple sclerosis

SD Standard deviation

SE Standard error

sMCI Stable MCI

TN+ Abnormal P-tau and T-tau levels in cerebrospinal fluid

TN- Normal P-tau and T-tau levels in cerebrospinal fluid
